# Net benefit and cost-effectiveness of universal iron-containing multiple micronutrient powders for young children in 78 countries: a microsimulation study

**DOI:** 10.1016/S2214-109X(20)30240-0

**Published:** 2020-08

**Authors:** Sant-Rayn Pasricha, Adrian Gheorghe, Fayrouz Sakr-Ashour, Amrita Arcot, Lynnette Neufeld, Laura E Murray-Kolb, Parminder S Suchdev, Michael Bode

**Affiliations:** Population Health and Immunity Division, Walter and Eliza Hall Institute of Medical Research, Melbourne, VIC, Australia; Department of Medical Biology, University of Melbourne, Melbourne, VIC, Australia; Diagnostic Haematology, The Royal Melbourne Hospital, and Clinical Haematology at The Peter MacCallum Cancer Centre and The Royal Melbourne Hospital, Melbourne, VIC, Australia; Global Health and Development, Department of Infectious Disease Epidemiology, Imperial College London, London, UK; Department of Nutrition and Food Science, University of Maryland, College Park, MD, USA; Department of Nutritional Sciences, Pennsylvania State University, University Park, PA, USA; Global Alliance for Improved Nutrition, Geneva, Switzerland; Department of Nutritional Sciences, Pennsylvania State University, University Park, PA, USA; Nutrition Branch, Centers for Disease Control and Prevention, Atlanta, GA, USA; Department of Pediatrics and Emory Global Health Institute, Emory University, Atlanta, GA, USA; School of Mathematics, Queensland University of Technology, Brisbane, QLD, Australia

## Abstract

**Background:**

Universal home fortification of complementary foods with iron-containing multiple micronutrient powders (MNPs) is a key intervention to prevent anaemia in young children in low-income and middle-income countries. However, evidence that MNPs might promote infection raises uncertainty about whether MNPs give net health benefits and are cost-effective. We aimed to determined country-specific net benefit or harm and cost-effectiveness of universal provision of MNPs to children aged 6 months.

**Methods:**

We developed a microsimulation model to estimate net country-specific disability-adjusted life-years (DALYs), years lived with disability (YLDs), and years of life lost (YLLs) due to anaemia, malaria, and diarrhoea averted (or increased) by provision of a 6-month course of MNPs to children aged 6 months, compared with no intervention, who would be followed up for an additional 6 months (ie, to age 18 months). Anaemia prevalence was derived from Demographic and Health Surveys or similar national surveys, and malaria and diarrhoea incidence were sourced from the Global Burden of Disease Study. Programme and health-care costs were modelled to determine cost per DALY averted (US$). Additionally, we explored the effects of reduced MNP coverage in a sensitivity analysis.

**Findings:**

78 countries (46 countries in Africa, 20 in Asia or the Middle East, and 12 in Latin America) were included in the analysis, and we simulated 5 million children per country. 6 months of universal distribution of daily MNPs, assuming 100% coverage, produced a net benefit (DALYs averted) in 54 countries (24 in Africa, 19 in Asia and the Middle East, 11 in Latin America) and net harm in 24 countries (22 in Africa, one in Asia, and one in Latin America). MNP intervention provided a benefit on YLDs associated with anaemia, but these gains were attenuated and sometimes reversed by increases in YLLs associated with malaria and diarrhoea, reducing the benefits seen for DALYs. In the 54 countries where MNP provision was beneficial, the median benefit was 28·1 DALYs averted per 10 000 children receiving MNPs (IQR 20·6–40·4), and median cost per DALY averted was $3576 (IQR 2474–4918). DALY effects positively correlated with moderate and severe anaemia prevalence in Asia, the Middle East, and Latin America, but correlated inversely in Africa. Suboptimal coverage markedly reduced DALYs averted and cost-effectiveness.

**Interpretation:**

Net health benefits of MNPs vary between countries, are highest where prevalence of moderate and severe anaemia is greatest but infection prevalence is smallest, and are ameliorated when coverage of the intervention is poor. Our data provide country-specific guidance to national policy makers.

**Funding:**

International Union of Nutrition Sciences.

## Introduction

Almost 300 million children younger than 5 years worldwide are anaemic, with most living in low-income and middle-income countries (LMICs).^1^ Dietary iron deficiency is considered to be the major cause of anaemia in children worldwide.^2^ According to WHO, anaemia is classified as a severe public health problem when its prevalence among children younger than 5 years exceeds 40% and a moderate problem when prevalence is between 20% and less than 40%.^3^ WHO recommends that all children aged 6–23 months living where the prevalence of anaemia exceeds 20% should receive home fortification of complementary foods with iron-containing multiple micronutrient powders (MNPs).^4^ MNPs are single-dose sachets of lipid-coated iron combined with other micronutrients that can be mixed into any semi-solid food, enabling a food-based approach for delivering iron to young children. MNPs have become the predominant public health approach to providing iron for young children in LMICs: in 2017, over 16 million children worldwide received MNPs.^5^

However, in the past decade, WHO modelling indicated that only 32% of cases of childhood anaemia in sub-Saharan Africa and 41% in Asia were responsive to iron.^1^ Additionally, iron is also essential for proliferation of pathogenic micro-organisms, and randomised controlled trials in LMICs have raised concerns that iron interventions (including MNPs) could increase infection risk, including clinical incidence of malaria^6^ and diarrhoea.^7,8^

Therefore, when considering whether to implement universal iron interventions for young children, policy makers should balance the expected benefits of anaemia reduction against possible risks of infection. Furthermore, the magnitude of net health benefits should be understood relative to the economic costs of delivering a mass intervention. The net health benefits and resulting cost-effectiveness potentially will vary with the local prevalence and severity of anaemia, malaria control and prevention, burden of other infections, health system characteristics, and health-care costs. Therefore, assessments of risk–benefit and cost-effectiveness should be country specific. To provide policy makers with this information, we used microsimulation modelling to estimate country-specific net health benefit and cost-effectiveness of universal distribution of MNPs to children aged 6 months for a 6-month period compared with no intervention, followed by an additional 6-month post-intervention period, in LMICs where anaemia has been considered an important public health problem.

## Methods

### Study design

**Figure 1:**
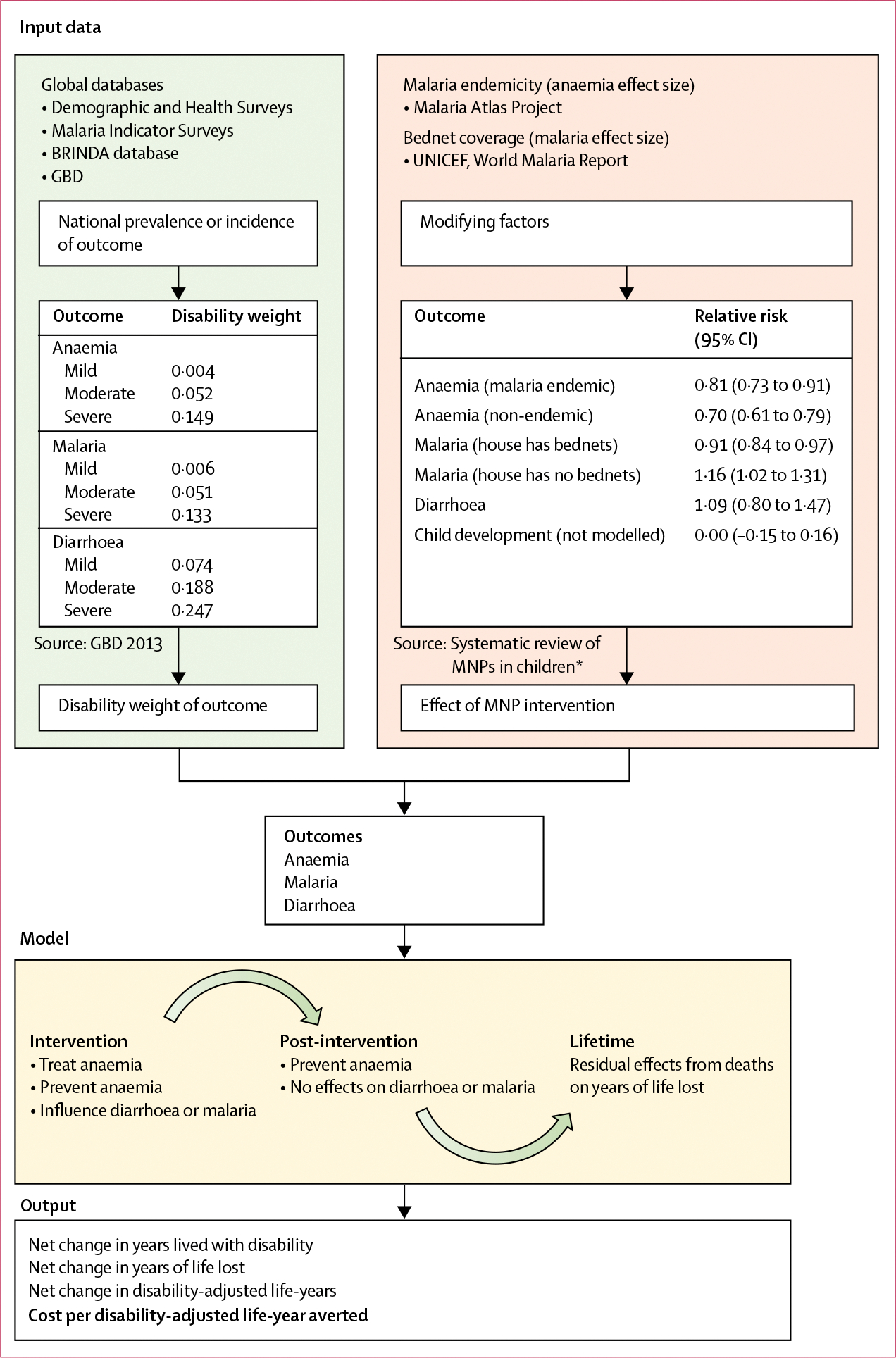
Data sources and modelling approach Summary of the input data sources, analytical approach, and outputs of the model. Effect sizes for MNPs on key outcomes were based on an updated Cochrane systematic review. Disease epidemiology was derived from global epidemiological databases and GBD. Disability weights were adopted from GBD 2013 estimates. The microsimulation approach modelled cohorts of children given MNPs or control from age 6–12 months and followed up to ages 12–18 months, and it ultimately calculated net disability-adjusted life-years averted by the intervention, and cost per disability-adjusted life-year averted. BRINDA=Biomarkers Reflecting Inflammation and Nutritional Determinants of Anemia. GBD=Global Burden of Disease Study. MNPs=multiple micronutrient powders. *See appendix (pp 8–11).

We developed a microsimulation model to estimate disability-adjusted life-years (DALYs) lost to, and direct health-care costs incurred by, anaemia, diarrhoea, and malaria in children aged 6–18 months. We modelled cohorts receiving either a programme of daily MNPs (active intervention group) or no programme (control group). In the active intervention group, we modelled MNPs being given to children aged 6 months, for 6 months of MNP doses, and simulations of outcomes continued until age 18 months (reflecting one cycle of annual supplementation).^4^ We selected outcomes on the basis of WHO priorities used to define guidelines,^4^ their importance in LMICs, and plausibility that MNPs would affect them. Disability weights for diseases were derived from the Global Burden of Disease Study (GBD) 2013.^9^ For each cohort, we calculated years lived with disability (YLDs), years of life lost (YLLs), and DALYs (sum of YLLs and YLDs) incurred by each condition. YLDs were calculated by multiplying the duration of each event by the corresponding disability weight. We calculated deaths on the basis of a fixed, defined country-specific and case-specific mortality rate caused by malaria and diarrhoea. Case-specific mortality rates were derived from GBD 2017 data (number of deaths divided by number of cases; appendix p 16).^10^ YLLs were then calculated as the years between the incident death and the country-specific remaining life expectancy. The primary outcome of the analysis was the number of DALYs (per 10 000 children) averted by MNPs (reduction in DALYs incurred between intervention and the control groups) in each country. We modelled all countries where WHO estimated that the 2011 prevalence of anaemia in children younger than 5 years exceeded 40%, or where MNP intervention programmes (national, subnational, or targeted to particular subgroups) had been piloted or were in progress as of 2013.^11^ We did 5000 simulations of 5000 children per cohort, for each group (ie, intervention and control), per country (5 million children simulated per country), using MATLAB, version 9.6. This study is reported with use of Consolidated Health Economic Evaluation Reporting Standards guidelines.^12^ The analytical approach is summarised in figure 1. Full details of the model are described in the appendix (pp 12–14).

### Disease epidemiology

We extracted the national prevalence of mild, moderate, and severe anaemia^3^ in each age subgroup (6–8 months, 9–11 months, and 12–17 months) from the most recently available Demographic and Health Survey, Malaria Indicator Survey, or national nutrition survey (as of June 30, 2019, from the Biomarkers Reflecting Inflammation and Nutritional Determinants of Anemia working group).^13^ For countries where these data were unavailable (n=19), we derived the age-specific prevalence by adjusting the data to the geographically closest country for which data were available (appendix p 7). For diarrhoea and malaria, we derived clinical incidence from GBD 2017 data.^10^

### Outcomes and effect sizes

Effect sizes for MNPs on anaemia and diarrhoea were based on an updated systematic review of randomised controlled trials that is being used to inform present MNP WHO guidelines (appendix pp 8–11).^14^ We considered that, during intervention, MNPs could both prevent and cure anaemia. Preventive effects of MNPs on anaemia were considered to be sustained for 6 months after the treatment course (ie, from 12–18 months of age). Harmful effects of diarrhoea and malaria were considered to be restricted to the 6-month intervention period (modelling detailed in the appendix, pp 12–14), on the basis of clinical data suggesting that harm from MNPs occurs during treatment.^15^ We stratified anaemia effect size by the malaria endemicity of countries, because the analysis suggested malaria endemicity modifies the efficacy of MNPs on anaemia. Effects of MNPs on clinical malaria were based on a 2016 Cochrane review addressing effects of iron on malaria, which indicated a modification of the effect on the basis of co-provision of malaria control strategies.^16^ Therefore, we modified effects of MNPs on malaria on the basis of co-provision of malaria control, which we defined as whether a child slept under a bednet (using data up to 2015).^17,18^ The model also reduced baseline incidence of malaria by 50% if children slept under a bednet (on the basis of a Cochrane review).^19^ Systematic reviews of randomised controlled trials of iron supplementation and MNPs in young children have not confirmed short-term benefits of iron on child cognitive development,^20,21^ and our analysis of two randomised controlled trials assessing long-term effects of early childhood iron interventions could not identify evidence of benefit (appendix p 11).^22,23^ Therefore, an effect from MNPs on long-term cognition or future earning potential could not be incorporated into the model.

To investigate associations with outcomes from MNPs, we plotted two-way associations between net benefit (in terms of DALYs, YLDs, and YLLs) and baseline prevalence of mild, moderate, and severe anaemia and incidence of malaria or diarrhoea, and calculated correlations using Spearman’s Rho.

### Costs

Costs are expressed in US$ (2015 exchange rate) and include the direct costs of health-care provision—the costs of MNPs (180 doses) and implementation of the programme and the direct costs of treating infection events (country-specific estimates of outpatient and inpatient clinical visits). We estimated MNP costs at $0·03 per dose.^24,25^ We estimated non-drug programme costs at $4·50 per child on the basis of $4 to $5 per-child cost described by the Home Fortification Technical Advisory Group.^26^ We calculated health costs on the basis of presentations for care according to the modelled proportion of mild, moderate, and severe infection cases, and estimated outpatient and inpatient care costs in accordance with WHO Choices data.^27^ Using this information, we estimated the incremental cost per DALY averted. We used probabilistic sensitivity analysis to explore the effect of parameter uncertainty on cost-effectiveness and present results using cost-effectiveness acceptability curves (CEACs) by country, to show the proportion of simulations where the cost per DALY averted was lower than a given cost-effectiveness threshold.^28,29^

### Sensitivity analysis

To model variation in programme coverage, we did sensitivity analyses. We modelled coverage (proportion of children in the intervention group who took a course of MNPs) at 100% (base case), and reduced it to 75%, 50%, and 25%. We modelled non-covered children as still incurring MNP doses (ie, MNP doses would be provided but wasted) and programme costs, but without having either the possible benefits or harms associated with the intervention. We did not model the proportion of doses consumed (adherence).

To model effects of variation in costs of programme delivery between regions, we adopted region-specific multipliers of nutrition programmes proposed by the World Bank (appendix p 15),^30^ and explored their effects on cost-effectiveness and CEACs.

Finally, to estimate the cost-effectiveness of MNPs without incorporating a risk of mortality, we calculated the cost-effectiveness of MNPs per YLD averted.

### Role of the funding source

The funders had no role in study design, data collection, data analysis, data interpretation, or writing of the report. The corresponding author had full access to the data in the study and had final responsibility for the decision to submit for publication.

## Results

78 countries (46 countries in Africa, 20 in Asia or the Middle East, and 12 in Latin America) met the criteria for inclusion in the analysis.

**Figure 2:**
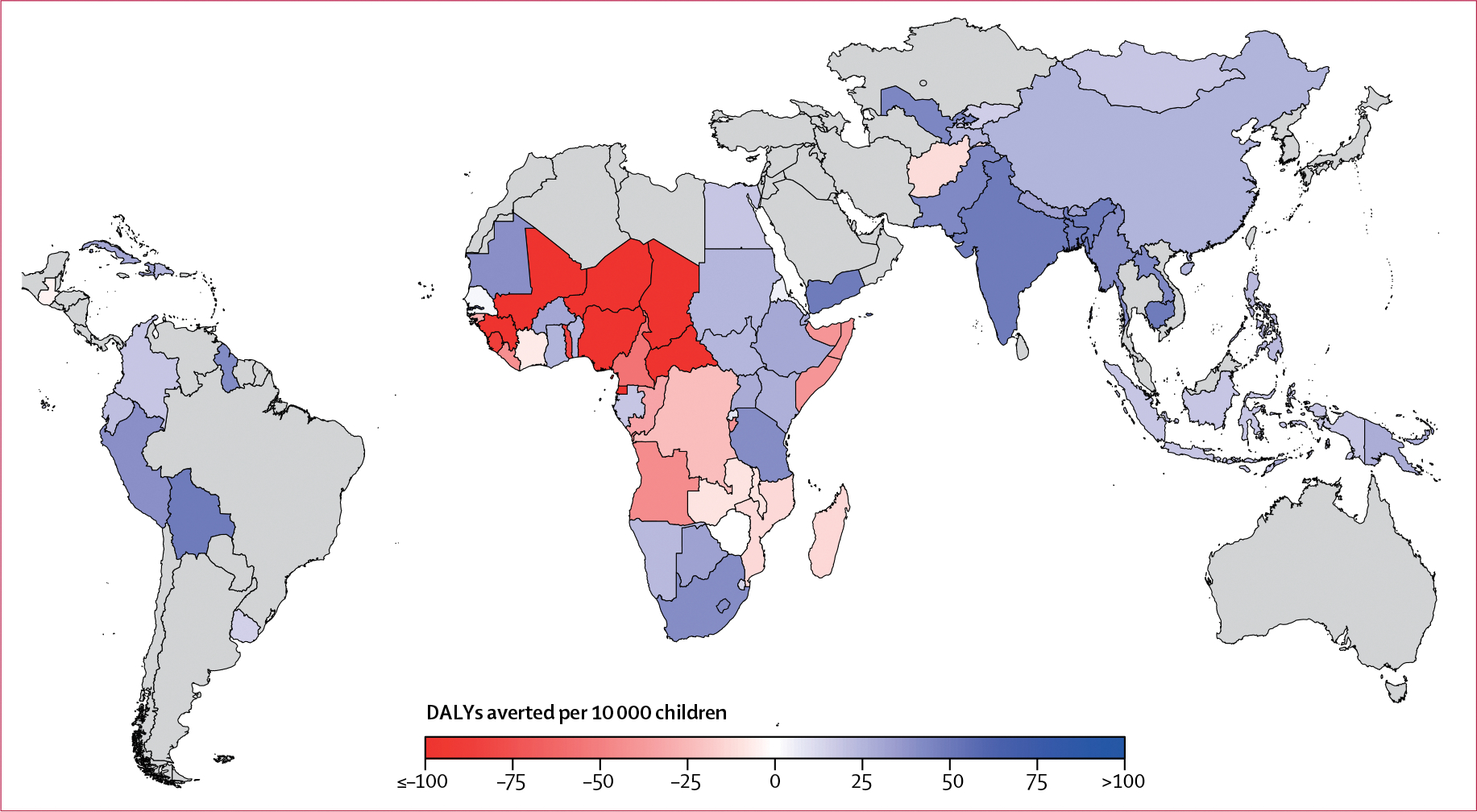
Map of disability-adjusted life-years averted by MNPs Global map showing DALYs per 10 000 children averted through universal delivery of MNPs, assuming 100% coverage. Countries not modelled are shown in grey. DALYs=disability-adjusted life-years. MNPs=multiple micronutrient powders.

6 months of universal distribution of daily MNPs, assuming 100% coverage, produced an average net benefit to health in 54 countries and net harm in 24 countries compared with no intervention (figure 2). In Africa, MNPs were net beneficial in 24 countries modelled and net harmful in 22 countries. In Asia and the Middle East, MNPs were beneficial in all but one of 20 countries modelled; likewise, MNPs were beneficial in 11 of 12 countries modelled in Latin America. The median net effect of MNPs across all 78 countries was 22·7 DALYs averted per 10 000 children (IQR −11·4 to 33·1). By region, the median benefit was 1·5 DALYs averted (−43·0 to 26·0 in Africa, 29·8 (23·6 to 42·6) in Asia and the Middle East, and 31·1 (19·6 to 37·9) in Latin America. Among the 54 countries where MNPs were beneficial, the median benefit was 28·1 DALYs averted per 10 000 children (20·6 to 40·4). Full details on mean net DALYs averted by country are presented in the appendix (p 17).

The top ten countries, where MNPs were most beneficial (DALYs averted per 10 000 children) compared with no intervention, were Yemen (105·2 DALYs averted per 10 000 children, 95% uncertainty interval [UI] 39·9 to 160·2), Bolivia (74·3, 33·5 to 108·7), Bangladesh (63·9, 50·1 to 76·0), Cambodia (58·0, 36·9 to 76·5), India (54·4, 20·3 to 85·0), The Gambia (44·5, 77·1 to 149·2), Uzbekhistan (43·6, 34·7 to 51·0), Pakistan (42·2, −21·9 to 99·6), Laos (42·2, −31·1 to 100·0), and Guyana (41·4, 19·8 to 58·3; appendix p 17). The countries where MNPs caused most harm (DALYs incurred per 10 000 children, expressed as negative DALYs averted) compared with no intervention were Niger (−359·6, 95% UI −999·2 to 229·1), Chad (−220·3, −992·0 to 388·0), Mali (−184·0, −545·0 to 140·8), Nigeria (−165·0, −546·0 to 141·7), Guinea (−150·0, −451·0 to 125·9), Central African Republic (−113·2, −368·9 to 123·3), Equatorial Guinea (−106·7, −320·6 to 97·2), Sierra Leone (−87·9, −324·4 to 146·8), Togo (−83·7, −296·0 to 122·9), and Cameroon (−56·5, −297·2 to 161·0).

**Figure 3:**
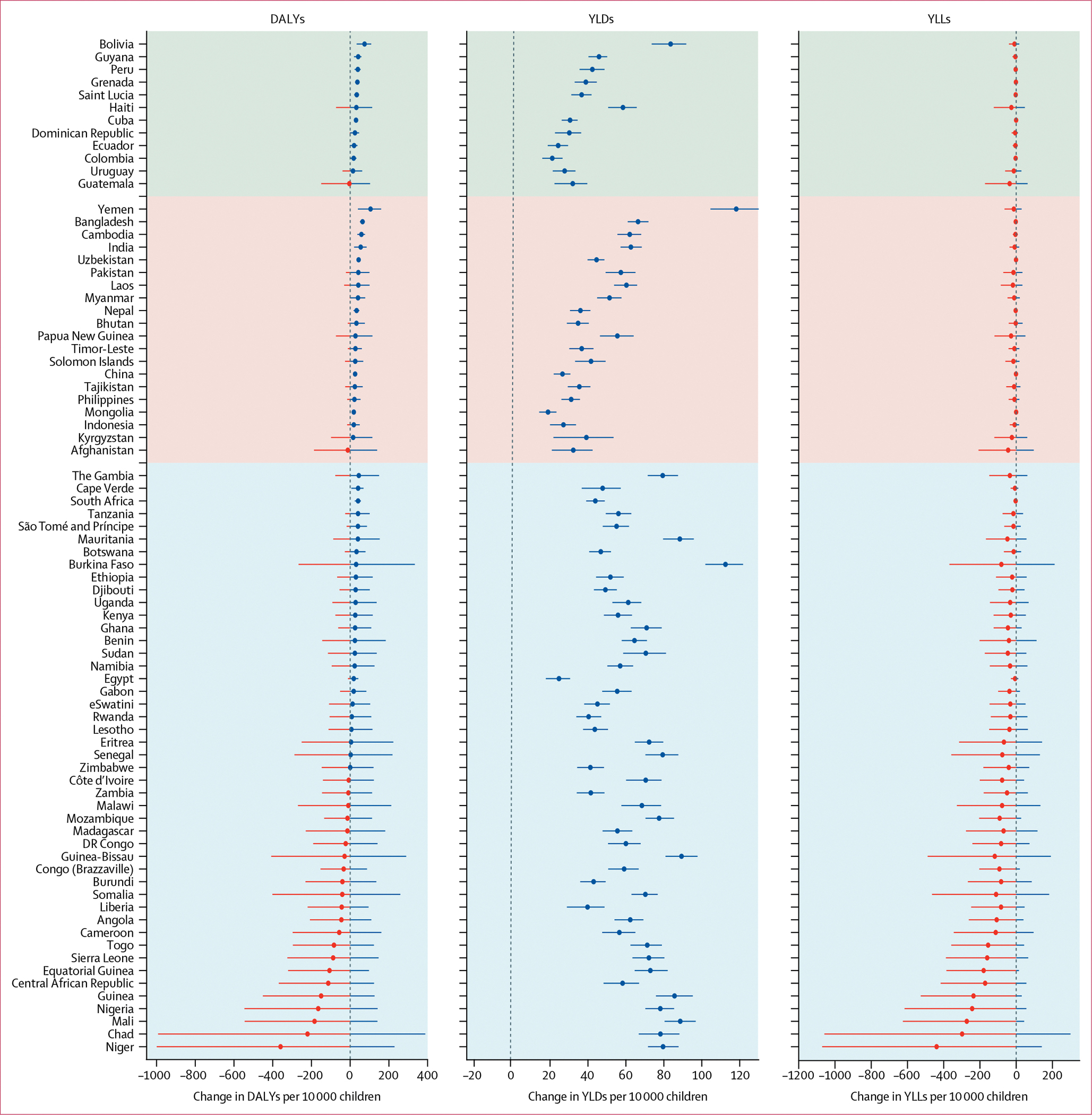
Effects of universal delivery of MNPs on DALYs, YLDs, and YLLs Caterpillar plots showing effects of MNPs on DALYs, YLDs, and YLLs averted per 10 000 children, assuming 100% coverage. Error bars denote 95% uncertainty intervals based on 5000 simulations (percentile method) per country, with 5000 simulated children per arm per simulation. DALYs=disability-adjusted life-years. MNPs=multiple micronutrient powders. YLDs=years lived with disability. YLLs=years of life lost.

We disaggregated the effects of MNPs on DALYs into constitutive YLDs and YLLs (figure 3; appendix p 17). In all countries, MNPs provided a clear positive effect on YLDs compared with no intervention, due to reductions in the prevalence of anaemia. Although the magnitude of this effect was heterogeneous, the median effect was 60·6 YLDs averted per 10 000 children (IQR 49·9–72·7) in Africa, 40·4 (34·2–58·1) in Asia and the Middle East, and 34·3 (29·6–42·2) in Latin America. MNPs also caused a possible excess in the risk of death in some countries, which was minimal in most cases (eg, smaller than 0·5 per 10 000 children in 37 countries). However, in 24 countries (all in sub-Saharan Africa), the risk exceeded 1·0 extra death per 10 000 children and was as high as 7·2 per 10 000 children (in Niger). Because of this excess risk, the net effect of MNPs on YLLs (excess risk of death multiplied by the remainder of that country’s life expectancy) was negative. The effects on DALYs were always smaller than the beneficial effects on YLDs. Overall, in 54 countries, the benefits on YLDs exceeded the risks from infection-related YLLs, hence net DALYs were averted, whereas in 24 countries (22 of which were in Africa), the YLL risks outweighed the YLD benefits, and MNPs caused net DALYs to be incurred. Full details on the effects of MNPs on mean YLDs and YLLs are presented in the appendix (pp 18–117).

In the 54 countries where MNPs were of net benefit, we calculated the cost per DALY averted (appendix p 17). Assuming 100% coverage and uptake of the intervention, median cost-effectiveness in countries where MNPs were beneficial was $3576 per DALY averted (IQR 2474–4918); the median cost-effectivess was $3897 (3101–6980) in Africa, $3136 (2335–4083) in Asia and the Middle East, and $3216 (2736–4783) in Latin America. The ten countries where MNPs were most cost-effective were Yemen ($1041 per DALY averted), Bolivia ($1398), Bangladesh ($1557), Cambodia ($1730), India ($1840), The Gambia ($2240), Uzbekhistan ($2283), Pakistan ($2388), Laos ($2389), and Guyana ($2390; appendix p 17).

When only modelling DALYs associated with anaemia, we found that MNPs were beneficial across all countries (appendix p 118). When we removed risk of death from the model, MNPs were benefical in all countries and cost per DALY averted was lower: median cost per DALY averted was $1741 (IQR 1392–2186) in Africa, $2567 (1735–2969) in Asia and the Middle East, and $3038 (2339–3801) in Latin America (appendix p 119).

**Figure 4:**
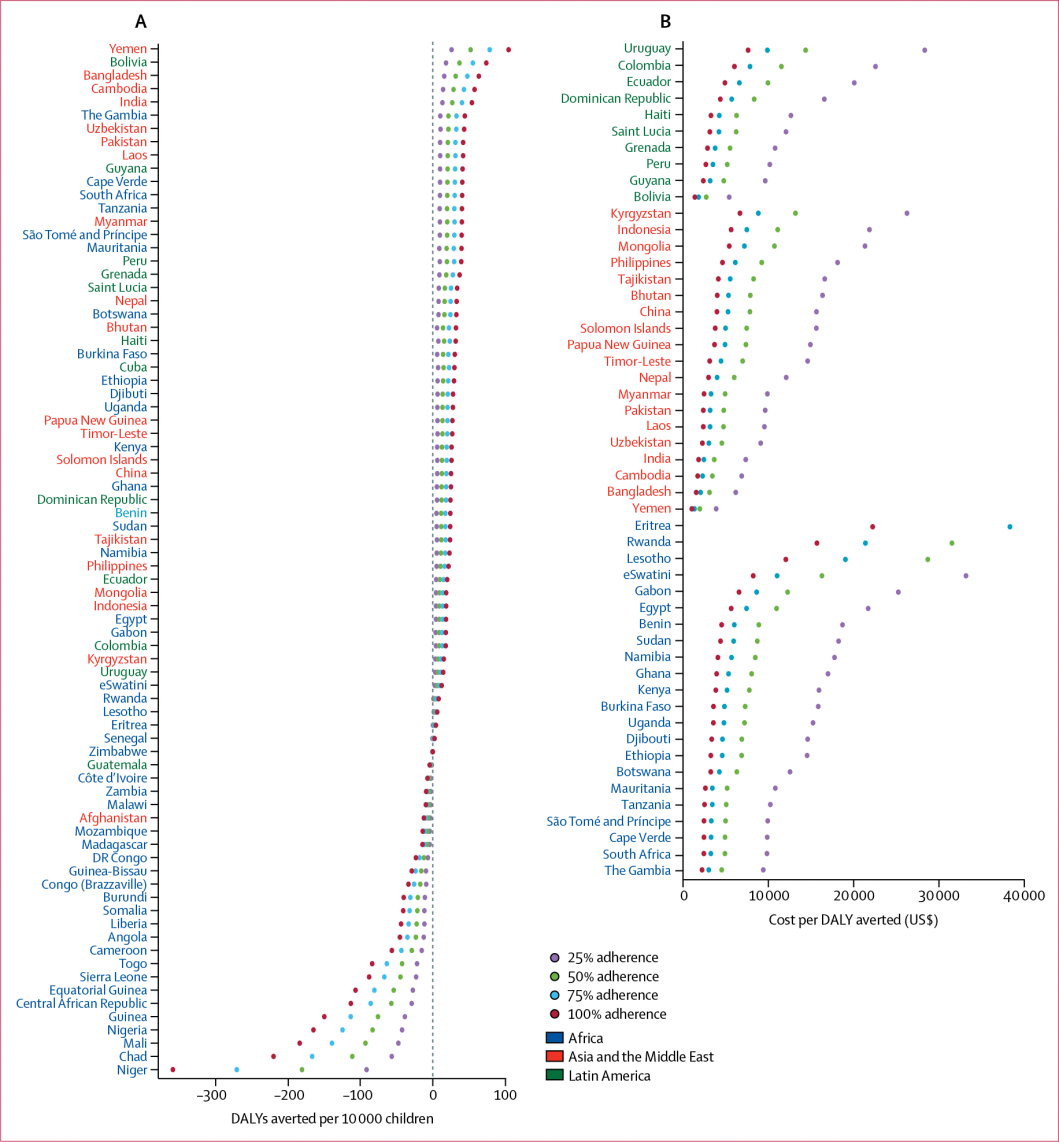
Effects of coverage on net DALYs averted and cost per DALY averted for MNP interventions Effects of reductions in coverage from 100% to 75%, 50%, or 25% on DALYs averted (A) and, in the countries where a net benefit was observed, cost per DALY averted (B). DALYs=disability-adjusted life-years. MNPs=multiple micronutrient powders.

The base case was modelled at 100% coverage and uptake, which might be unrealistic. Therefore, we also modelled coverage rates of 75%, 50%, and 25% (figure 4). In countries where MNPs were beneficial, lower coverage reduced DALYs averted by MNPs (figure 4A) and increased cost per DALY averted (figure 4B). In countries where MNPs were harmful, lower coverage reduced the DALYs incurred.

**Figure 5:**
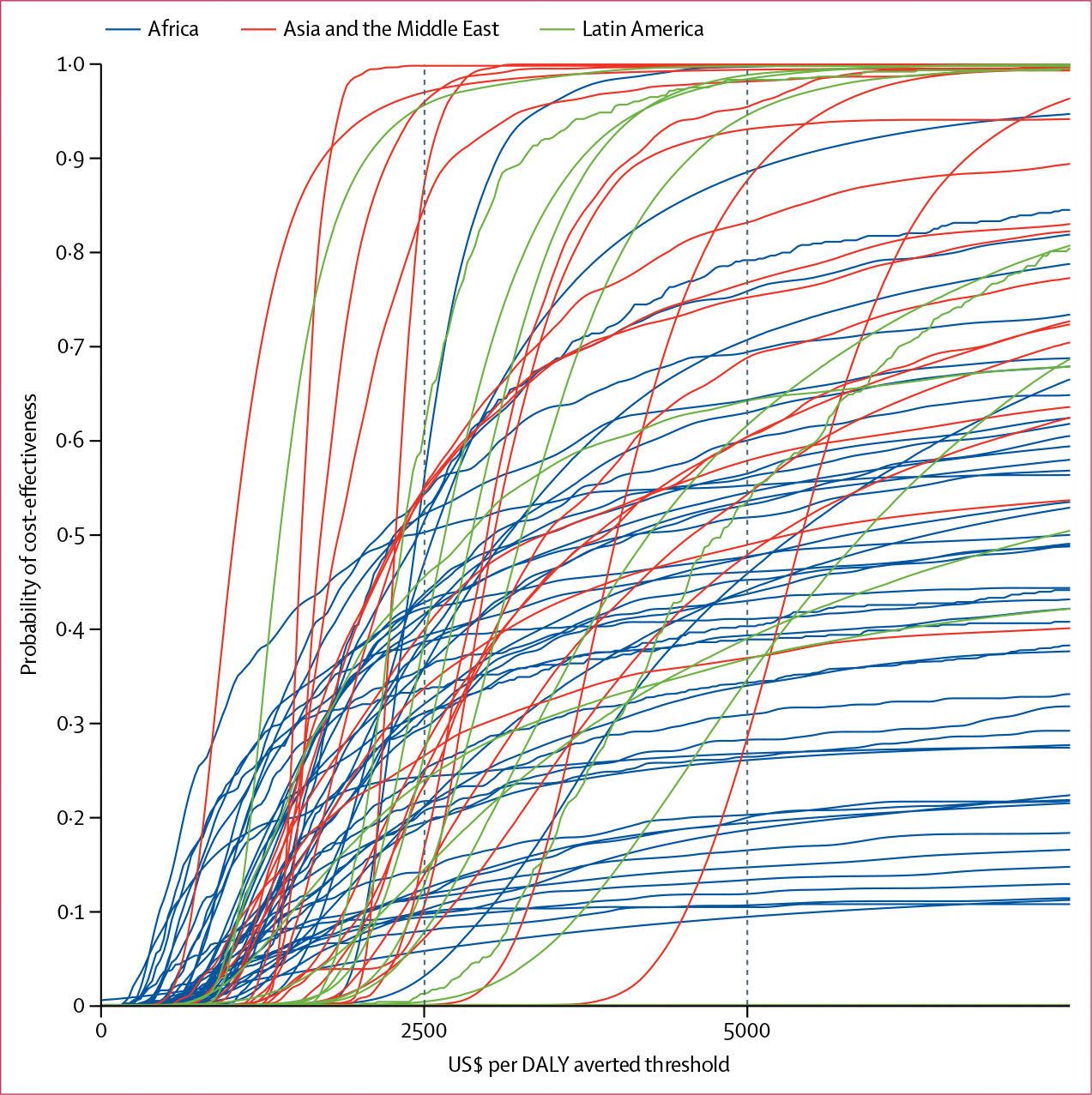
CEACs for countries in each region CEACs for each country in Africa, Asia and the Middle East, and Latin America. Each CEAC plot shows the proportion of simulations (y axis) where MNPs are cost-effective at a particular cost-effectiveness threshold value (x axis). Countries where the cumulative probability does not reach 1·0 are those in which the non-depicted simulations showed a net harm (and hence where cost-effectiveness could not be calculated). CEACs for each country are shown in the appendix (pp 120–22). CEAC=cost-effectiveness acceptability curve.

The CEACs show that for many countries, particularly in Africa, MNPs were unlikely to be cost-effective, even at cost-effectiveness thresholds higher than $5000 per DALY averted (figure 5). Individual CEACs for each country are presented in the appendix (pp 120–22). Effects of variation in programme costs by use of regional multipliers are also presented in the appendix (p 123) and showed an increase in cost per DALY averted in Latin America and central Asia.

We did an ecological analysis at the country level and disaggregated by region to explore baseline factors associated with effects of MNPs. We plotted two-way associations between net benefit (in terms of DALYs, YLDs, and YLLs) and baseline prevalence of mild, moderate, and severe anaemia and incidence of malaria or diarrhoea, and we calculated Spearman’s Rho (appendix pp 124–25). According to our ecological analysis, moderate and severe anaemia prevalence, but not mild anaemia prevalence (appendix p 124), were positively and closely correlated with improvements in YLDs. Moderate and severe anaemia were associated with a negative effect from MNPs on YLLs in Africa but not in other regions, potentially because in Africa, moderate and severe anaemia prevalence were each also positively correlated with incidence of malaria (appendix p 125). As a result, increasing prevalence of moderate and severe anaemia positively correlated with improvements in DALYs in Asia and the Middle East and in Latin America. However, in Africa, the opposite was true, with greater prevalences of moderate and severe anaemia associated with poorer outcomes from MNPs. Detailed reports for each country are included in the appendix (pp 18–117). These reports summarise the input data (eg, disease prevalence, bednet coverage, and case-specific mortality), and present effects of MNPs on anaemia, malaria, and diarrhoea; net effects on DALYs, YLDs, and YLLs; effects on anaemia alone (without considering other infections); cost-effectiveness; and effects of changes in invervention coverage. These reports can be used by national policy makers to inform decisions in their local setting.

## Discussion

Estimates suggest that 54 countries are now delivering MNP programmes.^5,24^ However, concerns exist that MNPs and other iron interventions could exacerbate infection, which might outweigh the benefits from their proven ability to reduce anaemia. We aimed to integrate information on the simultaneous benefits and potential harms of universal MNPs to predict the net effect of these interventions. We found that the magnitude and direction of this net effect was country specific. Beneficial effects of MNPs on YLDs were countered by exacerbations in infection-related YLLs, but the net effect remained positive in most countries. We observed a net harm in 24 countries, including 22 countries in Africa. The cost-effectiveness varied between countries because of the magnitude of effect and health-care costs. Where MNPs were beneficial, suboptimal coverage of programmes markedly attenuated the net benefit and reduced cost-effectiveness.

To corroborate our model, we compared our results to those reported in randomised controlled trials of the effects of MNP provision on anaemia. In Laos, 6 months of MNP provision to children aged 6–52 months reduced anaemia, with a relative risk (RR) of 0·77;^31^ in this country, our model estimated a 26% relative reduction in anaemia prevalence. In China, 6 months of MNP provision reduced absolute anaemia prevalence by 6%;^32^ our model estimated a 7·8% absolute decrease in anaemia in China. In Colombia, 18 months daily MNP provision to children aged 18 months reduced anaemia by RR 0·84;^33^ our model predicted a 25% relative reduction in the prevalence of anaemia in this country. Therefore, our model calculated effect sizes from MNP interventions on anaemia similar to those observed in trials from the past decade.

We aimed to provide a global analysis of net benefit and cost-effectiveness, and thus used standardised data sources across countries. However, the resolution of the input data does not permit us to draw conclusions at subnational or high geospatial resolution scales. We predict average net effects of MNP interventions for entire countries, but some subpopulations might have variation in anaemia, diarrhoea, and malaria epidemiology, where national estimates might not apply. Equally, within countries where we consider MNP interventions to not be cost-effective or to produce net harm, some localities exist where net benefits could be achieved.

We exclusively used effect sizes for our analyses derived from systematic reviews of randomised controlled trials, the best practice approach used by policy makers such as WHO. Previous analyses of the economic benefits of iron interventions have based effect sizes on functional outcomes (eg, two-thirds reductions in mortality or half of an SD effects of iron deficiency anaemia on cognitive development)^34^ by use of observational associations between anaemia and such outcomes.^35^ By restricting use of effect sizes exclusively to those derived from systematic reviews of randomised controlled trials, our analysis represents an estimate of the effects of an intervention (MNPs) rather than a disease (anaemia) on health. Previous analyses did not consider potential risks of interventions, as partly captured with our anaemia-only simulation. Although we originally aimed to incorporate analysis of long-term effects of iron interventions on cognitive development and thus economic productivity, our published meta-analysis of available randomised controlled trials of iron supplementation did not reveal evidence of effects of iron on short-term cognitive development.^20^ We also did a meta-analysis of the two trials^22,23^ that measured effects of iron supplementation in children younger than 2 years on later-life cognitive performance and identified no effect.

Guidelines recommending universal MNP interventions for young children are based on the overall prevalence of anaemia.^36^ However, this prevalence estimate includes mild anaemia, which has among the lowest disability weights for any condition,^9^ makes little contribution to the overall YLDs from anaemia (even when it is highly prevalent), and does not greatly affect the net reduction in DALYs from MNP interventions. Reducing the burden of mild anaemia is of negligible importance to the overall burden of the disease, especially if the therapeutic intervention increases the risk of a potentially lethal infection. In Asia, the Middle East, and Latin America, the prevalence of moderate and severe anaemia, which have much higher disability weights, more closely predict the magnitude of estimated benefit of MNPs on YLDs. In sub-Saharan Africa, positive correlations between anaemia prevalence and malaria mean that the prevalence of moderate and severe anaemia correlated with harm, not benefit, from MNPs; this might partly explain the striking regional variation in net effect size across Africa. In areas without endemic malaria, the prevalence of moderate or severe (rather than overall) anaemia might best guide decisions to implement MNP interventions. MNP distribution where anaemia prevalence is low or less severe will probably have a lesser benefit.

Our analysis is limited by gaps in the input data: specifically, some countries did not have up-to-date anaemia prevalence data. High-quality placebo-controlled randomised controlled trials assessing the effects of MNPs on short and sustained cognitive outcomes in preschool children are needed to definitively address the direction and magnitude of this effect. We derived case-specific mortality rates for malaria and diarrhoea from country-specific global burden of disease incidence and mortality estimates in children aged 1–4 years; case fatality may be higher in younger children, and thus our estimates of risk in children aged 6–12 months might be underestimates and our net benefit an overestimate. Parameter uncertainty and highly stochastic and rare, but impactful, occurrence of deaths caused substantial uncertainty in the YLLs and thus DALYs averted, especially in settings where infections were more highly prevalent.

For countries to optimally allocate scarce health-care resources, considering the cost-effectiveness of interventions is crucial. A 2017 study^37^ suggested that interventions with an incremental cost of $200 per DALY averted or lower could be considered for publicly-funded health care in low-income countries; interventions costing between $200 and $500 could be considered in lower-middle-income countries; and those between $500 and $1000 could be considered in upper-middle-income countries. For example, malaria treatment and addition of pneumococcal, rotavirus, or hepatitis B vaccines to standard schedules cost about $100 per DALY averted, whereas provision of caesarean sections or prevention of cardiovascular disease with drugs approach or exceed $1000 per DALY averted.^37^ Of 29 interventions specifically for children, 26 cost less than $1000 per DALY averted. Our data suggest that MNPs usually exceed $1000 per DALY averted, even if risks are disregarded.

We modelled a constant $4·50 per child in programme costs and explored variation in programme costs by use of regional multipliers, which particularly affect cost-effectiveness in Latin America and central Asia. However, programme costs might be higher in practice: for example, MNP pilot programmes in Uganda suggested that the cost of programme delivery exceeds $27–60 per child.^38^ In other cases, interventions might be fully integrated with existing programmes and thus be cheaper.

The net benefit and cost-effectiveness of MNPs fall with reducing coverage. Use of MNPs requires a complex set of caregiver behaviours and caregiver–child interactions, and many factors detrimentally influence coverage and adherence.^39^ Optimising coverage through community consultation, sensitisation, and education before implementation, and ongoing monitoring and support during intervention, could reimburse the outlay through improved cost-effectiveness.

Iron-containing MNPs are being implemented as a solution to the widespread problem of anaemia in young children. Our findings support policy makers and donors tasked with prioritising the settings in which to deliver these interventions, assist country-level decision makers to choose whether to opt for an MNP intervention programme among the range of health interventions available, and offer a framework for researchers to define and create environments that might enable beneficial outcomes from these interventions.

## Supplementary Material

supplementary material

## Research in context

### Evidence before this study

Anaemia affects 300 million children younger than 5 years worldwide, mainly in low-income and middle-income countries, and is a leading global cause of years lived with disability. Multiple micronutrient powders (MNPs) are a key WHO-recommended intervention for reducing anaemia in infants in low-income and middle-income countries but, because these supplements can also exacerbate infection, their net benefit and overall cost-effectiveness is unknown. We searched MEDLINE with no language restrictions for articles published between Jan 1, 1966, and Nov 1, 2019, using the search terms “(anaemia) AND (iron or (multiple micronutrient powders)) AND (children) AND (health economics OR cost effectiveness OR DALYs)”, and supplemented this search with known references or reports on this topic. We found that previous analyses of the economic benefits of iron interventions based effect sizes on functional outcomes that were derived from assumptions or observational studies, rather than from systematic reviews. No previous economic analyses had considered potential risks of iron interventions. Therefore, these analyses report highly favourable benefits and health economic outcomes of MNP interventions.

### Added value of this study

Our study systematically integrated the best available information on evidence of effect of MNP intervention, country-level disease epidemiology, direct health-care costs, and interacting factors such as bednet coverage. By using effect sizes exclusively derived from systematic reviews of interventional trials, our data comprised an evidence-based synthesis of the effects of an intervention rather than a disease, which is the information that policy makers need. We provide detailed country-level reports that can be directly used by national health officers to inform policy decisions.

### Implications of all the available evidence

Our findings indicate that, despite the risk of infection, MNPs are net beneficial in most—but not all—countries. However, this benefit is modest in many settings and, ultimately, some countries might not consider the intervention cost-effective. The prevalence of moderate and severe anaemia corresponded with net benefit in all regions except Africa where, because of the high prevalence of malaria, the opposite was true. We provide clear modelling of the effects of diminished coverage on the net effect and cost-effectiveness of an MNP intervention, which provides a clear rationale for programme implementers to ensure that attention is placed on optimising uptake. Our findings assist country-level decision makers to choose whether to invest in an MNP intervention programme, and they offer researchers a framework to define environments where beneficial outcomes from these interventions might be expected.

## References

[1] WHO. The global prevalence of anaemia in 2011. Geneva: World Health Organization, 2015.

[2] Kassebaum NJ, Jasrasaria R, Naghavi M, A systematic analysis of global anemia burden from 1990 to 2010. Blood 2014; 123: 615–24. 24297872 10.1182/blood-2013-06-508325 PMC3907750

[3] WHO. Haemoglobin concentrations for the diagnosis of anaemia and assessment of severity. Geneva: World Health Organization, 2011.

[4] WHO. Use of multiple micronutrient powders for point-of-use fortication of foods consumed by infants and young children aged 6–23 months and children aged 2–12 years. Geneva: World Health Organization, 2016. 28079999

[5] UNICEF. NutriDash 2.0. Multiple micronutrient powders 2019. https://www.unicefnutridash.org/surveyreport/3 (accessed Aug 14, 2019).

[6] Sazawal S, Black RE, Ramsan M, Effects of routine prophylactic supplementation with iron and folic acid on admission to hospital and mortality in preschool children in a high malaria transmission setting: community-based, randomised, placebo-controlled trial. Lancet 2006; 367: 133–43. 16413877 10.1016/S0140-6736(06)67962-2

[7] Soofi S, Cousens S, Iqbal SP, Effect of provision of daily zinc and iron with several micronutrients on growth and morbidity among young children in Pakistan: a cluster-randomised trial. Lancet 2013; 382: 29–40. 23602230 10.1016/S0140-6736(13)60437-7

[8] Jaeggi T, Kortman GA, Moretti D, Iron fortification adversely affects the gut microbiome, increases pathogen abundance and induces intestinal inflammation in Kenyan infants. Gut 2015; 64: 731–42. 25143342 10.1136/gutjnl-2014-307720

[9] Salomon JA, Haagsma JA, Davis A, Disability weights for the Global Burden of Disease 2013 study. Lancet Glob Health 2015; 3: e712–23. 26475018 10.1016/S2214-109X(15)00069-8

[10] Global Burden of Disease Collaborative Network. Global Burden of Disease Study 2017 (GBD 2017) results. Seattle,WA: Institute for Health Metrics and Evaluation, 2018.

[11] Jefferds ME, Irizarry L, Timmer A, Tripp K. UNICEF-CDC global assessment of home fortification interventions 2011: current status, new directions, and implications for policy and programmatic guidance. Food Nutr Bull 2013; 34: 434–43. 24605694 10.1177/156482651303400409 PMC4547468

[12] Husereau D, Drummond M, Petrou S, Consolidated Health Economic Evaluation Reporting Standards (CHEERS) statement. BMJ 2013; 346: f1049. 23529982 10.1136/bmj.f1049

[13] Suchdev PS, Namaste SM, Aaron GJ, Overview of the Biomarkers Reflecting Inflammation and Nutritional Determinants of Anemia (BRINDA) project. Adv Nutr 2016; 7: 349–56. 26980818 10.3945/an.115.010215 PMC4785469

[14] De-Regil LM, Suchdev PS, Vist GE, Walleser S, Pena-Rosas JP. Home fortification of foods with multiple micronutrient powders for health and nutrition in children under two years of age. Cochrane Database Syst Rev 2011; 9: CD008959. 10.1002/14651858.CD008959.pub2 21901727

[15] Zlotkin S, Newton S, Aimone AM, Effect of iron fortification on malaria incidence in infants and young children in Ghana: a randomized trial. JAMA 2013; 310: 938–47. 24002280 10.1001/jama.2013.277129

[16] Neuberger A, Okebe J, Yahav D, Paul M. Oral iron supplements for children in malaria-endemic areas. Cochrane Database Syst Rev 2016; 2: CD006589. 26921618 10.1002/14651858.CD006589.pub4 PMC4916933

[17] UNICEF. Malaria data—child health coverage. https://data.unicef.org/resources/dataset/malaria/ (accessed June 1, 2016).

[18] WHO. World malaria report 2014. Geneva: World Health Organization, 2015.

[19] Lengeler C Insecticide-treated bed nets and curtains for preventing malaria. Cochrane Database Syst Rev 2004; 2: CD000363. 10.1002/14651858.CD000363.pub2 15106149

[20] Pasricha S-R, Hayes E, Kalumba K, Biggs B-A. Effect of daily iron supplementation on health in children aged 4—23 months: a systematic review and meta-analysis of randomised controlled trials. Lancet Glob Health 2013; 1: e77–86. 25104162 10.1016/S2214-109X(13)70046-9

[21] Sachdev H, Gera T, Nestel P. Effect of iron supplementation on mental and motor development in children: systematic review of randomised controlled trials. Public Health Nutr 2005; 8: 117–32. 15877905 10.1079/phn2004677

[22] Pongcharoen T, DiGirolamo AM, Ramakrishnan U, Winichagoon P, Flores R, Martorell R. Long-term effects of iron and zinc supplementation during infancy on cognitive function at 9 y of age in northeast Thai children: a follow-up study. Am J Clin Nutr 2011; 93: 636–43. 21270383 10.3945/ajcn.110.002220

[23] Murray-Kolb LE, Khatry SK, Katz J, Preschool micronutrient supplementation effects on intellectual and motor function in school-aged Nepalese children. Arch Pediatr Adolesc Med 2012; 166: 404–10. 22566538 10.1001/archpediatrics.2012.37

[24] UNICEF Supply Division. Multiple micronutrient powder supply and market outlook. 2016. https://www.unicef.org/supply/media/566/file/Multiple%20micronutrient%20powder%20(MNP)%20supply%20and%20market%20outlook.pdf x (accessed Aug 4, 2017).

[25] UNICEF, WHO. Sources and prices of selected medicines for children—including therapeutic food, dietary vitamin and mineral supplementation. Geneva: World Health Organization, 2010.

[26] De Pee Saskia, Flores-Ayala Rafael, Van Hees Joris, , eds. Introduction: Home Fortification Technical Advisory Group. Geneva: Home Fortification Technical Advisory Group, 2013.

[27] WHO. Tables of costs and prices used in WHO-CHOICE analysis. http://www.who.int/choice/costs/en/ (accessed June 1, 2018).

[28] Fenwick E, O’Brien BJ, Briggs A. Cost-effectiveness acceptability curves—facts, fallacies and frequently asked questions. Health Economics 2004; 13: 405–15. 15127421 10.1002/hec.903

[29] Briggs AH, Weinstein MC, Fenwick EA, Model parameter estimation and uncertainty: a report of the ISPOR-SMDM Modeling Good Research Practices Task Force—6. Value Health 2012; 15: 835–42. 22999133 10.1016/j.jval.2012.04.014

[30] Horton S, Shekar M, McDonald C, Mahal A, Brooks JK. Scaling up nutrition—what will it cost? Washington, DC: The World Bank, 2010.

[31] Kounnavong S, Sunahara T, Mascie-Taylor CG, Effect of daily versus weekly home fortification with multiple micronutrient powder on haemoglobin concentration of young children in a rural area, Lao People’s Democratic Republic: a randomised trial. Nutr J 2011; 10: 129. 22111770 10.1186/1475-2891-10-129 PMC3266642

[32] Luo R, Yue A, Zhou H, The effect of a micronutrient powder home fortification program on anemia and cognitive outcomes among young children in rural China: a cluster randomized trial. BMC Public Health 2017; 17: 738. 28946866 10.1186/s12889-017-4755-0 PMC5613507

[33] Andrew A, Attanasio O, Fitzsimmons E, Rubio-Codina M. Why is multiple micronutrient powder ineffective at reducing anaemia among 12–24 month olds in Colombia? Evidence from a randomised controlled trial. SSM Popul Health 2016; 2: 95–104. 29349132 10.1016/j.ssmph.2016.02.004 PMC5757801

[34] Horton S The economics of food fortification. J Nutr 2006; 136: 1068–71. 16549479 10.1093/jn/136.4.1068

[35] Stoltzfus RJ. Mullany LBR. Iron deficiency anaemia. In: Ezzati MLA, Rodgers A, Murray CJL, eds. Comparative quantification of health risks: global and regional burden of disease attributable to selected major risk factors. Geneva: World Health Organization, 2004: 163–209.

[36] WHO. Daily iron supplementation in children 6–23 months of age. 2016. http://www.who.int/elena/titles/guidance_summaries/iron_children/en/ (accessed July 11, 2016).

[37] Horton S, Gelband H, Jamison D, Levin C, Nugent R, Watkins D. Ranking 93 health interventions for low- and middle-income countries by cost-effectiveness. PLoS One 2017; 12: e0182951. 28797115 10.1371/journal.pone.0182951 PMC5552255

[38] Namaste S, Richardson B, Ssebiryo F, Kantuntu D, Vosti S, D’Agostino A. Comparing the effectiveness and cost-effectiveness of facility- versus community-based distribution of micronutrient powders in rural Uganda. IUNS 21st International Congress of Nutrition; Buenos Aires; Oct 15–20, 2017.

[39] Tumilowicz A, Schnefke CH, Neufeld LM, Pelto GH. Toward a better understanding of adherence to micronutrient powders: generating theories to guide program design and evaluation based on a review of published results. Curr Dev Nutr 2017; 1: e001123. 29955708 10.3945/cdn.117.001123 PMC5998355

