# Supplementary material for: Net benefit and cost-effectiveness of universal iron-containing multiple micronutrient powders for young children in 78 countries: a microsimulation study
Source: Lancet Glob Health. Author manuscript; Available in PMC 2022 Oct 31. (PMC9620416; doi:10.1016/S2214-109X(20)30240-0)
Supplement: supplementary material [file NIHMS1841695-supplement-supplementary_material.pdf]

THE LANCET
Global Health

Supplementary appendix

This appendix formed part of the original submission and has been peer reviewed. We post it as supplied by the authors.

Supplement to: Pasricha S-R, Gheorghe A, Sakr-Ashour F, et al. Net benefit and cost-effectiveness of universal iron-containing multiple micronutrient powders for young children in 78 countries: a microsimulation study. *Lancet Glob Health* 2020; **8:** e1071–80.

Net benefit and cost-effectiveness of universal iron-containing multiple micronutrient powders for young children in 78 countries: a microsimulation study: Supplementary Appendix

Supplementary Methods 2
Analytic overview 2
Countries 2
Disease epidemiology 2
Anaemia 3
Diarrhoea 8
Malaria 8
Intervention effect sizes 8
Summary of effect sizes used in the model 8
Comparison of MNP versus no intervention or placebo on anaemia by malaria endemicity in the study area 10
Comparison of MNP versus no intervention or placebo on diarrhoea 10
Effects on cognition 11
Outcomes 11
Modelling 12
Modelling of anaemia 12
Associations with MNP effects on disease burden 13
Model validation 13
Uncertainty 14
Costs 14
Cost Effective Acceptability Curves (CEACs) 15
Sensitivity analysis 15
Supplementary Data 16
Table S1 Case-specific mortality for diarrhoea and malaria for each country included in the analysis, derived from Global Burden of Disease data. 16
Table S2 Effect of MNP on DALYs, YLDs and YLLs in each country modelled, at 100%, 75%, 50% and 25% coverage. 17
Table S3 Detailed reports on benefit risk and cost per DALY averted for each country modelled, in each region. 18
Africa 18
Asia and the Middle East 76
Latin America 102
Figure S1 Anaemia-only model of effects of universal MNP interventions on DALYs 118
Figure S2 Cost per YLD averted for provision of MNPs adjusted by coverage 119
Figure S3 Cost Effectiveness Acceptability Curves for Africa 120
Figure S4 Cost Effectiveness Acceptability Curves for Asia and The Middle East 121
Figure S5 Cost Effectiveness Acceptability Curves for Latin America 122
Figure S6 Cost per DALY averted using regional multipliers for programme costs 123
Figure S7 Correlation matrix of associations between YLDs/ YLLs/ DALYs averted by MNPs and prevalence of anaemia (mild/ moderate/ severe/ moderate+severe), incidence of malaria and incidence of diarrhoea. 124
Figure S8 Correlation matrix of associations between prevalence of anaemia (mild/ moderate/ severe/ moderate+severe), incidence of malaria and incidence of diarrhoea. 125
References 126

Supplementary Methods

Analytic overview

We developed a microsimulation model to estimate the Disability Adjusted Life Years (DALYs) lost due to, and costs incurred by, anaemia, diarrhoea and malaria between 6 and 18 months of age. These outcomes were selected based on WHO priorities used to define guidelines,[1,2] perceived or known effects from iron on these outcomes, ability to parameterise them using this approach, and their importance in high anaemia burden countries.

We modeled individuals in control cohorts (no intervention) and individuals in cohorts receiving MNPs. We followed children from birth, applied interventions from the ages of six to 12 months, measured DALYs incurred by each condition (above) by age eighteen months, and compared total DALYs incurred due to these causes between intervention and control groups at this timepoint. We estimated the incidence of death in the event of each condition according to the country-specific case-fatality rate, and calculated Years of Life Lost based on the interval between age at death and life expectancy. In addition, we calculated the effect of both interventions on health costs, based on increased or reduced presentations for care. Finally, we used these two estimates to estimate the overall cost per DALY averted (differences in DALYs incurred).

The model is illustrated in Supplementary Figure 1. The analysis is reported following the CHEERS guidelines.[3]

Countries

We applied this microsimulation model to countries where the latest WHO estimates indicate that prevalence of anaemia in under-5 children exceeds 40% (and hence is considered a ‘severe’ public health problem[4]), or where MNP intervention programmes (targeted, pilot, subnational or national) were introduced or in progress as of 2013.[5] This latter inclusion thus meant that in some cases we modeled implementation in an entire country whereas a programme may only be targeted to a particular high risk subpopulation within that country. Supplementary Table 1 summarises the raw data used to run the model for each country.

Disease epidemiology

We mined publically-available datasets to extract country-specific estimates of disease epidemiology.

Anaemia

Prevalence of mild, moderate and severe anaemia (categorized based on haemoglobin cut-offs as mild: 10.9-10g/dL, moderate: 9.9-7g/dL, and severe: <7g/dL)[4] for each age group (usually 6-11 months, 12-18 months, 19-23 months, 24-35 months, 36-47 months and 48-59 months) was derived from the most recent available Demographic and Health Survey (DHS) or Malaria Indicator Survey (MIS)), or national nutrition survey compiled by the BRINDA working group (www.BRINDA-nutrition.org).

Afghanistan	Ministry of Public Health, UNICEF and Aga Khan University. 2013 Afghanistan National Nutrition Survey. Kabul: UNICEF Afghanistan.
Angola	Instituto Nacional de Estatística (INE), Ministério da Saúde (MINSA), Ministério do Planeamento e do Desenvolvimento Territorial (MINPLAN) e ICF. 2017. *Inquérito de Indicadores Múltiplos e de Saúde em Angola 2015-2016*. Luanda, Angola e Rockville, Maryland, EUA: INE, MINSA, MINPLAN e ICF.
Bangladesh	National Institute of Population Research and Training (NIPORT), Mitra and Associates, and ICF International. 2013. Bangladesh Demographic and Health Survey 2011. Dhaka, Bangladesh and Calverton, Maryland, USA: NIPORT, Mitra and Associates, and ICF International.
Benin	Institut National de la Statistique et de l'Analyse Économique (INSAE) et ICF International, 2013. Enquête Démographique et de Santé du Bénin 2011-2012. Calverton, Maryland, USA : INSAE et ICF International.
Bolivia	MINISTERIO DE SALUD Y DEPORTES and Measure DHS. Encuesta Nacional de Demografía y Salud (ENDSA 2008). Octubre 2009. La Paz, Bolivia, and Calverton, Maryland
Burkina Faso	Institut National de la Statistique et de la Démographie (INSD) et ICF International, 2012. Enquête Démographique et de Santé et à Indicateurs Multiples du Burkina Faso 2010. Calverton, Maryland, USA : INSD et ICF International.
Burundi	Ministère à la Présidence chargé de la Bonne Gouvernance et du Plan [Burundi] (MPBGP), Ministère de la Santé Publique et de la Lutte contre le Sida [Burundi] (MSPLS), Institut de Statistiques et d'Études Économiques du Burundi (ISTEEBU), et ICF. 2017. *Troisième Enquête Démographique et de Santé*. Bujumbura, Burundi : ISTEEBU, MSPLS, et ICF.
Cambodia	National Institute of Statistics, Directorate General for Health, and ICF International, 2015. Cambodia Demographic and Health Survey 2014. Phnom Penh, Cambodia, and Rockville, Maryland, USA: National Institute of Statistics, Directorate General for Health, and ICF International.
Cameroon	Institut National de la Statistique (INS) et ICF. International. 2012. Enquête Démographique et de Santé et à Indicateurs Multiples du Cameroun 2011. Calverton, Maryland, USA : INS et ICF International.
Cape Verde	Instituto Nacional de Estatística (INE) [Cabo Verde], Ministério da Saúde, e Macro International. 2008. Segundo Inquérito Demográfico e

	de Saúde Reprodutiva, Cabo Verde, IDSR-II, 2005. Calverton, Maryland, USA: INE
Colombia	Minesterio de Proteccion Social, Instituto Nacional de Salud. 2011. Encuesta Nacional de la Situacion Nutricional en Colombia 2010-ENSIN. Bogota D.C.
Congo	Centre Nationale de la Statistique et des Études Économiques (CNSEE) [Congo] et ICF International. 2013 Enquête Démographique et de Santé du Congo (EDSC-II) 2011-2012. Calverton, Maryland, USA : CNSEE et ICF International.
Cote D'Ivoire	Institut National de la Statistique (INS) et ICF International. 2012. Enquête Démographique et de Santé et à Indicateurs Multiples de Côte d'Ivoire 2011-2012. Calverton, Maryland, USA : INS et ICF International.
DRC	Ministère du Plan et Suivi de la Mise en œuvre de la Révolution de la Modernité (MPSMRM), Ministère de la Santé Publique (MSP) et ICF International, 2014. Enquête Démographique et de Santé en République Démocratique du Congo 2013-2014. Rockville, Maryland, USA : MPSMRM, MSP et ICF International.
Egypt	Ministry of Health and Population [Egypt], El-Zanaty and Associates [Egypt], and ICF International. 2015. Egypt Demographic and Health Survey 2014. Cairo, Egypt and Rockville, Maryland, USA: Ministry of Health and Population and ICF International.
Equitorial Guinea	Ministerio de Sanidad y Bienestar Social, Ministerio de Economía, Planificación e Inversiones Públicas, e ICF International. 2012. Encuesta Demográfica y de Salud (EDSGE-I) 2011. Calverton, Maryland, USA
Ethiopia	Central Statistical Agency (CSA) [Ethiopia] and ICF. 2016. Ethiopia Demographic and Health Survey 2016. Addis Ababa, Ethiopia, and Rockville, Maryland, USA: CSA and ICF.
Gabon	Direction Générale de la Statistique (DGS) et ICF International. 2013. Enquête Démographique et de Santé du Gabon 2012. Calverton, Maryland, et Libreville, Gabon : DGS et ICF International.
Ghana	Ghana Statistical Service (GSS), Ghana Health Service (GHS), and ICF International. 2015. Ghana Demographic and Health Survey 2014. Rockville, Maryland, USA: GSS, GHS, and ICF International.
Guatemala	Ministerio de Salud Pública y Asistencia Social (MSPAS), Instituto Nacional de Estadística (INE), ICF International, 2017. Encuesta Nacional de Salud Materno Infantil 2014-2015. Informe Final. Guatemala, MSPAS/INE/ICF
Guinea	Institut National de la Statistique Ministère du Plan Conakry, Guinée and MEASURE DHS, ICF International Calverton, Maryland, U.S.A. ENQUÊTE DEMOGRAPHIQUE ET DE SANTÉ ET À INDICATEURS MULTIPLES (EDS-MICS 2012). Novembre 2013.
Guyana	Ministry of Health (MOH), Bureau of Statistics (BOS), and ICF Macro. 2010. Guyana Demographic and Health Survey 2009. Georgetown, Guyana: MOH, BOS, and ICF Macro.
Haiti	Cayemittes, Michel, Michelle Fatuma Busangu, Jean de Dieu Bizimana, Bernard Barrère, Blaise Sévère, Viviane Cayemittes et Emmanuel Charles. 2013. Enquête Mortalité, Morbidité et Utilisation des Services, Haïti, 2012. Calverton, Maryland, USA : MSPP, IHE et ICF International.

India	International Institute for Population Sciences (IIPS) and ICF. 2017. National Family Health Survey (NFHS-4), 2015-16: India. Mumbai: IIPS.
Kenya	National Malaria Control Programme (NMCP), Kenya National Bureau of Statistics (KNBS), and ICF International. 2016. Kenya Malaria Indicator Survey 2015. Nairobi, Kenya, and Rockville, Maryland, USA: NMCP, KNBS, and ICF International.
Kyrgyz Republic	National Statistical Committee of the Kyrgyz Republic (NSC), Ministry of Health [Kyrgyz Republic], and ICF International. 2013. Kyrgyz Republic Demographic and Health Survey 2012. Bishkek, Kyrgyz Republic, and Calverton, Maryland, USA: NSC, MOH, and ICF International.
Laos	Ministry of Planning and Investment, Ministry of Health and UNICEF. 2008. National Maternal and Child Nutrition Survey (MICS3-NNS) Report, The Lao PDR, 2006.
Lesotho	Ministry of Health [Lesotho] and ICF International. 2016. Lesotho Demographic and Health Survey 2014. Maseru, Lesotho: Ministry of Health and ICF International.
Liberia	UNICEF. Liberia National Micronutrient Survey 2011—Selected preliminary findings. Monrovia, Liberia: UNICEF, Liberia Institute of Statistics, 2011.
Madagascar	Institut National de la Statistique (INSTAT), Programme National de lutte contre le Paludisme (PNLP), Institut Pasteur de Madagascar (IPM) et ICF International. 2016. *Enquête sur les Indicateurs du Paludisme 2016*. Calverton, MD, USA : INSTAT, PNLP, IPM et ICF International.
Malawi	National Statistical Office (NSO) [Malawi] and ICF. 2017. Malawi Demographic and Health Survey 2015-16. Zomba, Malawi, and Rockville, Maryland, USA. NSO and ICF.
Mali	Cellule de Planification et de Statistique (CPS/SSDSPF), Institut National de la Statistique (INSTAT/MPATP), INFO-STAT et ICF International, 2014. Enquête Démographique et de Santé au Mali 2012-2013. Rockville, Maryland, USA : CPS, INSTAT, INFO-STAT et ICF International.
Mongolia	Lander, R. L., Enkhjargal, T., Batjargal, J., Bailey, K. B., Diouf, S., Green, T. J., Gibson, R. S. 2008. Multiple micronutrient deficiencies persist during early childhood in Mongolia. Asia Pacific Journal of Clinical Nutrition, 17(3), 429-440
Mozambique	Ministério da Saúde (MISAU), Instituto Nacional de Estatística (INE), e ICF, 2015. Inquérito de Indicadores de Imunização, Malária e HIV/SIDA em Moçambique 2015. Maputo, Moçambique. Rockville, Maryland, EUA: INS, INE, e ICF.
Myanmar	Ministry of Health and Sports - MoHS/Myanmar and ICF. 2017. Myanmar Demographic and Health Survey 2015-16. Nay Pyi Taw, Myanmar: MoHS and ICF.
Namibia	The Nambia Ministry of Health and Social Services (MoHSS) and ICF International. 2014. The Namibia Demographic and Health Survey 2013. Windhoek, Namibia, and Rockville, Maryland, USA: MoHSS and ICF International.
Nepal	Ministry of Health and Population (MOHP) [Nepal], New ERA, and ICF International Inc. 2012. Nepal Demographic and Health Survey 2011.

	Kathmandu, Nepal: Ministry of Health and Population, New ERA, and ICF International, Calverton, Maryland.
Niger	Institut National de la Statistique (INS) et ICF International, 2013. Enquête Démographique et de Santé et à Indicateurs Multiples du Niger 2012. Calverton, Maryland, USA : INS et ICF International.
Nigeria	National Malaria Elimination Programme (NMEP), National Population Commission (NPopC), National Bureau of Statistics (NBS), and ICF International. 2016. Nigeria Malaria Indicator Survey 2015. Abuja, Nigeria, and Rockville, Maryland, USA: NMEP, NPopC, and ICF International.
Pakistan	Aga Khan University, Pakistan Ministry of Health and UNICEF. Pakistan National Nutrition Survey 2011. 2011. Islamabad: Aga Khan University, MOH, and UNICEF.
Papua New Guinea	National Department of Health, U. P., University of Papua New Guinea, US Centre for Disease Control. (2011). Papua New Guinea National Nutrition Survey, 2005 (PNG NNS 2005)
Peru	Instituto Nacional de Estadística e Informática. Encuesta Demográfica y de Salud Familiar-ENDES 2014. Nacional y Departamental. Lima. Peru. 2015
Rwanda	National Institute of Statistics of Rwanda (NISR) [Rwanda], Ministry of Health (MOH) [Rwanda], and ICF International. 2015. Rwanda Demographic and Health Survey 2014-15. Rockville, Maryland, USA: NISR, MOH, and ICF International.
São Tomé e Príncipe	Instituto Nacional de Estatística (INE) [São Tomé e Príncipe], Ministério da Saúde, e ICF Macro. 2010. *Inquérito Demográfico e Sanitário, São Tomé e Príncipe, IDS STP, 2008-2009*. Calverton, Maryland, USA: INE.
Senegal	Agence Nationale de la Statistique et de la Démographie (ANSD) [Sénégal], et ICF. 2017. Sénégal : Enquête Démographique et de Santé Continue (EDS-Continue 2016). Rockville, Maryland, USA : ANSD et ICF.
Sierra Leone	Statistics Sierra Leone (SSL) and ICF International. 2014. Sierra Leone Demographic and Health Survey 2013. Freetown, Sierra Leone and Rockville, Maryland, USA: SSL and ICF International.
Solomon Islands	Solomon Islands National Statistics Office, Solomon Islands Ministry of Health and Medical Services and the Pacific Community. 2017. *Solomon Islands Demographic and Health Survey, 2015.*
Swaziland	Central Statistical Office (CSO) [Swaziland], and Macro International Inc. 2008. *Swaziland Demographic and Health Survey 2006-07.* Mbabane, Swaziland: Central Statistical Office and Macro International Inc.
Tanzania	Ministry of Health, Community Development, Gender, Elderly and Children (MoHCDGEC) [Tanzania Mainland], Ministry of Health (MoH) [Zanzibar], National Bureau of Statistics (NBS), Office of the Chief Government Statistician (OCGS), and ICF. 2016. Tanzania Demographic and Health Survey and Malaria Indicator Survey (TDHS-MIS) 2015-16. Dar es Salaam, Tanzania, and Rockville, Maryland, USA: MoHCDGEC, MoH, NBS, OCGS, and ICF.
The Gambia	The Gambia Bureau of Statistics (GBOS) and ICF International. 2014. The Gambia Demographic and Health Survey 2013. Banjul, The Gambia, and Rockville, Maryland, USA: GBOS and ICF International.

Timor Leste	General Directorate of Statistics - GDS/Timor-Leste. Ministry of Finance/Timor-Leste and ICF. 2018. Timor-Leste Demographic and Health Survey 2016. Dili, Timor-Leste and Rockville, Maryland, USA: GDS and ICF.
Togo	Ministère de la Planification, du Développement et de l'Aménagement du Territoire (MPDAT), Ministère de la Santé (MS) et ICF International, 2015. Enquête Démographique et de Santé au Togo 2013-2014. Rockville, Maryland, USA : MPDAT, MS et ICF International.
Uganda	Uganda Bureau of Statistics (UBOS) and ICF. 2018. Uganda Demographic and Health Survey 2016. Kampala, Uganda and Rockville, Maryland, USA: UBOS and ICF.
Uzbekistan	Analytical and Information Center, Ministry of Health of the Republic of Uzbekistan [Uzbekistan], State Department of Statistics, Ministry of Macroeconomics and Statistics [Uzbekistan], and ORC Macro. 2004. *Uzbekistan Health Examination Survey 2002.* Calverton, Maryland, USA: Analytical and Information Center, State Department of Statistics, and ORC Macro.
Yemen	Ministry of Public Health and Population (MOPHP), Central Statistical Organization (CSO) [Yemen], Pan Arab Program for Family Health (PAPFAM), and ICF International. 2015. Yemen National Health and Demographic Survey 2013. Rockville, Maryland, USA: MOPHP, CSO, PAPFAM, and ICF International.
Zimbabwe	Zimbabwe National Statistics Agency and ICF International. 2016. *Zimbabwe Demographic and Health Survey 2015: Final Report*. Rockville, Maryland, USA: Zimbabwe National Statistics Agency (ZIMSTAT) and ICF International.

For countries where these data were not available, we derived the overall prevalence of anaemia from WHO estimates,[6] and estimated prevalence of mild/ moderate/ severe subcategories in each age subgroup using observed proportions from the geographically closest country (with similar malaria endemicity) for which DHS data were available. We had to perform this for the following countries:

**Country**	**Source Data Country**
South Africa	Namibia
Zambia	Zimbabwe
Eritrea, Somalia, Djibouti	Ethiopia
Central African Republic	Demographic Republic of Congo
Chad	Niger
Liberia	Cote D'Ivoire
Guinea Bissau	Guinea

Sudan	Egypt
Grenada, Cuba, Dominican Republic, St Lucia	Guyana
Uruguay	Peru
Bhutan	Nepal
China	Cambodia
Tajikistan	Krgyk Republic
Indonesia	Timor Leste

Duration of a case of anaemia was define as three months. After every three month period, individuals were re-modelled and assigned to an anaemia status (non-anaemic, mild/moderate/severe anaemia) in proportion with the prevalence of each anaemia subcategory for the relevant age group and country (see section on 'Modelling of anaemia' below).

Diarrhoea

Incidence of diarrhoea was derived from 2017 Global Burden of Disease estimates for each country.[7] In all countries, these incidence estimates were then assigned as either mild, moderate or severe, in proportions of 64.8%, 34.7% and 0.5% respectively,[8] with durations of 4.3, 6.4 and 8.4 days respectively.[8]

Malaria

Clinical incidence was derived from 2017 GBD data.[7] In all countries, clinical malaria episodes were assigned as 66.3% mild, 33.2% moderate and 0.5% severe in accordance with GBD distributions. Duration of clinical malaria episodes was defined as 14 days, adapted from GBD.[7]

Intervention effect sizes

Summary of effect sizes used in the model

Outcome	Context	Effect size
**Anaemia**	Non-malaria endemic	0.70 [0.61, 0.79]
	Malarial endemic	0.81 [0.73, 0.91]
**Clinical malaria**	Non-malaria endemic<br>OR<br>Malaria endemic with bednet	0.91 [0.84 to 0.97][9]

	Malaria endemic without bednet	1.16 [1.02 to 1.31][9]
**Diarrhoea**	Any	1.09 [0.80, 1.47]

For each cohort, we used an effect size of MNPs on each outcome drawn from a log-normal distribution fitted to the 95% range of estimated effect sizes. Effect sizes (relative risk) for MNPs on the key outcomes of anaemia, diarrhoea and malaria were derived from the same systematic reviews (either published or being updated) that have been used by WHO to define evidence for current global guidelines.

Effects of MNPs on anaemia and diarrhoea were derived from an updated Cochrane review of the effects of MNPs on health outcomes in young children. Briefly, the systematic review included randomised controlled trials of apparently-healthy children aged 6 to 23 months at the start of the intervention, who were randomized to receive MNP formulated with at least iron, zinc and vitamin A, compared with placebo, no intervention, or use of iron-containing supplements. A total of 23 studies were included, and 21 studies (22,162 children) compared the use of MNP versus no intervention or placebo. Primary outcomes of interest were anaemia, iron status and weight. Duration of the intervention was between 2 and 36 months. Most of the included studies were assessed as low risk of selection and attrition bias.

Compared with no intervention or placebo, overall MNP reduced the risk of anaemia by 21% (RR 0.79, 95% CI 0.71-0.88, moderate quality evidence). MNPs effect sizes on anaemia in settings described as malaria-endemic were RR 0.81, 95% CI 0.73-0.91, 11 trials; in non-malaria endemic settings the effect size on anaemia was RR 0.70, 95% CI 0.61-0.79, 1 trial. There was no effect on weight-for-age (Mean Difference -0.01, 95% -0.05-0.02, moderate quality evidence). The effect on diarrhea was not signficant (OR 1.09, 95% CI 0.80-1.47, 3 trials).

Comparison of MNP versus no intervention or placebo on anaemia by malaria endemicity in the study area

Study or Subgroup	MNP Events	MNP Total	No int/placebo Events	No int/placebo Total	Weight	Risk Ratio M-H, Random, 95% CI
**1.6.1 Yes**						
Adu-Afarwuah 2007	18	98	31	96	3.6%	0.57 [0.34, 0.95]
Barffour 2018	274	689	313	726	14.7%	0.92 [0.81, 1.04]
Clarke 2018 (C)	310	538	309	514	15.7%	0.96 [0.87, 1.06]
Giovannini 2006	25	65	43	60	6.3%	0.54 [0.38, 0.76]
Inayati 2012 (C)	15	42	20	53	3.3%	0.95 [0.56, 1.61]
Jack 2012 (C)	219	443	259	457	14.7%	0.87 [0.77, 0.99]
Kounnavong 2011	26	115	14	57	3.0%	0.92 [0.52, 1.62]
Macharia-Mutie 2012	18	93	30	93	3.6%	0.60 [0.36, 1.00]
Menon 2007 (C)	18	76	22	50	3.5%	0.54 [0.32, 0.90]
Sharieff 2006a	5	13	6	13	1.3%	0.83 [0.34, 2.06]
Soofi 2013 (C)	151	210	194	211	16.0%	0.78 [0.71, 0.86]
**Subtotal (95% CI)**		**2382**		**2330**	**85.6%**	**0.81 [0.73, 0.91]**
Total events	1079		1241			
Heterogeneity: Tau² = 0.01; Chi² = 24.69, df = 10 (P = 0.006); I² = 59%						
Test for overall effect: Z = 3.76 (P = 0.0002)						
**1.6.2 No**						
Lundeen 2010 (C)	148	283	206	274	14.4%	0.70 [0.61, 0.79]
**Subtotal (95% CI)**		**283**		**274**	**14.4%**	**0.70 [0.61, 0.79]**
Total events	148		206			
Heterogeneity: Not applicable						
Test for overall effect: Z = 5.45 (P < 0.00001)						
**Total (95% CI)**		**2665**		**2604**	**100.0%**	**0.79 [0.71, 0.88]**
Total events	1227		1447			
Heterogeneity: Tau² = 0.02; Chi² = 32.82, df = 11 (P = 0.0006); I² = 66%						
Test for overall effect: Z = 4.32 (P < 0.0001)						
Test for subgroup differences: Chi² = 3.20, df = 1 (P = 0.07), I² = 68.8%						

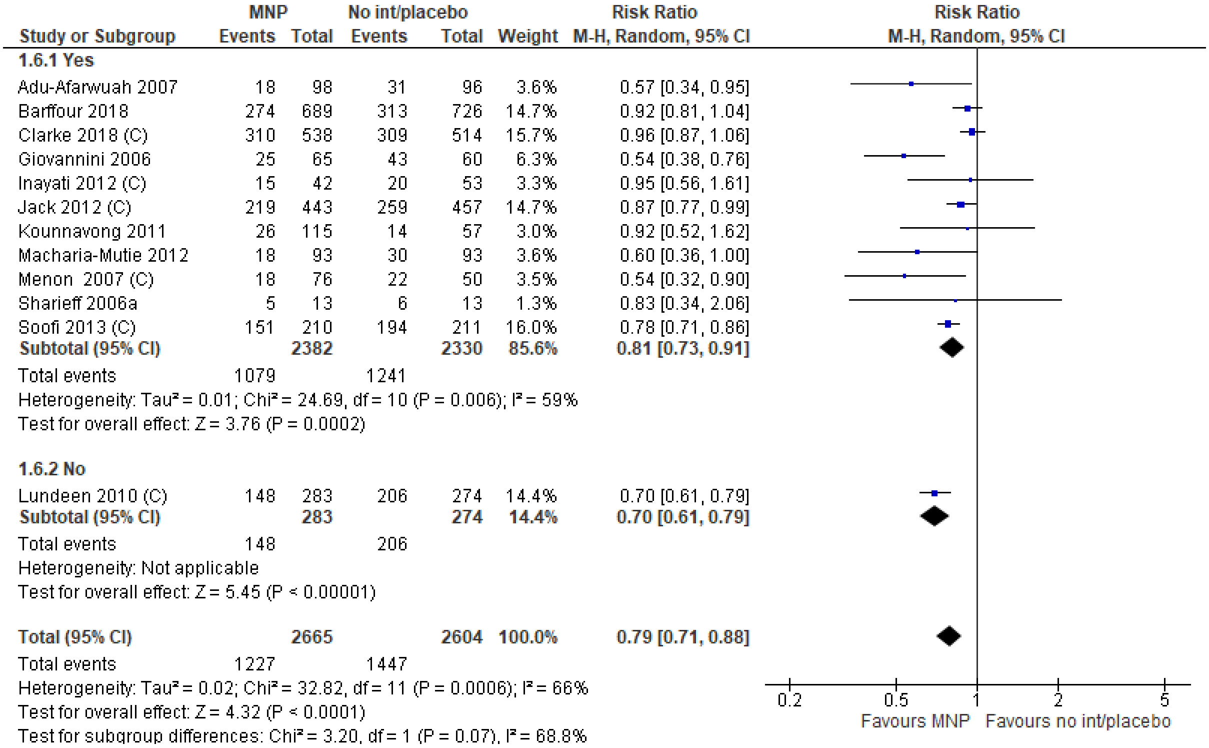

Comparison of MNP versus no intervention or placebo on diarrhoea

Study or Subgroup	log[Odds Ratio]	SE	MNP Total	No int/placebo Total	Weight	Odds Ratio IV, Random, 95% CI
Sharieff 2006a	-0.665	0.706	24	25	4.4%	0.51 [0.13, 2.05]
Barffour 2018	-0.0408	0.0867	841	847	46.9%	0.96 [0.81, 1.14]
Soofi 2013 (C)	0.27	0.0754	746	779	48.7%	1.31 [1.13, 1.52]
**Total (95% CI)**			**1611**	**1651**	**100.0%**	**1.09 [0.80, 1.47]**
Heterogeneity: Tau² = 0.04; Chi² = 8.60, df = 2 (P = 0.01); I² = 77%						
Test for overall effect: Z = 0.54 (P = 0.59)						

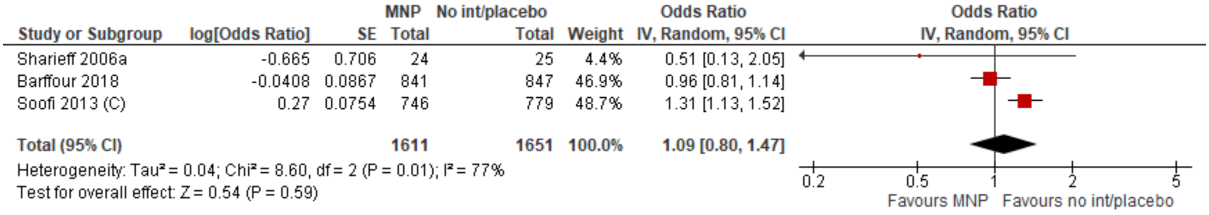

Given the paucity of data on MNPs and malaria from MNP review, effects of MNPs on clinical malaria incidence were derived from the key 2016 Cochrane review, which reported that the effect of iron interventions (both iron supplements and MNPs) on malaria risk was modified by co-provision of malaria prevention measures.[9] WHO guidelines for provision of MNPs in malaria endemic countries are derived from this Cochrane review. For our analysis, we defined malaria prevention as provided when a child slept under a bednet. Children were assigned a probability to sleeping under a bednet according to national data (based on UNICEF and World Malaria reports).[10,11] Sleeping under a bednet is also known to reduce malaria incidence by ~50% (based on a Cochrane review); thus, the model accounted for this by also reducing the baseline incidence of malaria in such children by 50%.[12]

Effects on cognition

Although iron interventions have been considered able to benefit long-term cognitive development, and to improve educational performance and earning potential, we identified only two trials[13,14] which had compared iron supplementation with placebo to children <2 years of age and then undertaken long term follow up. Neither trial used MNPs. In both trials, no effect on cognitive performance by mid-primary school age was seen (meta-analysis of trials: standardised mean difference 0.00 [-0.15, 0.16]). Systematic reviews of RCTs of iron supplementation and MNPs in young children have not confirmed short term benefits on child cognitive development.[15,16] Thus, we could not incorporate an effect from MNPs on long term cognition or future earning potential in the model.

Outcomes

Disability weights for individual diseases, for each severity level, were derived from GBD 2013.[17]

We estimated country-specific case fatality rates for each of anaemia, diarrhoea and malaria, using data from GBD: in children aged 1-4 years (data in children 6-12 months were not available) country-specific estimates of the number of deaths for each condition were divided by the country-specific total number of cases (data shown in Supplementary Table 1). The number of deaths were fixed based on the overall incidence of each infection and the country specific case-specific mortality rate for that disease. DALYs were calculated as the sum of years of life lost (YLLs) and years lived with disability (YLDs). YLLs were equal to the time remaining between mortality and that country's life expectancy. Years lived with disability (YLDs) were calculated by multiplying the duration of each adverse event with the corresponding disability weight.

Note that we used disability weights for mild, moderate and severe anaemia based on commonly utilised haemoglobin thresholds used for designating severity: Mild anaemia: Hb 10-10.9g/dL, Moderate anaemia: 7-9.9 g/dL, Severe anaemia <7g/dL. However, the GBD uses clinical descriptors of anaemia which may not be directly reflected by these threshods:

Mild Anaemia: feels slightly tired and weak at times, but this does not interfere with normal daily activities.

Moderate Anaemia: feels moderate fatigue, weakness, and shortness of breath after exercise, making daily activities more difficult.

Severe Anaemia: feels very weak, tired and short of breath, and has problems with activities that require physical effort or deep concentration.[17]

Modelling

Simulated individuals entered the model at birth and progressed through life in two-week cycles until age 18 months. During each cycle, a child could incur anaemia, malaria, and/or diarrhoea according to their country-specific, age-specific incidences. Given that multiple adverse events could occur simultaneously in a given patient within the same two-week time interval, the YLD for an individual in interval t, L_t, was calculated as:

$$L_t = 1 - \prod_d (1 - W_d \, e_{d,t})$$

where $e_{d,t}$ is a binary variable that indicates whether the individual experienced disease d in cycle t, and W_d is the disability weight associated with disease d over the assumed duration of that disease.

As an example, this logic implies that if an individual experienced disease 1 and 3, but not disease 2 (in a model with $d = 3$ diseases), and the diseases incurred DALYs of 0.1 0.2 and 0.3, we would get an overall DALY loss of:

$$L = 1 - (1 - 0.1 \times 1)(1 - 0.2 \times 0)(1 - 0.3 \times 1),$$
$$L = 1 - 0.9 \cdot 1 \cdot 0.7,$$
$$L = 1 - 0.63 = 0.37.$$

If they only had disease 1, they would have lost 0.1. If they only had disease 3, they would have lost 0.3. If they had both, the loss would be less than the sum (0.4) because disease 3 can't take away life-years already lost to disease 1.

Modelling of anaemia

We considered that during intervention, MNPs could both prevent and cure anaemia. If a child entered the intervention period (at six months) anemic, they could be cured after four

weeks of intervention (two cycles) at a probability derived from the relative risk of anaemia between the control and intervention arms. If cured, they thereafter experienced an ameliorated risk of becoming anemic (prevention). However, anemic children in the MNP arm who did not recover upon intervention were deemed 'non-responsive to iron' and remained anemic for the full three months, and were thereafter subject to the country-age-specific probability of becoming anaemic / non anaemic again, albeit with a reduced risk because they were in an MNP arm. *Preventive* effects of MNPs (i.e. through replenished iron stores) on anaemia were considered to be sustained for six months beyond the treatment course (i.e. from 12-18 months of age), based on expected improvement in iron stores and trial data indicating similar haemoglobin concentrations at six months post-intervention between 6-36 month old children whom had previously received iron and placebo[18]. The *curative* effects of iron on anaemia were considered to occur only *during* intervention, i.e. between 6 and 12 months of age. Effects of MNPs on adverse events (malaria, diarrhoea) were restricted to the duration of the intervention (i.e. from 6 to 12 months of age) based on trials indicating no evidence of harmful effect during the post-intervention period.[19,20]

For each country and intervention arm, statistics were based on 5,000 simulations of 5,000 individuals for each of the intervention groups, and also for the control group. The mean difference between simulations for each group is reported. All programming and analyses were conducted in Matlab 9.6 (Mathworks, Natick, MA, USA).

Associations with MNP effects on disease burden

We screened 2 x 2 associations between net benefit achieved by MNPs (as defined by DALYs, YLDs and YLLs), and baseline disease epidemiology (mild/ moderate/ severe anaemia, malaria and diarrhoea) at the country level. For each analysis, we calculated Spearman's Rho for the overall set of countries, and disaggregated by region.

Model validation

Outputs for countries where recent RCTs reported on efficacy of MNPs were compared to the simulation results. We reviewed relative risks of anaemia reported in the trials to those produced by the model in the same country.

Uncertainty

Uncertainty intervals were calculated to show the 2.5 and 97.5 percentiles of the mean differences between the treatment and control arms, over 5,000 simulated comparisons. They are indicated in Figure 3 and detailed in individual country reports shown in Table S3. These intervals reflect parameter or “second-order” uncertainty, resulting from uncertainty in the estimation of model parameters, principally uncertainty around the relative rates of each disease in the treatment, versus the control arm. We incorporate this uncertainty by sampling parameter values from their respective distributions in each of the 5,000 simulations per country. We minimised stochastic or “first-order” uncertainty, resulting from the fact that patients facing the same probabilities may experience different outcomes, through a sufficiently high number of simulated children (5,000) per model run.

Costs

All costs are reported in 2015 US$. We considered direct healthcare costs comprising the cost of delivering each intervention and the direct cost of treating health events (malaria, diarrhoea and respiratory infections), but not indirect healthcare costs. Cost of interventions was derived from the lowest estimated price by UNICEF. Thus, MNPs were costed at US$0.03 per dose.[21] We estimated the non-drug costs of providing the programme as US$4.50 per child, using experience-based estimates of 2015 US$ 4-5 per year from the Home-Fortification Technical Advisory Group[22].

To model possible effects of regional variation in programme costs, sensitivity analysis, we adopted the World Bank regional cost multipliers.[23] These apply a cost multiplier for programme costs of 1.0 for Africa, South Asia and East Asia and the Pacific; 2.35 for Latin America; 2.20 for the Middle East and North Africa; and 1.35 for Europe and Central Asia.

Healthcare costs for adverse events were calculated as the product of either outpatient consultations or length of inpatient stay (days), as reported for each health event, and their unit costs. It was assumed that severe malaria, severe pneumonia and severe diarrhoea would require hospitalizations amounting to 4.3 days[24], 5 days[25], and 8.4 days[8], respectively. Moderate cases would require one outpatient clinic visit, while mild cases (the majority) would not receive any health care; thus, the majority of infection episodes (which were mild) do not incur health care cost. Unit costs for inpatient days and outpatient visits were taken from the WHO-CHOICE database[26]. First, 2007-2008 estimates were converted in local

currency, then were inflated to their 2015 values using country-specific inflation rates. Finally, these local currency values were converted to US$ using the average exchange rate for 2015. No discounting was applied to either costs or outcomes (DALYs) given the one year modelling horizon.

Cost Effective Acceptability Curves (CEACs)

Each of the 5,000 simulations produced a different estimate of the DALYs averted as a consequence of the intervention, and a different estimate of the total additional healthcare costs that were incurred. These values varied as a consequence of demographic stochasticity, and on different modelled relative rates from the effect size distributions, creating a probability distribution of incremental cost-effectiveness ratios (cost per DALY averted). We used these probability distributions to create Cost Effective Acceptability Curves (CEACs), a measure of the probability that an intervention will be cost-effective at a given threshold. These probabilities are estimated using the proportion of simulations where the cost per DALY averted falls below a particular cost effectiveness threshold value.

Sensitivity analysis

We conducted sensitivity analysis on coverage to the intervention, from 100% (base case) to 75%, 50% and 25%. Drug procurement and programme costs remained identical as per 100% coverage, but in the lesser coverage scenarios, non-covered individuals in the treatment arm did not receive the intervention and hence did not experience either possible associated benefits or harms.

Secondly, we varied costs of programme delivery according to published regional multipliers for nutrition programmes used by the World Bank, and recalculated cost per DALY averted.[23]

Supplementary Data

Table S1 Case-specific mortality for diarrhoea and malaria for each country included in the analysis, derived from Global Burden of Disease data.

Region	Country	Diarrhoea	Malaria
Africa	Kenya	0.00034099	0.00085418
Africa	South Africa	0.00022218	0.0045771
Africa	Namibia	0.00031108	0.00092824
Africa	Botswana	0.00021279	0.00061868
Africa	Zimbabawe	0.00039224	0.001876
Africa	Zambia	0.00028931	0.0022156
Africa	Angola	0.00023363	0.0050997
Africa	Gabon	4.31E-05	0.001462
Africa	Uganda	0.00025864	0.0023195
Africa	Ethiopia	0.0003373	0.0025571
Africa	Eritrea	0.00081559	0.0036619
Africa	Madagascar	0.00048228	0.0023116
Africa	Djibouti	0.00037283	0.00088919
Africa	Mozambique	0.0002835	0.0025399
Africa	Tanzania	0.00016296	0.002331
Africa	Democratic F	0.00017116	0.0038145
Africa	Central Afric	0.00034366	0.0043664
Africa	Cameroon	0.00037443	0.0051662
Africa	Chad	0.001409	0.0077952
Africa	Nigeria	0.001269	0.0050277
Africa	Niger	0.0016009	0.0059911
Africa	Mali	0.00067225	0.0070178
Africa	Burkina Faso	0.00064496	0.006062
Africa	Congo	9.85E-05	0.0025374
Africa	Somalia	0.0014695	0.0056868
Africa	Ghana	0.00012653	0.0020757
Africa	Togo	0.00031992	0.0031511
Africa	Benin	0.0004737	0.0035659
Africa	Cote d'Ivoire	0.00025135	0.0036989
Africa	Liberia	0.00024871	0.0020295
Africa	Sierra Leone	0.0004715	0.0050691
Africa	Guinea	0.00035477	0.0046977
Africa	Senegal	0.0005162	0.0038964
Africa	Gambia	0.00030622	0.0023279
Africa	Cape Verde	3.73E-05	0
Africa	Guinea-Bissa	0.0013717	0.008882
Africa	Mauritania	0.00033656	0.0035689
Africa	Rwanda	0.0003262	0.0043868
Africa	Burundi	0.00052544	0.0034667
Africa	Sao Tome an	0.00013779	0.00011193
Africa	Lesotho	0.00055009	0
Africa	Malawi	0.00048981	0.0034019
Africa	Swaziland	0.00037533	0.00059932
Africa	Equatorial Gu	6.56E-05	0.0038287
Africa	Egypt	5.58E-05	0
Africa	Sudan	0.00021661	0.00249
Asia	Yemen	6.97E-05	0.00012037
Asia	Kyrgyzstan	8.98E-05	0
Asia	Tajikistan	0.000172	0
Asia	Indonesia	7.46E-05	0.00015575
Asia	Mongolia	4.69E-06	0
Asia	China	2.12E-05	0.0010566
Asia	Philippines	0.00014124	1.42E-05
Asia	Myanmar	0.00013138	0.00024511
Asia	Solomon Isla	9.04E-05	0.00034959
Asia	Timor Leste	0.00010095	5.84E-05
Asia	India	0.00020732	0.00035372
Asia	Pakistan	0.00018554	0.00067021
Asia	Afghanistan	0.00026558	0.0023148
Asia	Nepal	5.50E-05	0.00067494
Asia	Bangladesh	5.71E-05	1.17E-05
Asia	Bhutan	2.73E-05	0.19688
Asia	Lao PDR	0.00024466	0.00030279
Asia	Cambodia	4.76E-05	0.0006589
Asia	Uzbekistan	3.12E-05	0
Asia	Saudi Arabia	1.36E-05	0.0038618
Asia	Papua New (	0.00021795	0.00027505
Latin Americ	Grenada	2.16E-05	0
Latin Americ	Colombia	3.32E-05	0.00010687
Latin Americ	Cuba	8.42E-06	0
Latin Americ	Dominican R	4.50E-05	4.56E-05
Latin Americ	Peru	2.59E-05	8.94E-05
Latin Americ	Uruguay	0.00015311	0
Latin Americ	Ecuador	4.91E-05	0.0020945
Latin Americ	Bolivia	8.26E-05	0.00021708
Latin Americ	Haiti	0.00027187	0.00071814
Latin Americ	Guyana	7.61E-05	0.00020385
Latin Americ	St. Lucia	2.73E-05	0
Latin Americ	Guatemala	0.00022462	3.35E-05

Table S2 Effect of MNP on DALYs, YLDs and YLLs in each country modelled, at 100%, 75%, 50% and 25% coverage.

	100% coverage							75% coverage				50% coverage				25% coverage			
Country	DALY averted/ 10000 children	[95% CI]	YLL averted/ 10000 children	[95% CI]	YLD averted/ 10000 children	[95% CI]	USD/DALY averted	DALY averted/ 10000 children	YLL averted/ 10000 children	YLD averted/ 10000 children	USD/DALY averted	DALY averted/ 10000 children	YLL averted/ 10000 children	YLD averted/ 10000 children	USD/DALY averted	DALY averted/ 10000 children	YLL averted/ 10000 children	YLD averted/ 10000 children	USD/DALY averted
Global	22.7		-30.9		55.5		3576.4	16.9	-23.5	41.65	4829.6	11.2	-15.65	27.7	7242.3	5.4	-8.0	13.9	14913.3
**Africa**																			
Angola	-45.4	[-207.8, 109.5]	-107.7	[-261.8, 40.2]	62.3	[54.0, 69.2]	N/A	-34.9	-81.6	46.7	N/A	-23.3	-54.4	31.1	N/A	-12.3	-27.8	15.5	N/A
Benin	24.5	[-144.4, 183.7]	-40.1	[-202.2, 112.6]	64.5	[57.8, 71.1]	4092.3	17.6	-30.8	48.4	5687.3	11.8	-20.5	32.3	8463.5	5.3	-10.8	16.1	18667
Botswana	32.8	[-27.5, 79.2]	-14.0	[-68.1, 27.0]	46.8	[40.6, 52.1]	3257.5	24.6	-10.6	35.1	4266.8	16.3	-7.1	23.4	6307.8	8.1	-3.6	11.7	12528.8
Burkina Faso	30.7	[-266.7, 334]	-81.8	[-368.6, 212.2]	112.5	[101.9, 121.8]	3267	21.7	-62.7	84.4	4598.8	14.5	-41.8	56.2	6888.6	6.3	-21.7	28	15830
Burundi	-40.1	[-231.0, 135.0]	-83.0	[-266.9, 85.7]	43.0	[35.9, 49.3]	N/A	-30.8	-63	32.2	N/A	-20.5	-42	21.5	N/A	-10.7	-21.5	10.8	N/A
Cameroon	-56.5	[-297.2, 161.0]	-113.0	[-344.7, 96.0]	56.5	[47.5, 65.0]	N/A	-43.3	-85.8	42.4	N/A	-28.9	-57.2	28.3	N/A	-15.1	-29.3	14.2	N/A
Cape Verde	41	[6.0, 69.1]	-6.7	[-30.7, 11.8]	47.7	[36.7, 57.3]	2632	30.8	-5	35.8	3437.1	20.5	-3.4	23.8	5065.9	10.3	-1.7	11.9	9858.6
Central African Republic	-113.2	[-368.9, 123.3]	-171.5	[-417.1, 56.4]	58.2	[48.2, 66.9]	N/A	-85.8	-129.4	43.6	N/A	-57.2	-86.3	29.1	N/A	-29	-43.7	14.7	N/A
Chad	-220.3	[-992.0, 388.0]	-298.5	[-1058.8, 299.8	78.2	[66.8, 88.2]	N/A	-166.8	-225.4	58.6	N/A	-111.3	-150.3	39	N/A	-56.6	-76.2	19.6	N/A
Congo	-33.5	[-153.2, 87.0]	-92.6	[-203.8, 20.3]	59.1	[50.6, 66.8]	N/A	-25.7	-70	44.3	N/A	-17.1	-46.6	29.5	N/A	-8.9	-23.7	14.7	N/A
Cote d'Ivoire	-7.2	[-140.9, 122.5]	-77.7	[-201.0, 43.7]	70.4	[60.1, 78.8]	N/A	-6.1	-58.9	52.8	N/A	-4	-39.3	35.3	N/A	-2.4	-20.1	17.7	N/A
Democratic Republic of Congo	-23.2	[-191.0, 141.3]	-83.2	[-241.6, 73.5]	60.0	[50.6, 67.8]	N/A	-18.2	-63.2	44.9	N/A	-12.1	-42.1	30	N/A	-6.6	-21.6	15	N/A
Djibouti	28.2	[-55.0, 101.4]	-21.0	[-98.1, 46.2	49.2	[43.1, 55.2]	3576.4	20.9	-16	36.9	4799.6	13.9	-10.7	24.5	7206	6.8	-5.5	12.3	14591.9
Egypt	18.5	[-11.6, 42.1]	-6.2	[-29.4, 11.5]	24.7	[17.8, 30.6]	5652.5	13.8	-4.7	18.5	7442.2	9.3	-3.1	12.4	10954.7	4.6	-1.6	6.2	21667.2
Equatorial Guinea	-106.7	[-320.6, 97.2]	-179.6	[-385.2, 15.2]	72.9	[64.6, 82.0]	N/A	-80.7	-135.4	54.7	N/A	-53.9	-90.3	36.4	N/A	-27.4	-45.6	18.2	N/A
Eritrea	4.5	[-250.6, 222.8]	-67.7	[-315.3, 143.1]	72.2	[64.7, 79.7]	22195.6	2.6	-51.6	54.2	38251.3	1.7	-34.4	36.1	57619.6	0.4	-17.7	18	274174.8
Ethiopia	29.6	[-67.2, 116.6]	-22.3	[-111.5, 57.7]	51.8	[44.3, 58.9]	3371	21.5	-17.3	38.8	4626.7	14.5	-11.5	26	6872.2	6.8	-6.1	13	14526.6
Gabon	18.2	[-52.1, 83.6]	-37.2	[-99.6, 20.6]	55.4	[47.5, 63.0]	6565.4	13.3	-28.2	41.5	8622.2	8.9	-18.8	27.7	12246.1	4.2	-9.6	13.8	25199.6
Gambia	44.5	[-77.1, 149.2]	-34.9	[-148.6, 61.7]	79.4	[71.5, 87.5]	2240	32.9	-26.6	59.6	3024.5	21.9	-17.8	39.7	4540.5	10.6	-9.2	19.8	9402.3
Ghana	25.4	[-61.9, 109.0]	-45.6	[-124.4, 30.1]	70.9	[62.6, 78.9]	3945.1	18.7	-34.6	53.3	5342.6	12.4	-23.1	35.4	8038.7	5.8	-11.8	17.7	16968.7
Guinea	-150	[-451.0, 125.9]	-235.6	[-526.8, 30.7]	85.6	[75.8, 95.3]	N/A	-113.4	-177.7	64.3	N/A	-75.7	-118.4	42.8	N/A	-38.5	-59.8	21.4	N/A
Guinea-Bissau	-28.9	[-407.9, 289.3]	-118.2	[-488.8, 191.5]	89.3	[80.9, 97.8]	N/A	-23.7	-90.6	66.9	N/A	-15.8	-60.4	44.7	N/A	-9.2	-31.5	22.3	N/A
Kenya	26.2	[-76.5, 116.6]	-29.7	[-124.8, 53.4]	55.8	[48.4, 63.2]	3848.8	19.4	-22.5	41.9	5163.2	12.9	-15	27.9	7768.1	6.2	-7.7	13.9	15915.9
Lesotho	6.4	[-111.7, 115.1]	-37.2	[-149.1, 64.6]	43.6	[37.4, 50.6]	15678.9	4.7	-28	32.7	21351.1	3.2	-18.7	21.8	31464.4	1.5	-9.4	10.9	64793.5
Liberia	-43.7	[-219.8, 95.1]	-83.5	[-248.6, 46.3]	39.8	[28.9, 48.7]	N/A	-33.3	-63.1	29.8	N/A	-22.2	-42	19.9	N/A	-11.4	-21.3	9.9	N/A
Madagascar	-14.1	[-229.4, 181.1]	-69.6	[-277.1, 117.7]	55.5	[47.7, 63.4]	N/A	-11.2	-52.8	41.6	N/A	-7.5	-35.2	27.7	N/A	-4.1	-18	13.9	N/A
Malawi	-9.4	[-269.7, 212.3]	-77.9	[-327.3, 133.7]	68.4	[57.6, 78.6]	N/A	-7.8	-59.1	51.3	N/A	-5.3	-39.4	34.1	N/A	-3.1	-20.2	17.1	N/A
Mali	-184	[-545.0, 140.8]	-272.7	[-625.4, 44.0]	88.7	[80.4, 96.8]	N/A	-139.4	-206	66.6	N/A	-93	-137.3	44.3	N/A	-47.5	-69.6	22.1	N/A
Mauritania	40	[-87.3, 152.7]	-48.5	[-166.9, 56.9]	88.4	[79.6, 95.8]	2530.9	29.2	-37.1	66.4	3442.6	19.3	-24.8	44.1	5174.5	9.2	-12.9	22.1	10805.3
Mozambique	-13.8	[-134.3, 112.8]	-91.3	[-204.7, 27.4]	77.5	[70.3, 85.4]	N/A	-10.7	-69	58.3	N/A	-7.1	-46	38.9	N/A	-4	-23.3	19.3	N/A
Namibia	23.3	[-95.4, 125.9]	-33.7	[-145.6, 62.0]	57.0	[50.2, 63.8]	4531.4	17.3	-25.5	42.8	6012.9	11.5	-17	28.5	8881	5.7	-8.6	14.3	17731.6
Niger	-359.6	[-999.2, 229.1]	-439.2	[-1070.8, 141.3	79.6	[71.6, 87.8]	N/A	-271.2	-330.9	59.7	N/A	-180.9	-220.6	39.7	N/A	-91.5	-111.3	19.8	N/A
Nigeria	-165	[-546, 141.7]	-243.1	[-616.3, 56.3]	78.1	[70.2, 85.4]	N/A	-124.8	-183.4	58.6	N/A	-83.2	-122.3	39.1	N/A	-42.3	-61.9	19.6	N/A
Rwanda	8.3	[-106.3, 109.1]	-32.1	[-140.1, 62.2]	40.3	[33.9, 47.0]	12035.8	5.2	-25	30.2	19009.9	3.5	-16.7	20.2	28640.4	1.1	-9	10.1	90002.7
Sao Tome and Principe	40.2	[-18.2, 87.0]	-14.9	[-66.0, 25.3]	55.1	[47.8, 61.6]	2464.2	30	-11.2	41.3	3296	20.1	-7.5	27.6	4925.6	10	-3.8	13.8	9921.6
Senegal	2.7	[-287.9, 218.7]	-76.7	[-358.2, 131.0]	79.4	[70.3, 87.7]	37816.5	1.2	-58.5	59.6	85349.2	0.7	-39	39.7	140223	-0.3	-20.1	19.8	N/A
Sierra Leone	-87.9	[-324.4, 146.8]	-160.0	[-387.8, 66.4]	72.1	[63.4, 80.3]	N/A	-66.9	-120.9	54	N/A	-44.6	-80.6	36.1	N/A	-22.9	-40.9	18	N/A
Somalia	-40.8	[-401.8, 258.9]	-111.0	[-464.7, 182.1]	70.3	[63.0, 76.8]	N/A	-31.9	-84.5	52.6	N/A	-21.2	-56.3	35.2	N/A	-11.5	-29	17.5	N/A
South Africa	40.9	[23.7, 55.4]	-3.0	[-15.3,6.6]	43.9	[39.0, 48.9]	2452.3	30.6	-2.4	32.9	3268.8	20.3	-1.6	21.9	4916.6	10.1	-0.9	11	9832.2
Sudan	24.3	[-114.7, 137.3]	-46.2	[-173.3, 56.2]	70.5	[58.6, 81.1]	4403.1	17.7	-35.2	52.8	5934.5	11.8	-23.4	35.3	8703	5.5	-12.1	17.6	18207.8
Swaziland	12.5	[-109.2, 103.4]	-32.5	[-147.2, 51.8]	45.0	[38.0, 51.6]	8225.1	9.2	-24.5	33.7	11017.2	6.2	-16.3	22.5	16261.2	3	-8.2	11.2	33110
Tanzania	40.6	[-25.7, 100.8]	-15.5	[-75.1, 38.0]	56.1	[49.4, 62.9]	2462.4	30	-12.1	42.1	3320.5	19.9	-8.1	28	4989.6	9.7	-4.4	14.1	10226.9
Togo	-83.7	[-296, 122.9]	-155.0	[-358.4, 43.9]	71.3	[62.4, 79.0]	N/A	-63.4	-116.9	53.5	N/A	-42.3	-77.9	35.6	N/A	-21.6	-39.4	17.8	N/A
Uganda	28	[-92.0, 136.8]	-33.2	[-144.9, 68.7]	61.2	[52.9, 68.1]	3570.5	20.5	-25.4	45.9	4859.5	13.7	-16.9	30.6	7278.6	6.5	-8.8	15.3	15221.5
Zambia	-9	[-145.5, 113.1]	-50.5	[-179.5, 64.4]	41.5	[34.1, 48.7]	N/A	-7.2	-38.4	31.1	N/A	-4.8	-25.6	20.8	N/A	-2.6	-13.1	10.4	N/A
Zimbabwe	0.2	[-147, 120.6]	-41.0	[-181.3, 72.2]	41.3	[34.3, 48.5]	425696.9	-0.3	-31.2	30.9	N/A	-0.2	-20.8	20.6	N/A	-0.4	-10.7	10.3	N/A
Median	1.45		-68.7		60.6		3897.0	0.45	-52.2	45.4	5163.2	0.25	-34.8	30.3	7768.1	-0.35	-17.85	15.15	15873.0
**Asia and the Middle East**																			
Afghanistan	-12.1	[-186.9, 139.4]	-44.4	[-207.9, 97.0]	32.3	[20.9, 42.4]	N/A	-9.5	-33.7	24.2	N/A	-6.4	-22.5	16.1	N/A	-3.5	-11.5	8.1	N/A
Bangladesh	63.9	[50.1, 76.0]	-2.5	[-10.8, 4.1]	66.4	[60.9, 71.9]	1557.1	48	-1.9	49.9	2070.1	31.8	-1.2	33.1	3116.7	16	-0.6	16.7	6182.6
Bhutan	32.3	[-12.3, 76.0]	-2.5	[-41.1, 35.5]	34.8	[28.9, 40.4]	3135.9	22.6	-3.6	26.1	4458.7	14.3	-3.1	17.4	6989.6	6.1	-2.6	8.7	16333.8
Cambodia	58	[36.9, 76.5]	-4.0	[-18.6, 8.5]	62.0	[55.5, 68.1]	1729.5	43.3	-3.1	46.4	2307.1	28.9	-2.1	31	3447.1	14.4	-1.1	15.6	6877.6
China	25.5	[16.2, 33.3]	-1.1	[-5.7, 2.5]	26.6	[21.9, 30.7]	4019.9	19	-0.8	19.9	5336.3	12.8	-0.6	13.3	7882.7	6.4	-0.3	6.7	15622.4
India	54.4	[20.3, 85.0]	-8.3	[-36.9, 16.6]	62.6	[57.2, 68.4]	1839.6	40.6	-6.3	46.9	2453.5	27.2	-4.2	31.4	3662.7	13.5	-2.2	15.7	7347.2
Indonesia	18.7	[-15.7, 49.5]	-8.4	[-35.6, 15.7]	27.1	[20.0, 33.7]	5638.2	13.9	-6.4	20.2	7474.4	9.2	-4.2	13.5	11086.5	4.6	-2.1	6.8	21821.5
Kyrgyzstan	15.4	[-98.9, 114.7]	-23.7	[-120.7, 61.2]	39.2	[21.8, 53.5]	6673.5	11.6	-17.8	29.4	8820.7	7.7	-11.9	19.5	13166.4	3.8	-6	9.8	26207.6
Lao PDR	42.2	[-31.1, 100.3]	-18.1	[-84.9, 34.4]	60.3	[53.7, 65.9]	2388.6	31.6	-13.7	45.2	3178.3	20.9	-9.1	30	4779.5	10.4	-4.6	15.1	9535.1
Mongolia	18.7	[12.9, 24.0]	-0.3	[-1.4, 0.6]	18.9	[14.3, 23.4]	5423.3	14	-0.2	14.2	7190.3	9.3	-0.1	9.5	10705.5	4.7	-0.1	4.7	21296.1
Myanmar	40.5	[-2.9, 77.6]	-10.9	[-47.7, 19.9]	51.4	[44.8, 57.7]	2474.4	30.3	-8.2	38.6	3291.4	20.2	-5.5	25.6	4939.5	10	-2.8	12.8	9883
Nepal	33.2	[17.6, 47.4]	-2.8	[-12.9, 6.1]	36.0	[30.5, 41.3]	2998.1	24.8	-2.2	27	4003	16.5	-1.4	18	6003.2	8.2	-0.7	8.9	12081.9
Pakistan	42.2	[-21.9, 99.6]	-15.1	[-71.2, 34.6]	57.3	[49.3, 65.1]	2387.5	31.5	-11.5	43	3185.6	21	-7.7	28.7	4746.5	10.3	-3.9	14.3	9626.7
Papua New Guinea	27.3	[-73.6, 115.1]	-28.2	[-120.0, 51.0]	55.5	[46.3, 64.1]	3779.1	20.5	-21.2	41.7	4988.1	13.6	-14.1	27.7	7456	6.7	-7.1	13.9	14913.3
Philippines	22	[-15.4, 53.5]	-9.1	[-41.4, 17.8]	31.1	[26.0, 35.8]	4630	16.5	-6.9	23.4	6131.3	10.9	-4.6	15.5	9220.4	5.5	-2.3	7.8	18090.5
Solomon Islands	26.2	[-26.6, 67.6]	-15.5	[-59.8, 18.2]	41.6	[33.2, 49.3]	3985.5	19.5	-11.7	31.2	5278.1	13	-7.8	20.8	7839.6	6.4	-4	10.4	15605.7
Tajikistan	24.1	[-25.6, 64.7]	-11.4	[-54.9, 23.4]	35.4	[29.4, 41.3]	4145.7	18	-8.6	26.6	5531.2	12	-5.7	17.7	8268.7	6	-2.9	8.9	16589.3
Timor Leste	27.1	[-11.9, 60.3]	-9.6	[-42.1, 17.4]	36.7	[30.1, 42.9]	3701	20.3	-7.2	27.5	4930.2	13.5	-4.8	18.3	7370.3	6.8	-2.4	9.2	14590.6
Uzbekistan	43.6	[34.7, 51]	-0.9	[-5.0, 2.3]	44.5	[39.8, 48.7]	2283.2	32.7	-0.7	33.4	3040.7	21.8	-0.5	22.2	4557.4	10.9	-0.2	11.1	9099.1
Yemen	105.2	[39.9, 160.2]	-13.0	[-64.7, 30.1]	118.2	[104.6, 130.1]	1041.2	79	-9.8	88.8	1353	52.5	-6.5	59.1	1979.9	26.2	-3.3	29.5	3888.9
Median	29.8		-9.35		40.4		3135.9	21.55	-7.05	30.3	4458.7	13.95	-4.7	20.15	6989.6	6.75	-2.5	10.1	14590.6
**Latin America**																			
Bolivia	74.3	[33.5, 108.7]	-9.3	[-40.1, 16.9]	83.6	[73.6, 91.8]	1398.3	55.7	-7	62.7	1842.3	37.1	-4.7	41.8	2730.9	18.5	-2.4	20.9	5415.2
Colombia	18.1	[1.4, 32.4]	-3.1	[-14.5, 5.8]	21.2	[15.9, 26.6]	6026.4	13.6	-2.3	15.9	7849.2	9	-1.6	10.6	11519.9	4.5	-0.8	5.3	22525.1
Cuba	30.2	[24.3, 35.3]	-0.4	[-1.8, 0.8]	30.5	[26.2, 34.5]	N/A	22.7	-0.3	22.9	N/A	15.1	-0.2	15.3	N/A	7.5	-0.1	7.6	NaN
Dominican Republic	24.7	[-2.2, 46.0]	-5.5	[-24.9, 9.6]	30.2	[22.6, 36.4]	4381.4	18.5	-4.1	22.7	5708.8	12.4	-2.8	15.2	8338.2	6.1	-1.4	7.5	16541.8
Ecuador	20.1	[-0.3, 37.9]	-4.1	[-19.1, 8.4]	24.3	[18.8, 29.5]	4917.6	15	-3.2	18.2	6607.4	10	-2.2	12.1	9947.5	4.9	-1.2	6.1	20042.7
Grenada	37.3	[25.5, 48.6]	-1.5	[-7.5, 3.9]	38.9	[33.0, 44.7]	2880.1	28	-1.2	29.2	3768.9	18.8	-0.8	19.5	5515	9.3	-0.4	9.7	10780.5
Guatemala	-4.2	[-149.6, 102.8]	-36.1	[-172.0, 63.2]	32.0	[22.3, 39.7]	N/A	-3.2	-27.2	24	N/A	-2.1	-18.1	16	N/A	-1.1	-9.1	8	N/A
Guyana	41.4	[19.8, 58.3]	-4.4	[-20.5, 8.1]	45.8	[40.3, 50.1]	2390.2	31	-3.4	34.4	3189.9	20.7	-2.2	23	4779.4	10.3	-1.2	11.4	9630.8
Haiti	31.9	[-73.0, 113.6]	-26.5	[-123.7, 47.9]	58.4	[50.6, 65.7]	3150.1	23.7	-20.1	43.8	4217.9	15.9	-13.4	29.3	6276.8	7.9	-6.8	14.7	12632
Peru	39.6	[22.9, 54.1]	-2.7	[-12.7, 5.3]	42.3	[35.6, 48.8]	2689.2	29.7	-2	31.8	3516.9	19.8	-1.4	21.2	5185.9	9.9	-0.7	10.6	10165.2
St. Lucia	33.9	[20.6, 46]	-2.7	[-10.6, 4.1]	36.7	[31.2, 41.9]	3283.2	25.4	-2.1	27.5	4257.7	16.9	-1.4	18.3	6229.9	8.5	-0.7	9.2	12060.7
Uruguay	14.6	[-39.6,62.2]	-13.1	[-61.0, 28.8]	27.7	[21.4, 33.4]	7626.1	10.9	-9.9	20.8	9885.3	7.3	-6.6	13.9	14353.8	3.6	-3.3	6.9	28285.9
Median	31.1		-4.3		34.4		3216.7	23.2	-3.3	25.8	4237.8	15.5	-2.2	17.15	6253.4	7.7	-1.2	8.6	12346.4

Table S3 Detailed reports on benefit risk and cost per DALY averted for each country modelled, in each region.

Africa

Kenya (KEN)

--- INPUTS ---

Based on the 2017 Global Burden of Disease analysis, the incidence rates of diarrhoea are: mild = 1.2 events per year per child; moderate = 0.65 per year per child; severe = 0.0093 per year per child.

Based on the 2017 Global Burden of Disease analysis, the incidence rates of malaria are: mild = 0.1 per year per child; moderate = 0.055 per year per child; severe = 0.0008 per year per child.

Based on the most recent Demographic and Health Survey (or equivalent), the prevalences of anaemia are:
6-8 months mild 31 percent, moderate 45 percent, severe 3.5 percent
9-11 months mild 31 percent, moderate 45 percent, severe 3.5 percent
12-17 months mild 30 percent, moderate 40 percent, severe 2.5 percent.

Background mortality rates through the treatment period are 0.861 percent per year on average.

Case-specific mortality rates are 0.0321 percent for a case of diarrhoea; 0.0722 percent for a case of malaria.

Kenya is classified as having endemic malaria, and has an Insecticide Treated bednet coverage rate of 54.3 percent based on 2015 UNICEF bednet coverage data.

In this country, the cost of an outpatient clinic visit is $1.69, and of hospitalisation is $6.5. We modelled the cost of MNPs as US $9.9 per course (including implementation).

--- OUTPUTS ---

Treatment with micronutrient powders caused a 4 percent increase in the incidence of diarrhoea, and a 3 percent increase in the incidence of malaria.
Treatment with micronutrient powders produced a 18 percent absolute decrease in the prevalence of anaemia at 12 months of age, and a 23 percent relative decrease.

Treatment with micronutrient powders caused an additional 0.473 deaths per 10,000 children [95 percent CIs = 1.99 to -0.853].

If coverage of the intervention is 100 percent, universal home fortification with micronutrient powders (MNPs) from age 6-12 months averted 26.2 [95 percent CIs = -76.5 to 117] DALYs per 10,000 children, compared with a control cohort. Of these averted DALYs, -29.7 [95 percent CIs = -125 to 53.4] were YLLs and 55.8 [95 percent CIs = 48.4 to 63.2] were YLDs.

When the effects of micronutrient powders on anaemia alone (excluding effects on diarrhoea and malaria) are considered, treatment with

micronutrient powders caused a change in YLDs per 10,000 children of 57.4.

Treatment with micronutrient powders caused a net increase/decrease in healthcare costs of USD $10.1 per DALY averted.

If coverage was:
* 100 percent, net DALYs per 10,000 children averted was 26.2.
* 75 percent, net DALYs per 10,000 children averted was 19.4.
* 50 percent, net DALYs per 10,000 children averted was 12.9.
* 25 percent, net DALYs per 10,000 children averted was 6.25.

If coverage was:
* 100 percent: cost per DALY averted was $3849.
* 75 percent: cost per DALY averted was $5163.
* 50 percent: cost per DALY averted was $7768.
* 25 percent: cost per DALY averted was $15916.

South Africa (ZAF)

--- INPUTS ---

Based on the 2017 Global Burden of Disease analysis, the incidence rates of diarrhoea are: mild = 0.18 events per year per child; moderate = 0.096 per year per child; severe = 0.0014 per year per child.

Based on the 2017 Global Burden of Disease analysis, the incidence rates of malaria are: mild = 1.2e-05 per year per child; moderate = 6.5e-06 per year per child; severe = 9.3e-08 per year per child.

Based on the most recent Demographic and Health Survey (or equivalent), the prevalences of anaemia are:
6-8 months mild 21 percent, moderate 28 percent, severe 0.75 percent
9-11 months mild 20 percent, moderate 31 percent, severe 2.4 percent
12-17 months mild 23 percent, moderate 35 percent, severe 0.67 percent.

Background mortality rates through the treatment period are 2.26 percent per year on average.

Case-specific mortality rates are 0.118 percent for a case of diarrhoea; 5.68 percent for a case of malaria.

South Africa is not classified as having endemic malaria, and has an Insecticide Treated bednet coverage rate of 0 percent based on 2015 UNICEF bednet coverage data.

In this country, the cost of an outpatient clinic visit is $6.13, and of hospitalisation is $43.2. We modelled the cost of MNPs as US $9.9 per course (including implementation).

--- OUTPUTS ---

Treatment with micronutrient powders caused a 5 percent increase in the incidence of diarrhoea, and a 2 percent increase in the incidence of malaria.

Treatment with micronutrient powders produced a 13 percent absolute decrease in the prevalence of anaemia at 12 months of age, and a 25 percent relative decrease.

Treatment with micronutrient powders caused an additional 0.0486 deaths per 10,000 children [95 percent CIs = 0.246 to -0.106].

If coverage of the intervention is 100 percent, universal home fortification with micronutrient powders (MNPs) from age 6-12 months averted 40.9 [95 percent CIs = 23.7 to 55.4] DALYs per 10,000 children, compared with a control cohort. Of these averted DALYs, -3.02 [95 percent CIs = -15.3 to 6.56] were YLLs and 43.9 [95 percent CIs = 39 to 48.9] were YLDs.

When the effects of micronutrient powders on anaemia alone (excluding effects on diarrhoea and malaria) are considered, treatment with micronutrient powders caused a change in YLDs per 10,000 children of 44.1.

Treatment with micronutrient powders caused a net increase/decrease in healthcare costs of USD $10 per DALY averted.

If coverage was:
* 100 percent, net DALYs per 10,000 children averted was 40.9.
* 75 percent, net DALYs per 10,000 children averted was 30.6.
* 50 percent, net DALYs per 10,000 children averted was 20.3.
* 25 percent, net DALYs per 10,000 children averted was 10.1.

If coverage was:
* 100 percent: cost per DALY averted was $2452.
* 75 percent: cost per DALY averted was $3269.
* 50 percent: cost per DALY averted was $4917.
* 25 percent: cost per DALY averted was $9832.

Namibia (NAM)

--- INPUTS ---

Based on the 2017 Global Burden of Disease analysis, the incidence rates of diarrhoea are: mild = 1.3 events per year per child; moderate = 0.71 per year per child; severe = 0.01 per year per child.

Based on the 2017 Global Burden of Disease analysis, the incidence rates of malaria are: mild = 0.0058 per year per child; moderate = 0.0031 per year per child; severe = 4.4e-05 per year per child.

Based on the most recent Demographic and Health Survey (or equivalent), the prevalences of anaemia are:
6-8 months mild 25 percent, moderate 34 percent, severe 0.9 percent
9-11 months mild 24 percent, moderate 37 percent, severe 2.9 percent
12-17 months mild 27 percent, moderate 42 percent, severe 0.8 percent.

Background mortality rates through the treatment period are 2.28 percent per year on average.

Case-specific mortality rates are 0.028 percent for a case of diarrhoea; 0.12 percent for a case of malaria.

Namibia is classified as having endemic malaria, and has an Insecticide Treated bednet coverage rate of 5.6 percent based on 2015 UNICEF bednet coverage data.

In this country, the cost of an outpatient clinic visit is $4.77, and of hospitalisation is $29. We modelled the cost of MNPs as US $9.9 per course (including implementation).

--- OUTPUTS ---

Treatment with micronutrient powders caused a 5 percent increase in the incidence of diarrhoea, and a 8 percent increase in the incidence of malaria.
Treatment with micronutrient powders produced a 17 percent absolute decrease in the prevalence of anaemia at 12 months of age, and a 27 percent relative decrease.

Treatment with micronutrient powders caused an additional 0.518 deaths per 10,000 children [95 percent CIs = 2.24 to -0.954].

If coverage of the intervention is 100 percent, universal home fortification with micronutrient powders (MNPs) from age 6-12 months averted 23.3 [95 percent CIs = -95.4 to 126] DALYs per 10,000 children, compared with a control cohort. Of these averted DALYs, -33.7 [95 percent CIs = -146 to 62] were YLLs and 57 [95 percent CIs = 50.2 to 63.8] were YLDs.

When the effects of micronutrient powders on anaemia alone (excluding effects on diarrhoea and malaria) are considered, treatment with micronutrient powders caused a change in YLDs per 10,000 children of 58.9.

Treatment with micronutrient powders caused a net increase/decrease in healthcare costs of USD $10.5 per DALY averted.

If coverage was:
* 100 percent, net DALYs per 10,000 children averted was 23.3.
* 75 percent, net DALYs per 10,000 children averted was 17.3.
* 50 percent, net DALYs per 10,000 children averted was 11.5.
* 25 percent, net DALYs per 10,000 children averted was 5.67.

If coverage was:
* 100 percent: cost per DALY averted was $4531.
* 75 percent: cost per DALY averted was $6013.
* 50 percent: cost per DALY averted was $8881.
* 25 percent: cost per DALY averted was $17732.

Botswana (BWA)

--- INPUTS ---

Based on the 2017 Global Burden of Disease analysis, the incidence rates of diarrhoea are: mild = 0.85 events per year per child; moderate = 0.45 per year per child; severe = 0.0065 per year per child.

Based on the 2017 Global Burden of Disease analysis, the incidence rates of malaria are: mild = 0.00013 per year per child; moderate = 6.8e-05 per year per child; severe = 9.8e-07 per year per child.

Based on the most recent Demographic and Health Survey (or equivalent), the prevalences of anaemia are:
6-8 months mild 22 percent, moderate 30 percent, severe 0.79 percent
9-11 months mild 21 percent, moderate 32 percent, severe 2.5 percent
12-17 months mild 24 percent, moderate 37 percent, severe 0.7 percent.

Background mortality rates through the treatment period are 2.42 percent per year on average.

Case-specific mortality rates are 0.0367 percent for a case of diarrhoea; 3.13 percent for a case of malaria.

Botswana is not classified as having endemic malaria, and has an Insecticide Treated bednet coverage rate of 0 percent based on 2015 UNICEF bednet coverage data.

In this country, the cost of an outpatient clinic visit is $8.42, and of hospitalisation is $65.8. We modelled the cost of MNPs as US $9.9 per course (including implementation).

--- OUTPUTS ---

Treatment with micronutrient powders caused a 5 percent increase in the incidence of diarrhoea, and a 3 percent decrease in the incidence of malaria.
Treatment with micronutrient powders produced a 12 percent absolute decrease in the prevalence of anaemia at 12 months of age, and a 22 percent relative decrease.

Treatment with micronutrient powders caused an additional 0.215 deaths per 10,000 children [95 percent CIs = 1.05 to -0.416].

If coverage of the intervention is 100 percent, universal home fortification with micronutrient powders (MNPs) from age 6-12 months averted 32.8 [95 percent CIs = -27.5 to 79.2] DALYs per 10,000 children, compared with a control cohort. Of these averted DALYs, -14 [95 percent CIs = -68.1 to 27] were YLLs and 46.8 [95 percent CIs = 40.6 to 52.1] were YLDs.

When the effects of micronutrient powders on anaemia alone (excluding effects on diarrhoea and malaria) are considered, treatment with micronutrient powders caused a change in YLDs per 10,000 children of 47.9.

Treatment with micronutrient powders caused a net increase/decrease in healthcare costs of USD $10.7 per DALY averted.

If coverage was:
* 100 percent, net DALYs per 10,000 children averted was 32.8.
* 75 percent, net DALYs per 10,000 children averted was 24.6.

* 50 percent, net DALYs per 10,000 children averted was 16.3.
* 25 percent, net DALYs per 10,000 children averted was 8.09.

If coverage was:
* 100 percent: cost per DALY averted was $3258.
* 75 percent: cost per DALY averted was $4267.
* 50 percent: cost per DALY averted was $6308.
* 25 percent: cost per DALY averted was $12529.

Zimbabawe (ZWE)

--- INPUTS ---

Based on the 2017 Global Burden of Disease analysis, the incidence rates of diarrhoea are: mild = 1.5 events per year per child; moderate = 0.83 per year per child; severe = 0.012 per year per child.

Based on the 2017 Global Burden of Disease analysis, the incidence rates of malaria are: mild = 0.029 per year per child; moderate = 0.016 per year per child; severe = 0.00023 per year per child.

Based on the most recent Demographic and Health Survey (or equivalent), the prevalences of anaemia are:
6-8 months mild 33 percent, moderate 28 percent, severe 0.9 percent
9-11 months mild 33 percent, moderate 28 percent, severe 0.9 percent
12-17 months mild 26 percent, moderate 26 percent, severe 0.9 percent.

Background mortality rates through the treatment period are 3.33 percent per year on average.

Case-specific mortality rates are 0.0216 percent for a case of diarrhoea; 0.377 percent for a case of malaria.

Zimbabawe is classified as having endemic malaria, and has an Insecticide Treated bednet coverage rate of 27 percent based on 2015 UNICEF bednet coverage data.

In this country, the cost of an outpatient clinic visit is $0, and of hospitalisation is $0. We modelled the cost of MNPs as US $9.9 per course (including implementation).

--- OUTPUTS ---

Treatment with micronutrient powders caused a 4 percent increase in the incidence of diarrhoea, and a 5 percent increase in the incidence of malaria.
Treatment with micronutrient powders produced a 18 percent absolute decrease in the prevalence of anaemia at 12 months of age, and a 29 percent relative decrease.

Treatment with micronutrient powders caused an additional 0.684 deaths per 10,000 children [95 percent CIs = 3.02 to -1.2].

If coverage of the intervention is 100 percent, universal home fortification with micronutrient powders (MNPs) from age 6-12 months

averted 0.233 [95 percent CIs = -147 to 121] DALYs per 10,000 children, compared with a control cohort. Of these averted DALYs, -41 [95 percent CIs = -181 to 72.2] were YLLs and 41.3 [95 percent CIs = 34.3 to 48.5] were YLDs.

When the effects of micronutrient powders on anaemia alone (excluding effects on diarrhoea and malaria) are considered, treatment with micronutrient powders caused a change in YLDs per 10,000 children of 43.2.

Treatment with micronutrient powders caused a net increase/decrease in healthcare costs of USD $9.9 per DALY averted.

If coverage was:
* 100 percent, net DALYs per 10,000 children averted was 0.233.
* 75 percent, net DALYs per 10,000 children averted was -0.299.
* 50 percent, net DALYs per 10,000 children averted was -0.151.
* 25 percent, net DALYs per 10,000 children averted was -0.408.

If coverage was:
* 100 percent: cost per DALY averted was $425697.
* 75 percent: cost per DALY averted was $-331608.
* 50 percent: cost per DALY averted was $-655533.
* 25 percent: cost per DALY averted was $-242599.

Zambia (ZMB)

--- INPUTS ---

Based on the 2017 Global Burden of Disease analysis, the incidence rates of diarrhoea are: mild = 1.5 events per year per child; moderate = 0.78 per year per child; severe = 0.011 per year per child.

Based on the 2017 Global Burden of Disease analysis, the incidence rates of malaria are: mild = 0.32 per year per child; moderate = 0.17 per year per child; severe = 0.0024 per year per child.

Based on the most recent Demographic and Health Survey (or equivalent), the prevalences of anaemia are:
6-8 months mild 40 percent, moderate 33 percent, severe 1.1 percent
9-11 months mild 40 percent, moderate 33 percent, severe 1.1 percent
12-17 months mild 32 percent, moderate 31 percent, severe 1.1 percent.

Background mortality rates through the treatment period are 3.07 percent per year on average.

Case-specific mortality rates are 0.0328 percent for a case of diarrhoea; 0.127 percent for a case of malaria.

Zambia is classified as having endemic malaria, and has an Insecticide Treated bednet coverage rate of 41 percent based on 2015 UNICEF bednet coverage data.

In this country, the cost of an outpatient clinic visit is $2.73, and of hospitalisation is $9.92. We modelled the cost of MNPs as US $9.9 per course (including implementation).

--- OUTPUTS ---

Treatment with micronutrient powders caused a 5 percent increase in the incidence of diarrhoea, and a 5 percent increase in the incidence of malaria.
Treatment with micronutrient powders produced a 18 percent absolute decrease in the prevalence of anaemia at 12 months of age, and a 24 percent relative decrease.

Treatment with micronutrient powders caused an additional 0.836 deaths per 10,000 children [95 percent CIs = 2.99 to -1.07].

If coverage of the intervention is 100 percent, universal home fortification with micronutrient powders (MNPs) from age 6-12 months averted -9.04 [95 percent CIs = -145 to 113] DALYs per 10,000 children, compared with a control cohort. Of these averted DALYs, -50.5 [95 percent CIs = -180 to 64.4] were YLLs and 41.5 [95 percent CIs = 34.1 to 48.7] were YLDs.

When the effects of micronutrient powders on anaemia alone (excluding effects on diarrhoea and malaria) are considered, treatment with micronutrient powders caused a change in YLDs per 10,000 children of 43.5.

Treatment with micronutrient powders caused a net increase/decrease in healthcare costs of USD $10.1 per DALY averted.

If coverage was:
* 100 percent, net DALYs per 10,000 children averted was -9.04.
* 75 percent, net DALYs per 10,000 children averted was -7.23.
* 50 percent, net DALYs per 10,000 children averted was -4.81.
* 25 percent, net DALYs per 10,000 children averted was -2.65.

[Because our model predicts that treatment with micronutrient powders increases net DALYs, we did not calculate the effect on healthcare costs when MNPs increase DALYs]

Angola (AGO)

--- INPUTS ---

Based on the 2017 Global Burden of Disease analysis, the incidence rates of diarrhoea are: mild = 1.7 events per year per child; moderate = 0.91 per year per child; severe = 0.013 per year per child.

Based on the 2017 Global Burden of Disease analysis, the incidence rates of malaria are: mild = 0.28 per year per child; moderate = 0.15 per year per child; severe = 0.0021 per year per child.

Based on the most recent Demographic and Health Survey (or equivalent), the prevalences of anaemia are:
6-8 months mild 27 percent, moderate 36 percent, severe 0.96 percent
9-11 months mild 25 percent, moderate 39 percent, severe 3.1 percent

12-17 months mild 29 percent, moderate 44 percent, severe 0.85 percent.

Background mortality rates through the treatment period are 7.05 percent per year on average.

Case-specific mortality rates are 0.0409 percent for a case of diarrhoea; 0.154 percent for a case of malaria.

Angola is classified as having endemic malaria, and has an Insecticide Treated bednet coverage rate of 25.9 percent based on 2015 UNICEF bednet coverage data.

In this country, the cost of an outpatient clinic visit is $7.09, and of hospitalisation is $41.1. We modelled the cost of MNPs as US $9.9 per course (including implementation).

--- OUTPUTS ---

Treatment with micronutrient powders caused a 5 percent increase in the incidence of diarrhoea, and a 6 percent increase in the incidence of malaria.
Treatment with micronutrient powders produced a 18 percent absolute decrease in the prevalence of anaemia at 12 months of age, and a 27 percent relative decrease.

Treatment with micronutrient powders caused an additional 2.08 deaths per 10,000 children [95 percent CIs = 5.07 to -0.779].

If coverage of the intervention is 100 percent, universal home fortification with micronutrient powders (MNPs) from age 6-12 months averted -45.4 [95 percent CIs = -208 to 109] DALYs per 10,000 children, compared with a control cohort. Of these averted DALYs, -108 [95 percent CIs = -262 to 40.2] were YLLs and 62.3 [95 percent CIs = 54 to 69.2] were YLDs.

When the effects of micronutrient powders on anaemia alone (excluding effects on diarrhoea and malaria) are considered, treatment with micronutrient powders caused a change in YLDs per 10,000 children of 64.8.

Treatment with micronutrient powders caused a net increase/decrease in healthcare costs of USD $11.3 per DALY averted.

If coverage was:
* 100 percent, net DALYs per 10,000 children averted was -45.4.
* 75 percent, net DALYs per 10,000 children averted was -34.9.
* 50 percent, net DALYs per 10,000 children averted was -23.3.
* 25 percent, net DALYs per 10,000 children averted was -12.3.

[Because our model predicts that treatment with micronutrient powders increases net DALYs, we did not calculate the effect on healthcare costs when MNPs increase DALYs]

Gabon (GAB)

--- INPUTS ---

Based on the 2017 Global Burden of Disease analysis, the incidence rates of diarrhoea are: mild = 1.8 events per year per child; moderate = 0.96 per year per child; severe = 0.014 per year per child.

Based on the 2017 Global Burden of Disease analysis, the incidence rates of malaria are: mild = 0.4 per year per child; moderate = 0.21 per year per child; severe = 0.0031 per year per child.

Based on the most recent Demographic and Health Survey (or equivalent), the prevalences of anaemia are:
6-8 months mild 32 percent, moderate 37 percent, severe 0.1 percent
9-11 months mild 36 percent, moderate 37 percent, severe 0.5 percent
12-17 months mild 34 percent, moderate 38 percent, severe 2.9 percent.

Background mortality rates through the treatment period are 2.54 percent per year on average.

Case-specific mortality rates are 0.00611 percent for a case of diarrhoea; 0.143 percent for a case of malaria.

Gabon is classified as having endemic malaria, and has an Insecticide Treated bednet coverage rate of 38.8 percent based on 2015 UNICEF bednet coverage data.

In this country, the cost of an outpatient clinic visit is $10.5, and of hospitalisation is $83.9. We modelled the cost of MNPs as US $9.9 per course (including implementation).

--- OUTPUTS ---

Treatment with micronutrient powders caused a 5 percent increase in the incidence of diarrhoea, and a 5 percent increase in the incidence of malaria.
Treatment with micronutrient powders produced a 19 percent absolute decrease in the prevalence of anaemia at 12 months of age, and a 27 percent relative decrease.

Treatment with micronutrient powders caused an additional 0.57 deaths per 10,000 children [95 percent CIs = 1.53 to -0.316].

If coverage of the intervention is 100 percent, universal home fortification with micronutrient powders (MNPs) from age 6-12 months averted 18.2 [95 percent CIs = -52.1 to 83.6] DALYs per 10,000 children, compared with a control cohort. Of these averted DALYs, -37.2 [95 percent CIs = -99.6 to 20.6] were YLLs and 55.4 [95 percent CIs = 47.5 to 63] were YLDs.

When the effects of micronutrient powders on anaemia alone (excluding effects on diarrhoea and malaria) are considered, treatment with micronutrient powders caused a change in YLDs per 10,000 children of 57.9.

Treatment with micronutrient powders caused a net increase/decrease in healthcare costs of USD $12 per DALY averted.

If coverage was:
* 100 percent, net DALYs per 10,000 children averted was 18.2.
* 75 percent, net DALYs per 10,000 children averted was 13.3.

* 50 percent, net DALYs per 10,000 children averted was 8.89.
* 25 percent, net DALYs per 10,000 children averted was 4.15.

If coverage was:
* 100 percent: cost per DALY averted was $6565.
* 75 percent: cost per DALY averted was $8622.
* 50 percent: cost per DALY averted was $12246.
* 25 percent: cost per DALY averted was $25200.

Uganda (UGA)

--- INPUTS ---

Based on the 2017 Global Burden of Disease analysis, the incidence rates of diarrhoea are: mild = 1.3 events per year per child; moderate = 0.68 per year per child; severe = 0.0098 per year per child.

Based on the 2017 Global Burden of Disease analysis, the incidence rates of malaria are: mild = 0.52 per year per child; moderate = 0.28 per year per child; severe = 0.004 per year per child.

Based on the most recent Demographic and Health Survey (or equivalent), the prevalences of anaemia are:
6-8 months mild 22 percent, moderate 41 percent, severe 3.4 percent
9-11 months mild 25 percent, moderate 42 percent, severe 2.3 percent
12-17 months mild 32 percent, moderate 30 percent, severe 3.5 percent.

Background mortality rates through the treatment period are 2.69 percent per year on average.

Case-specific mortality rates are 0.0196 percent for a case of diarrhoea; 0.169 percent for a case of malaria.

Uganda is classified as having endemic malaria, and has an Insecticide Treated bednet coverage rate of 74.3 percent based on 2015 UNICEF bednet coverage data.

In this country, the cost of an outpatient clinic visit is $0.877, and of hospitalisation is $3.1. We modelled the cost of MNPs as US $9.9 per course (including implementation).

--- OUTPUTS ---

Treatment with micronutrient powders caused a 5 percent increase in the incidence of diarrhoea, and a 0.8 percent increase in the incidence of malaria.
Treatment with micronutrient powders produced a 19 percent absolute decrease in the prevalence of anaemia at 12 months of age, and a 28 percent relative decrease.

Treatment with micronutrient powders caused an additional 0.539 deaths per 10,000 children [95 percent CIs = 2.35 to -1.12].

If coverage of the intervention is 100 percent, universal home fortification with micronutrient powders (MNPs) from age 6-12 months

averted 28 [95 percent CIs = -92 to 137] DALYs per 10,000 children, compared with a control cohort. Of these averted DALYs, -33.2 [95 percent CIs = -145 to 68.7] were YLLs and 61.2 [95 percent CIs = 52.9 to 68.1] were YLDs.

When the effects of micronutrient powders on anaemia alone (excluding effects on diarrhoea and malaria) are considered, treatment with micronutrient powders caused a change in YLDs per 10,000 children of 63.2.

Treatment with micronutrient powders caused a net increase/decrease in healthcare costs of USD $10 per DALY averted.

If coverage was:
* 100 percent, net DALYs per 10,000 children averted was 28.
* 75 percent, net DALYs per 10,000 children averted was 20.5.
* 50 percent, net DALYs per 10,000 children averted was 13.7.
* 25 percent, net DALYs per 10,000 children averted was 6.52.

If coverage was:
* 100 percent: cost per DALY averted was $3570.
* 75 percent: cost per DALY averted was $4859.
* 50 percent: cost per DALY averted was $7279.
* 25 percent: cost per DALY averted was $15221.

Ethiopia (ETH)

--- INPUTS ---

Based on the 2017 Global Burden of Disease analysis, the incidence rates of diarrhoea are: mild = 1.1 events per year per child; moderate = 0.61 per year per child; severe = 0.0088 per year per child.

Based on the 2017 Global Burden of Disease analysis, the incidence rates of malaria are: mild = 0.021 per year per child; moderate = 0.011 per year per child; severe = 0.00016 per year per child.

Based on the most recent Demographic and Health Survey (or equivalent), the prevalences of anaemia are:
6-8 months mild 24 percent, moderate 36 percent, severe 1.5 percent
9-11 months mild 26 percent, moderate 41 percent, severe 5.7 percent
12-17 months mild 25 percent, moderate 34 percent, severe 3.6 percent.

Background mortality rates through the treatment period are 2.9 percent per year on average.

Case-specific mortality rates are 0.0521 percent for a case of diarrhoea; 0.122 percent for a case of malaria.

Ethiopia is not classified as having endemic malaria, and has an Insecticide Treated bednet coverage rate of 30.1 percent based on 2015 UNICEF bednet coverage data.

In this country, the cost of an outpatient clinic visit is $0.726, and of hospitalisation is $2.24. We modelled the cost of MNPs as US $9.9 per course (including implementation).

--- OUTPUTS ---

Treatment with micronutrient powders caused a 4 percent increase in the incidence of diarrhoea, and a 5 percent decrease in the incidence of malaria.
Treatment with micronutrient powders produced a 16 percent absolute decrease in the prevalence of anaemia at 12 months of age, and a 22 percent relative decrease.

Treatment with micronutrient powders caused an additional 0.348 deaths per 10,000 children [95 percent CIs = 1.74 to -0.902].

If coverage of the intervention is 100 percent, universal home fortification with micronutrient powders (MNPs) from age 6-12 months averted 29.6 [95 percent CIs = -67.2 to 117] DALYs per 10,000 children, compared with a control cohort. Of these averted DALYs, -22.3 [95 percent CIs = -111 to 57.7] were YLLs and 51.8 [95 percent CIs = 44.3 to 58.9] were YLDs.

When the effects of micronutrient powders on anaemia alone (excluding effects on diarrhoea and malaria) are considered, treatment with micronutrient powders caused a change in YLDs per 10,000 children of 53.3.

Treatment with micronutrient powders caused a net increase/decrease in healthcare costs of USD $9.97 per DALY averted.

If coverage was:
* 100 percent, net DALYs per 10,000 children averted was 29.6.
* 75 percent, net DALYs per 10,000 children averted was 21.5.
* 50 percent, net DALYs per 10,000 children averted was 14.5.
* 25 percent, net DALYs per 10,000 children averted was 6.82.

If coverage was:
* 100 percent: cost per DALY averted was $3371.
* 75 percent: cost per DALY averted was $4627.
* 50 percent: cost per DALY averted was $6872.
* 25 percent: cost per DALY averted was $14527.

Eritrea (ERI)

--- INPUTS ---

Based on the 2017 Global Burden of Disease analysis, the incidence rates of diarrhoea are: mild = 1.2 events per year per child; moderate = 0.66 per year per child; severe = 0.0095 per year per child.

Based on the 2017 Global Burden of Disease analysis, the incidence rates of malaria are: mild = 0.0042 per year per child; moderate = 0.0022 per year per child; severe = 3.2e-05 per year per child.

Based on the most recent Demographic and Health Survey (or equivalent), the prevalences of anaemia are:
6-8 months mild 29 percent, moderate 42 percent, severe 1.8 percent
9-11 months mild 31 percent, moderate 48 percent, severe 6.7 percent
12-17 months mild 30 percent, moderate 40 percent, severe 4.2 percent.

Background mortality rates through the treatment period are 2.39 percent per year on average.

Case-specific mortality rates are 0.0626 percent for a case of diarrhoea; 0.0259 percent for a case of malaria.

Eritrea is classified as having endemic malaria, and has an Insecticide Treated bednet coverage rate of 20 percent based on 2015 UNICEF bednet coverage data.

In this country, the cost of an outpatient clinic visit is $1.93, and of hospitalisation is $5.55. We modelled the cost of MNPs as US $9.9 per course (including implementation).

--- OUTPUTS ---

Treatment with micronutrient powders caused a 4 percent increase in the incidence of diarrhoea, and a 7 percent increase in the incidence of malaria.
Treatment with micronutrient powders produced a 23 percent absolute decrease in the prevalence of anaemia at 12 months of age, and a 26 percent relative decrease.

Treatment with micronutrient powders caused an additional 1.06 deaths per 10,000 children [95 percent CIs = 4.93 to -2.24].

If coverage of the intervention is 100 percent, universal home fortification with micronutrient powders (MNPs) from age 6-12 months averted 4.54 [95 percent CIs = -251 to 223] DALYs per 10,000 children, compared with a control cohort. Of these averted DALYs, -67.7 [95 percent CIs = -315 to 143] were YLLs and 72.2 [95 percent CIs = 64.7 to 79.7] were YLDs.

When the effects of micronutrient powders on anaemia alone (excluding effects on diarrhoea and malaria) are considered, treatment with micronutrient powders caused a change in YLDs per 10,000 children of 73.8.

Treatment with micronutrient powders caused a net increase/decrease in healthcare costs of USD $10.1 per DALY averted.

If coverage was:
* 100 percent, net DALYs per 10,000 children averted was 4.54.
* 75 percent, net DALYs per 10,000 children averted was 2.62.
* 50 percent, net DALYs per 10,000 children averted was 1.73.
* 25 percent, net DALYs per 10,000 children averted was 0.363.

If coverage was:
* 100 percent: cost per DALY averted was $22196.
* 75 percent: cost per DALY averted was $38251.
* 50 percent: cost per DALY averted was $57620.

* 25 percent: cost per DALY averted was $274175.

Madagascar (MDG)

--- INPUTS ---

Based on the 2017 Global Burden of Disease analysis, the incidence rates of diarrhoea are: mild = 1.8 events per year per child; moderate = 0.97 per year per child; severe = 0.014 per year per child.

Based on the 2017 Global Burden of Disease analysis, the incidence rates of malaria are: mild = 0.12 per year per child; moderate = 0.065 per year per child; severe = 0.00093 per year per child.

Based on the most recent Demographic and Health Survey (or equivalent), the prevalences of anaemia are:
6-8 months mild 32 percent, moderate 37 percent, severe 0.1 percent
9-11 months mild 36 percent, moderate 37 percent, severe 0.5 percent
12-17 months mild 34 percent, moderate 38 percent, severe 2.9 percent.

Background mortality rates through the treatment period are 2.5 percent per year on average.

Case-specific mortality rates are 0.0636 percent for a case of diarrhoea; 0.164 percent for a case of malaria.

Madagascar is classified as having endemic malaria, and has an Insecticide Treated bednet coverage rate of 62.3 percent based on 2015 UNICEF bednet coverage data.

In this country, the cost of an outpatient clinic visit is $0.768, and of hospitalisation is $2.56. We modelled the cost of MNPs as US $9.9 per course (including implementation).

--- OUTPUTS ---

Treatment with micronutrient powders caused a 5 percent increase in the incidence of diarrhoea, and a 3 percent increase in the incidence of malaria.
Treatment with micronutrient powders produced a 19 percent absolute decrease in the prevalence of anaemia at 12 months of age, and a 26 percent relative decrease.

Treatment with micronutrient powders caused an additional 1.08 deaths per 10,000 children [95 percent CIs = 4.28 to -1.82].

If coverage of the intervention is 100 percent, universal home fortification with micronutrient powders (MNPs) from age 6-12 months averted -14.1 [95 percent CIs = -229 to 181] DALYs per 10,000 children, compared with a control cohort. Of these averted DALYs, -69.6 [95 percent CIs = -277 to 118] were YLLs and 55.5 [95 percent CIs = 47.7 to 63.4] were YLDs.

When the effects of micronutrient powders on anaemia alone (excluding effects on diarrhoea and malaria) are considered, treatment with

micronutrient powders caused a change in YLDs per 10,000 children of 58.

Treatment with micronutrient powders caused a net increase/decrease in healthcare costs of USD $10 per DALY averted.

If coverage was:
* 100 percent, net DALYs per 10,000 children averted was -14.1.
* 75 percent, net DALYs per 10,000 children averted was -11.2.
* 50 percent, net DALYs per 10,000 children averted was -7.48.
* 25 percent, net DALYs per 10,000 children averted was -4.09.

[Because our model predicts that treatment with micronutrient powders increases net DALYs, we did not calculate the effect on healthcare costs when MNPs increase DALYs]

Djibouti (DJI)

--- INPUTS ---

Based on the 2017 Global Burden of Disease analysis, the incidence rates of diarrhoea are: mild = 0.84 events per year per child; moderate = 0.45 per year per child; severe = 0.0065 per year per child.

Based on the 2017 Global Burden of Disease analysis, the incidence rates of malaria are: mild = 0.019 per year per child; moderate = 0.01 per year per child; severe = 0.00015 per year per child.

Based on the most recent Demographic and Health Survey (or equivalent), the prevalences of anaemia are:
6-8 months mild 21 percent, moderate 31 percent, severe 1.3 percent
9-11 months mild 23 percent, moderate 35 percent, severe 4.9 percent
12-17 months mild 22 percent, moderate 29 percent, severe 3.1 percent.

Background mortality rates through the treatment period are 3.73 percent per year on average.

Case-specific mortality rates are 0.0261 percent for a case of diarrhoea; 0.0022 percent for a case of malaria.

Djibouti is not classified as having endemic malaria, and has an Insecticide Treated bednet coverage rate of 20 percent based on 2015 UNICEF bednet coverage data.

In this country, the cost of an outpatient clinic visit is $2.44, and of hospitalisation is $10.4. We modelled the cost of MNPs as US $9.9 per course (including implementation).

--- OUTPUTS ---

Treatment with micronutrient powders caused a 5 percent increase in the incidence of diarrhoea, and a 4 percent decrease in the incidence of malaria.
Treatment with micronutrient powders produced a 16 percent absolute decrease in the prevalence of anaemia at 12 months of age, and a 25 percent relative decrease.

Treatment with micronutrient powders caused an additional 0.334 deaths per 10,000 children [95 percent CIs = 1.56 to -0.737].

If coverage of the intervention is 100 percent, universal home fortification with micronutrient powders (MNPs) from age 6-12 months averted 28.2 [95 percent CIs = -55 to 101] DALYs per 10,000 children, compared with a control cohort. Of these averted DALYs, -21 [95 percent CIs = -98.1 to 46.2] were YLLs and 49.2 [95 percent CIs = 43.1 to 55.2] were YLDs.

When the effects of micronutrient powders on anaemia alone (excluding effects on diarrhoea and malaria) are considered, treatment with micronutrient powders caused a change in YLDs per 10,000 children of 50.3.

Treatment with micronutrient powders caused a net increase/decrease in healthcare costs of USD $10.1 per DALY averted.

If coverage was:
* 100 percent, net DALYs per 10,000 children averted was 28.2.
* 75 percent, net DALYs per 10,000 children averted was 20.9.
* 50 percent, net DALYs per 10,000 children averted was 13.9.
* 25 percent, net DALYs per 10,000 children averted was 6.82.

If coverage was:
* 100 percent: cost per DALY averted was $3576.
* 75 percent: cost per DALY averted was $4800.
* 50 percent: cost per DALY averted was $7206.
* 25 percent: cost per DALY averted was $14592.

Mozambique (MOZ)

--- INPUTS ---

Based on the 2017 Global Burden of Disease analysis, the incidence rates of diarrhoea are: mild = 0.97 events per year per child; moderate = 0.52 per year per child; severe = 0.0075 per year per child.

Based on the 2017 Global Burden of Disease analysis, the incidence rates of malaria are: mild = 0.52 per year per child; moderate = 0.28 per year per child; severe = 0.004 per year per child.

Based on the most recent Demographic and Health Survey (or equivalent), the prevalences of anaemia are:
6-8 months mild 28 percent, moderate 44 percent, severe 9 percent
9-11 months mild 27 percent, moderate 47 percent, severe 7 percent
12-17 months mild 23 percent, moderate 50 percent, severe 7.8 percent.

Background mortality rates through the treatment period are 4.02 percent per year on average.

Case-specific mortality rates are 0.0305 percent for a case of diarrhoea; 0.155 percent for a case of malaria.

Mozambique is classified as having endemic malaria, and has an Insecticide Treated bednet coverage rate of 35.7 percent based on 2015 UNICEF bednet coverage data.

In this country, the cost of an outpatient clinic visit is $0.619, and of hospitalisation is $1.91. We modelled the cost of MNPs as US $9.9 per course (including implementation).

--- OUTPUTS ---

Treatment with micronutrient powders caused a 5 percent increase in the incidence of diarrhoea, and a 5 percent increase in the incidence of malaria.
Treatment with micronutrient powders produced a 20 percent absolute decrease in the prevalence of anaemia at 12 months of age, and a 24 percent relative decrease.

Treatment with micronutrient powders caused an additional 1.61 deaths per 10,000 children [95 percent CIs = 3.6 to -0.483].

If coverage of the intervention is 100 percent, universal home fortification with micronutrient powders (MNPs) from age 6-12 months averted -13.8 [95 percent CIs = -134 to 113] DALYs per 10,000 children, compared with a control cohort. Of these averted DALYs, -91.3 [95 percent CIs = -205 to 27.4] were YLLs and 77.5 [95 percent CIs = 70.3 to 85.4] were YLDs.

When the effects of micronutrient powders on anaemia alone (excluding effects on diarrhoea and malaria) are considered, treatment with micronutrient powders caused a change in YLDs per 10,000 children of 79.1.

Treatment with micronutrient powders caused a net increase/decrease in healthcare costs of USD $9.97 per DALY averted.

If coverage was:
* 100 percent, net DALYs per 10,000 children averted was -13.8.
* 75 percent, net DALYs per 10,000 children averted was -10.7.
* 50 percent, net DALYs per 10,000 children averted was -7.11.
* 25 percent, net DALYs per 10,000 children averted was -4.

[Because our model predicts that treatment with micronutrient powders increases net DALYs, we did not calculate the effect on healthcare costs when MNPs increase DALYs]
Tanzania (TZA)

--- INPUTS ---

Based on the 2017 Global Burden of Disease analysis, the incidence rates of diarrhoea are: mild = 0.98 events per year per child; moderate = 0.53 per year per child; severe = 0.0076 per year per child.

Based on the 2017 Global Burden of Disease analysis, the incidence rates of malaria are: mild = 0.21 per year per child; moderate = 0.11 per year per child; severe = 0.0016 per year per child.

Based on the most recent Demographic and Health Survey (or equivalent), the prevalences of anaemia are:
6-8 months mild 31 percent, moderate 45 percent, severe 3.5 percent
9-11 months mild 31 percent, moderate 45 percent, severe 3.5 percent
12-17 months mild 30 percent, moderate 40 percent, severe 2.5 percent.

Background mortality rates through the treatment period are 2.46 percent per year on average.

Case-specific mortality rates are 0.0189 percent for a case of diarrhoea; 0.201 percent for a case of malaria.

Tanzania is classified as having endemic malaria, and has an Insecticide Treated bednet coverage rate of 72 percent based on 2015 UNICEF bednet coverage data.

In this country, the cost of an outpatient clinic visit is $0.988, and of hospitalisation is $3.49. We modelled the cost of MNPs as US $9.9 per course (including implementation).

--- OUTPUTS ---

Treatment with micronutrient powders caused a 5 percent increase in the incidence of diarrhoea, and a 0.8 percent increase in the incidence of malaria.
Treatment with micronutrient powders produced a 18 percent absolute decrease in the prevalence of anaemia at 12 months of age, and a 23 percent relative decrease.

Treatment with micronutrient powders caused an additional 0.254 deaths per 10,000 children [95 percent CIs = 1.23 to -0.622].

If coverage of the intervention is 100 percent, universal home fortification with micronutrient powders (MNPs) from age 6-12 months averted 40.6 [95 percent CIs = -25.7 to 101] DALYs per 10,000 children, compared with a control cohort. Of these averted DALYs, -15.5 [95 percent CIs = -75.1 to 38] were YLLs and 56.1 [95 percent CIs = 49.4 to 62.9] were YLDs.

When the effects of micronutrient powders on anaemia alone (excluding effects on diarrhoea and malaria) are considered, treatment with micronutrient powders caused a change in YLDs per 10,000 children of 57.5.

Treatment with micronutrient powders caused a net increase/decrease in healthcare costs of USD $9.99 per DALY averted.

If coverage was:
* 100 percent, net DALYs per 10,000 children averted was 40.6.
* 75 percent, net DALYs per 10,000 children averted was 30.
* 50 percent, net DALYs per 10,000 children averted was 19.9.
* 25 percent, net DALYs per 10,000 children averted was 9.7.

If coverage was:
* 100 percent: cost per DALY averted was $2462.
* 75 percent: cost per DALY averted was $3321.
* 50 percent: cost per DALY averted was $4990.

* 25 percent: cost per DALY averted was $10227.

Democratic Republic of Congo (COD)

--- INPUTS ---

Based on the 2017 Global Burden of Disease analysis, the incidence rates of diarrhoea are: mild = 1.7 events per year per child; moderate = 0.92 per year per child; severe = 0.013 per year per child.

Based on the 2017 Global Burden of Disease analysis, the incidence rates of malaria are: mild = 0.55 per year per child; moderate = 0.3 per year per child; severe = 0.0043 per year per child.

Based on the most recent Demographic and Health Survey (or equivalent), the prevalences of anaemia are:
6-8 months mild 28 percent, moderate 46 percent, severe 3 percent
9-11 months mild 22 percent, moderate 44 percent, severe 3.5 percent
12-17 months mild 29 percent, moderate 34 percent, severe 4.6 percent.

Background mortality rates through the treatment period are 5.24 percent per year on average.

Case-specific mortality rates are 0.0272 percent for a case of diarrhoea; 0.33 percent for a case of malaria.

Democratic Republic of Congo is classified as having endemic malaria, and has an Insecticide Treated bednet coverage rate of 55.8 percent based on 2015 UNICEF bednet coverage data.

In this country, the cost of an outpatient clinic visit is $0.239, and of hospitalisation is $0.538. We modelled the cost of MNPs as US $9.9 per course (including implementation).

--- OUTPUTS ---

Treatment with micronutrient powders caused a 4 percent increase in the incidence of diarrhoea, and a 3 percent increase in the incidence of malaria.
Treatment with micronutrient powders produced a 15 percent absolute decrease in the prevalence of anaemia at 12 months of age, and a 23 percent relative decrease.

Treatment with micronutrient powders caused an additional 1.41 deaths per 10,000 children [95 percent CIs = 4.09 to -1.24].

If coverage of the intervention is 100 percent, universal home fortification with micronutrient powders (MNPs) from age 6-12 months averted -23.2 [95 percent CIs = -191 to 141] DALYs per 10,000 children, compared with a control cohort. Of these averted DALYs, -83.2 [95 percent CIs = -242 to 73.5] were YLLs and 60 [95 percent CIs = 50.6 to 67.8] were YLDs.

When the effects of micronutrient powders on anaemia alone (excluding effects on diarrhoea and malaria) are considered, treatment with

micronutrient powders caused a change in YLDs per 10,000 children of 62.3.

Treatment with micronutrient powders caused a net increase/decrease in healthcare costs of USD $9.94 per DALY averted.

If coverage was:
* 100 percent, net DALYs per 10,000 children averted was -23.2.
* 75 percent, net DALYs per 10,000 children averted was -18.2.
* 50 percent, net DALYs per 10,000 children averted was -12.1.
* 25 percent, net DALYs per 10,000 children averted was -6.6.

[Because our model predicts that treatment with micronutrient powders increases net DALYs, we did not calculate the effect on healthcare costs when MNPs increase DALYs]

Central African Republic (CAF)

--- INPUTS ---

Based on the 2017 Global Burden of Disease analysis, the incidence rates of diarrhoea are: mild = 2 events per year per child; moderate = 1.1 per year per child; severe = 0.015 per year per child.

Based on the 2017 Global Burden of Disease analysis, the incidence rates of malaria are: mild = 0.63 per year per child; moderate = 0.33 per year per child; severe = 0.0048 per year per child.

Based on the most recent Demographic and Health Survey (or equivalent), the prevalences of anaemia are:
6-8 months mild 30 percent, moderate 49 percent, severe 3.2 percent
9-11 months mild 23 percent, moderate 47 percent, severe 3.8 percent
12-17 months mild 31 percent, moderate 37 percent, severe 4.9 percent.

Background mortality rates through the treatment period are 6.53 percent per year on average.

Case-specific mortality rates are 0.116 percent for a case of diarrhoea; 0.251 percent for a case of malaria.

Central African Republic is classified as having endemic malaria, and has an Insecticide Treated bednet coverage rate of 36 percent based on 2015 UNICEF bednet coverage data.

In this country, the cost of an outpatient clinic visit is $1, and of hospitalisation is $3.02. We modelled the cost of MNPs as US $9.9 per course (including implementation).

--- OUTPUTS ---

Treatment with micronutrient powders caused a 4 percent increase in the incidence of diarrhoea, and a 5 percent increase in the incidence of malaria.
Treatment with micronutrient powders produced a 17 percent absolute decrease in the prevalence of anaemia at 12 months of age, and a 23 percent relative decrease.

Treatment with micronutrient powders caused an additional 3.31 deaths per 10,000 children [95 percent CIs = 8.06 to -1.09].

If coverage of the intervention is 100 percent, universal home fortification with micronutrient powders (MNPs) from age 6-12 months averted -113 [95 percent CIs = -369 to 123] DALYs per 10,000 children, compared with a control cohort. Of these averted DALYs, -171 [95 percent CIs = -417 to 56.4] were YLLs and 58.2 [95 percent CIs = 48.2 to 66.9] were YLDs.

When the effects of micronutrient powders on anaemia alone (excluding effects on diarrhoea and malaria) are considered, treatment with micronutrient powders caused a change in YLDs per 10,000 children of 60.9.

Treatment with micronutrient powders caused a net increase/decrease in healthcare costs of USD $10.1 per DALY averted.

If coverage was:
* 100 percent, net DALYs per 10,000 children averted was -113.
* 75 percent, net DALYs per 10,000 children averted was -85.8.
* 50 percent, net DALYs per 10,000 children averted was -57.2.
* 25 percent, net DALYs per 10,000 children averted was -29.

[Because our model predicts that treatment with micronutrient powders increases net DALYs, we did not calculate the effect on healthcare costs when MNPs increase DALYs]

Cameroon (CMR)

--- INPUTS ---

Based on the 2017 Global Burden of Disease analysis, the incidence rates of diarrhoea are: mild = 1.9 events per year per child; moderate = 1 per year per child; severe = 0.015 per year per child.

Based on the 2017 Global Burden of Disease analysis, the incidence rates of malaria are: mild = 0.47 per year per child; moderate = 0.25 per year per child; severe = 0.0036 per year per child.

Based on the most recent Demographic and Health Survey (or equivalent), the prevalences of anaemia are:
6-8 months mild 36 percent, moderate 42 percent, severe 1.2 percent
9-11 months mild 27 percent, moderate 42 percent, severe 4.4 percent
12-17 months mild 30 percent, moderate 41 percent, severe 2.2 percent.

Background mortality rates through the treatment period are 4.07 percent per year on average.

Case-specific mortality rates are 0.0368 percent for a case of diarrhoea; 0.292 percent for a case of malaria.

Cameroon is classified as having endemic malaria, and has an Insecticide Treated bednet coverage rate of 55 percent based on 2015 UNICEF bednet coverage data.

In this country, the cost of an outpatient clinic visit is $1.91, and of hospitalisation is $8.14. We modelled the cost of MNPs as US $9.9 per course (including implementation).

--- OUTPUTS ---

Treatment with micronutrient powders caused a 5 percent increase in the incidence of diarrhoea, and a 3 percent increase in the incidence of malaria.
Treatment with micronutrient powders produced a 17 percent absolute decrease in the prevalence of anaemia at 12 months of age, and a 24 percent relative decrease.

Treatment with micronutrient powders caused an additional 2 deaths per 10,000 children [95 percent CIs = 6.1 to -1.7].

If coverage of the intervention is 100 percent, universal home fortification with micronutrient powders (MNPs) from age 6-12 months averted -56.5 [95 percent CIs = -297 to 161] DALYs per 10,000 children, compared with a control cohort. Of these averted DALYs, -113 [95 percent CIs = -345 to 96] were YLLs and 56.5 [95 percent CIs = 47.5 to 65] were YLDs.

When the effects of micronutrient powders on anaemia alone (excluding effects on diarrhoea and malaria) are considered, treatment with micronutrient powders caused a change in YLDs per 10,000 children of 59.2.

Treatment with micronutrient powders caused a net increase/decrease in healthcare costs of USD $10.2 per DALY averted.

If coverage was:
* 100 percent, net DALYs per 10,000 children averted was -56.5.
* 75 percent, net DALYs per 10,000 children averted was -43.3.
* 50 percent, net DALYs per 10,000 children averted was -28.9.
* 25 percent, net DALYs per 10,000 children averted was -15.1.

[Because our model predicts that treatment with micronutrient powders increases net DALYs, we did not calculate the effect on healthcare costs when MNPs increase DALYs]

Chad (TCD)

--- INPUTS ---

Based on the 2017 Global Burden of Disease analysis, the incidence rates of diarrhoea are: mild = 2.4 events per year per child; moderate = 1.3 per year per child; severe = 0.018 per year per child.

Based on the 2017 Global Burden of Disease analysis, the incidence rates of malaria are: mild = 0.28 per year per child; moderate = 0.15 per year per child; severe = 0.0021 per year per child.

Based on the most recent Demographic and Health Survey (or equivalent), the prevalences of anaemia are:
6-8 months mild 30 percent, moderate 55 percent, severe 4.8 percent
9-11 months mild 24 percent, moderate 62 percent, severe 6.2 percent

12-17 months mild 23 percent, moderate 71 percent, severe 3.4 percent.

Background mortality rates through the treatment period are 6.22 percent per year on average.

Case-specific mortality rates are 0.127 percent for a case of diarrhoea; 0.282 percent for a case of malaria.

Chad is classified as having endemic malaria, and has an Insecticide Treated bednet coverage rate of 36 percent based on 2015 UNICEF bednet coverage data.

In this country, the cost of an outpatient clinic visit is $1.34, and of hospitalisation is $4.93. We modelled the cost of MNPs as US $9.9 per course (including implementation).

--- OUTPUTS ---

Treatment with micronutrient powders caused a 5 percent increase in the incidence of diarrhoea, and a 5 percent increase in the incidence of malaria.
Treatment with micronutrient powders produced a 22 percent absolute decrease in the prevalence of anaemia at 12 months of age, and a 23 percent relative decrease.

Treatment with micronutrient powders caused an additional 5.7 deaths per 10,000 children [95 percent CIs = 20.2 to -5.73].

If coverage of the intervention is 100 percent, universal home fortification with micronutrient powders (MNPs) from age 6-12 months averted -220 [95 percent CIs = -992 to 388] DALYs per 10,000 children, compared with a control cohort. Of these averted DALYs, -298 [95 percent CIs = -1.06e+03 to 300] were YLLs and 78.2 [95 percent CIs = 66.8 to 88.2] were YLDs.

When the effects of micronutrient powders on anaemia alone (excluding effects on diarrhoea and malaria) are considered, treatment with micronutrient powders caused a change in YLDs per 10,000 children of 81.5.

Treatment with micronutrient powders caused a net increase/decrease in healthcare costs of USD $10.2 per DALY averted.

If coverage was:
* 100 percent, net DALYs per 10,000 children averted was -220.
* 75 percent, net DALYs per 10,000 children averted was -167.
* 50 percent, net DALYs per 10,000 children averted was -111.
* 25 percent, net DALYs per 10,000 children averted was -56.6.

[Because our model predicts that treatment with micronutrient powders increases net DALYs, we did not calculate the effect on healthcare costs when MNPs increase DALYs]

Nigeria (NGA)

--- INPUTS ---

Based on the 2017 Global Burden of Disease analysis, the incidence rates of diarrhoea are: mild = 1.1 events per year per child; moderate = 0.58 per year per child; severe = 0.0084 per year per child.

Based on the 2017 Global Burden of Disease analysis, the incidence rates of malaria are: mild = 0.37 per year per child; moderate = 0.2 per year per child; severe = 0.0029 per year per child.

Based on the most recent Demographic and Health Survey (or equivalent), the prevalences of anaemia are:
6-8 months mild 26 percent, moderate 47 percent, severe 4.1 percent
9-11 months mild 21 percent, moderate 54 percent, severe 5.3 percent
12-17 months mild 20 percent, moderate 61 percent, severe 2.9 percent.

Background mortality rates through the treatment period are 5.03 percent per year on average.

Case-specific mortality rates are 0.106 percent for a case of diarrhoea; 0.471 percent for a case of malaria.

Nigeria is classified as having endemic malaria, and has an Insecticide Treated bednet coverage rate of 16.6 percent based on 2015 UNICEF bednet coverage data.

In this country, the cost of an outpatient clinic visit is $2.78, and of hospitalisation is $11.6. We modelled the cost of MNPs as US $9.9 per course (including implementation).

--- OUTPUTS ---

Treatment with micronutrient powders caused a 6 percent increase in the incidence of diarrhoea, and a 7 percent increase in the incidence of malaria.
Treatment with micronutrient powders produced a 19 percent absolute decrease in the prevalence of anaemia at 12 months of age, and a 23 percent relative decrease.

Treatment with micronutrient powders caused an additional 4.52 deaths per 10,000 children [95 percent CIs = 11.5 to -1.05].

If coverage of the intervention is 100 percent, universal home fortification with micronutrient powders (MNPs) from age 6-12 months averted -165 [95 percent CIs = -546 to 142] DALYs per 10,000 children, compared with a control cohort. Of these averted DALYs, -243 [95 percent CIs = -616 to 56.3] were YLLs and 78.1 [95 percent CIs = 70.2 to 85.4] were YLDs.

When the effects of micronutrient powders on anaemia alone (excluding effects on diarrhoea and malaria) are considered, treatment with micronutrient powders caused a change in YLDs per 10,000 children of 80.3.

Treatment with micronutrient powders caused a net increase/decrease in healthcare costs of USD $10.3 per DALY averted.

If coverage was:
* 100 percent, net DALYs per 10,000 children averted was -165.
* 75 percent, net DALYs per 10,000 children averted was -125.

* 50 percent, net DALYs per 10,000 children averted was -83.2.
* 25 percent, net DALYs per 10,000 children averted was -42.3.

[Because our model predicts that treatment with micronutrient powders increases net DALYs, we did not calculate the effect on healthcare costs when MNPs increase DALYs]

Niger (NER)

--- INPUTS ---

Based on the 2017 Global Burden of Disease analysis, the incidence rates of diarrhoea are: mild = 1.4 events per year per child; moderate = 0.76 per year per child; severe = 0.011 per year per child.

Based on the 2017 Global Burden of Disease analysis, the incidence rates of malaria are: mild = 0.58 per year per child; moderate = 0.31 per year per child; severe = 0.0045 per year per child.

Based on the most recent Demographic and Health Survey (or equivalent), the prevalences of anaemia are:
6-8 months mild 27 percent, moderate 50 percent, severe 4.4 percent
9-11 months mild 22 percent, moderate 57 percent, severe 5.7 percent
12-17 months mild 22 percent, moderate 65 percent, severe 3.1 percent.

Background mortality rates through the treatment period are 4.17 percent per year on average.

Case-specific mortality rates are 0.133 percent for a case of diarrhoea; 0.5 percent for a case of malaria.

Niger is classified as having endemic malaria, and has an Insecticide Treated bednet coverage rate of 20.1 percent based on 2015 UNICEF bednet coverage data.

In this country, the cost of an outpatient clinic visit is $0.598, and of hospitalisation is $1.73. We modelled the cost of MNPs as US $9.9 per course (including implementation).

--- OUTPUTS ---

Treatment with micronutrient powders caused a 5 percent increase in the incidence of diarrhoea, and a 6 percent increase in the incidence of malaria.
Treatment with micronutrient powders produced a 21 percent absolute decrease in the prevalence of anaemia at 12 months of age, and a 24 percent relative decrease.

Treatment with micronutrient powders caused an additional 7.19 deaths per 10,000 children [95 percent CIs = 17.5 to -2.31].

If coverage of the intervention is 100 percent, universal home fortification with micronutrient powders (MNPs) from age 6-12 months averted -360 [95 percent CIs = -999 to 229] DALYs per 10,000 children, compared with a control cohort. Of these averted DALYs, -439 [95 percent CIs = -1.07e+03 to 141] were YLLs and 79.6 [95 percent CIs = 71.6 to 87.8] were YLDs.

When the effects of micronutrient powders on anaemia alone (excluding effects on diarrhoea and malaria) are considered, treatment with micronutrient powders caused a change in YLDs per 10,000 children of 82.1.

Treatment with micronutrient powders caused a net increase/decrease in healthcare costs of USD $10 per DALY averted.

If coverage was:
* 100 percent, net DALYs per 10,000 children averted was -360.
* 75 percent, net DALYs per 10,000 children averted was -271.
* 50 percent, net DALYs per 10,000 children averted was -181.
* 25 percent, net DALYs per 10,000 children averted was -91.5.

[Because our model predicts that treatment with micronutrient powders increases net DALYs, we did not calculate the effect on healthcare costs when MNPs increase DALYs]

Mali (MLI)

--- INPUTS ---

Based on the 2017 Global Burden of Disease analysis, the incidence rates of diarrhoea are: mild = 1.2 events per year per child; moderate = 0.63 per year per child; severe = 0.009 per year per child.

Based on the 2017 Global Burden of Disease analysis, the incidence rates of malaria are: mild = 0.47 per year per child; moderate = 0.25 per year per child; severe = 0.0036 per year per child.

Based on the most recent Demographic and Health Survey (or equivalent), the prevalences of anaemia are:
6-8 months mild 29 percent, moderate 53 percent, severe 6.6 percent
9-11 months mild 21 percent, moderate 58 percent, severe 8.5 percent
12-17 months mild 17 percent, moderate 61 percent, severe 12 percent.

Background mortality rates through the treatment period are 5.34 percent per year on average.

Case-specific mortality rates are 0.0771 percent for a case of diarrhoea; 0.597 percent for a case of malaria.

Mali is classified as having endemic malaria, and has an Insecticide Treated bednet coverage rate of 27 percent based on 2015 UNICEF bednet coverage data.

In this country, the cost of an outpatient clinic visit is $1.11, and of hospitalisation is $3.82. We modelled the cost of MNPs as US $9.9 per course (including implementation).

--- OUTPUTS ---

Treatment with micronutrient powders caused a 5 percent increase in the incidence of diarrhoea, and a 6 percent increase in the incidence of malaria.

Treatment with micronutrient powders produced a 23 percent absolute decrease in the prevalence of anaemia at 12 months of age, and a 25 percent relative decrease.

Treatment with micronutrient powders caused an additional 4.74 deaths per 10,000 children [95 percent CIs = 10.9 to -0.766].

If coverage of the intervention is 100 percent, universal home fortification with micronutrient powders (MNPs) from age 6-12 months averted -184 [95 percent CIs = -545 to 141] DALYs per 10,000 children, compared with a control cohort. Of these averted DALYs, -273 [95 percent CIs = -625 to 44] were YLLs and 88.7 [95 percent CIs = 80.4 to 96.8] were YLDs.

When the effects of micronutrient powders on anaemia alone (excluding effects on diarrhoea and malaria) are considered, treatment with micronutrient powders caused a change in YLDs per 10,000 children of 90.6.

Treatment with micronutrient powders caused a net increase/decrease in healthcare costs of USD $10.1 per DALY averted.

If coverage was:
* 100 percent, net DALYs per 10,000 children averted was -184.
* 75 percent, net DALYs per 10,000 children averted was -139.
* 50 percent, net DALYs per 10,000 children averted was -93.
* 25 percent, net DALYs per 10,000 children averted was -47.5.

[Because our model predicts that treatment with micronutrient powders increases net DALYs, we did not calculate the effect on healthcare costs when MNPs increase DALYs]

Burkina Faso (BFA)

--- INPUTS ---

Based on the 2017 Global Burden of Disease analysis, the incidence rates of diarrhoea are: mild = 1.5 events per year per child; moderate = 0.79 per year per child; severe = 0.011 per year per child.

Based on the 2017 Global Burden of Disease analysis, the incidence rates of malaria are: mild = 0.65 per year per child; moderate = 0.35 per year per child; severe = 0.005 per year per child.

Based on the most recent Demographic and Health Survey (or equivalent), the prevalences of anaemia are:
6-8 months mild 13 percent, moderate 69 percent, severe 11 percent
9-11 months mild 8.1 percent, moderate 63 percent, severe 22 percent
12-17 months mild 11 percent, moderate 64 percent, severe 19 percent.

Background mortality rates through the treatment period are 4.35 percent per year on average.

Case-specific mortality rates are 0.0483 percent for a case of diarrhoea; 0.459 percent for a case of malaria.

Burkina Faso is classified as having endemic malaria, and has an Insecticide Treated bednet coverage rate of 75.3 percent based on 2015 UNICEF bednet coverage data.

In this country, the cost of an outpatient clinic visit is $0.872, and of hospitalisation is $3.08. We modelled the cost of MNPs as US $9.9 per course (including implementation).

--- OUTPUTS ---

Treatment with micronutrient powders caused a 5 percent increase in the incidence of diarrhoea, and a 0.4 percent increase in the incidence of malaria.
Treatment with micronutrient powders produced a 23 percent absolute decrease in the prevalence of anaemia at 12 months of age, and a 24 percent relative decrease.

Treatment with micronutrient powders caused an additional 1.38 deaths per 10,000 children [95 percent CIs = 6.23 to -3.59].

If coverage of the intervention is 100 percent, universal home fortification with micronutrient powders (MNPs) from age 6-12 months averted 30.7 [95 percent CIs = -267 to 334] DALYs per 10,000 children, compared with a control cohort. Of these averted DALYs, -81.8 [95 percent CIs = -369 to 212] were YLLs and 112 [95 percent CIs = 102 to 122] were YLDs.

When the effects of micronutrient powders on anaemia alone (excluding effects on diarrhoea and malaria) are considered, treatment with micronutrient powders caused a change in YLDs per 10,000 children of 115.

Treatment with micronutrient powders caused a net increase/decrease in healthcare costs of USD $10 per DALY averted.

If coverage was:
* 100 percent, net DALYs per 10,000 children averted was 30.7.
* 75 percent, net DALYs per 10,000 children averted was 21.7.
* 50 percent, net DALYs per 10,000 children averted was 14.5.
* 25 percent, net DALYs per 10,000 children averted was 6.27.

If coverage was:
* 100 percent: cost per DALY averted was $3267.
* 75 percent: cost per DALY averted was $4599.
* 50 percent: cost per DALY averted was $6889.
* 25 percent: cost per DALY averted was $15830.

Congo (COG)

--- INPUTS ---

Based on the 2017 Global Burden of Disease analysis, the incidence rates of diarrhoea are: mild = 1.8 events per year per child; moderate = 0.99 per year per child; severe = 0.014 per year per child.

Based on the 2017 Global Burden of Disease analysis, the incidence rates of malaria are: mild = 0.41 per year per child; moderate = 0.22 per year per child; severe = 0.0031 per year per child.

Based on the most recent Demographic and Health Survey (or equivalent), the prevalences of anaemia are:
6-8 months mild 35 percent, moderate 50 percent, severe 0.6 percent
9-11 months mild 27 percent, moderate 60 percent, severe 1 percent
12-17 months mild 31 percent, moderate 48 percent, severe 1.9 percent.

Background mortality rates through the treatment period are 2.34 percent per year on average.

Case-specific mortality rates are 0.0215 percent for a case of diarrhoea; 0.153 percent for a case of malaria.

Congo is classified as having endemic malaria, and has an Insecticide Treated bednet coverage rate of 26 percent based on 2015 UNICEF bednet coverage data.

In this country, the cost of an outpatient clinic visit is $2.16, and of hospitalisation is $11.1. We modelled the cost of MNPs as US $9.9 per course (including implementation).

--- OUTPUTS ---

Treatment with micronutrient powders caused a 5 percent increase in the incidence of diarrhoea, and a 6 percent increase in the incidence of malaria.
Treatment with micronutrient powders produced a 22 percent absolute decrease in the prevalence of anaemia at 12 months of age, and a 24 percent relative decrease.

Treatment with micronutrient powders caused an additional 1.45 deaths per 10,000 children [95 percent CIs = 3.19 to -0.317].

If coverage of the intervention is 100 percent, universal home fortification with micronutrient powders (MNPs) from age 6-12 months averted -33.5 [95 percent CIs = -153 to 87] DALYs per 10,000 children, compared with a control cohort. Of these averted DALYs, -92.6 [95 percent CIs = -204 to 20.3] were YLLs and 59.1 [95 percent CIs = 50.6 to 66.8] were YLDs.

When the effects of micronutrient powders on anaemia alone (excluding effects on diarrhoea and malaria) are considered, treatment with micronutrient powders caused a change in YLDs per 10,000 children of 61.7.

Treatment with micronutrient powders caused a net increase/decrease in healthcare costs of USD $10.3 per DALY averted.

If coverage was:
* 100 percent, net DALYs per 10,000 children averted was -33.5.
* 75 percent, net DALYs per 10,000 children averted was -25.7.
* 50 percent, net DALYs per 10,000 children averted was -17.1.
* 25 percent, net DALYs per 10,000 children averted was -8.93.

[Because our model predicts that treatment with micronutrient powders increases net DALYs, we did not calculate the effect on healthcare costs when MNPs increase DALYs]

Somalia (SOM)

--- INPUTS ---

Based on the 2017 Global Burden of Disease analysis, the incidence rates of diarrhoea are: mild = 1.2 events per year per child; moderate = 0.64 per year per child; severe = 0.0093 per year per child.

Based on the 2017 Global Burden of Disease analysis, the incidence rates of malaria are: mild = 0.05 per year per child; moderate = 0.027 per year per child; severe = 0.00038 per year per child.

Based on the most recent Demographic and Health Survey (or equivalent), the prevalences of anaemia are:
6-8 months mild 28 percent, moderate 41 percent, severe 1.7 percent
9-11 months mild 30 percent, moderate 46 percent, severe 6.5 percent
12-17 months mild 29 percent, moderate 38 percent, severe 4.1 percent.

Background mortality rates through the treatment period are 6.2 percent per year on average.

Case-specific mortality rates are 0.0599 percent for a case of diarrhoea; 0.0947 percent for a case of malaria.

Somalia is classified as having endemic malaria, and has an Insecticide Treated bednet coverage rate of 11 percent based on 2015 UNICEF bednet coverage data.

In this country, the cost of an outpatient clinic visit is $0, and of hospitalisation is $0. We modelled the cost of MNPs as US $9.9 per course (including implementation).

--- OUTPUTS ---

Treatment with micronutrient powders caused a 4 percent increase in the incidence of diarrhoea, and a 7 percent increase in the incidence of malaria.
Treatment with micronutrient powders produced a 22 percent absolute decrease in the prevalence of anaemia at 12 months of age, and a 26 percent relative decrease.

Treatment with micronutrient powders caused an additional 2.05 deaths per 10,000 children [95 percent CIs = 8.57 to -3.36].

If coverage of the intervention is 100 percent, universal home fortification with micronutrient powders (MNPs) from age 6-12 months averted -40.8 [95 percent CIs = -402 to 259] DALYs per 10,000 children, compared with a control cohort. Of these averted DALYs, -111 [95 percent CIs = -465 to 182] were YLLs and 70.3 [95 percent CIs = 63 to 76.8] were YLDs.

When the effects of micronutrient powders on anaemia alone (excluding effects on diarrhoea and malaria) are considered, treatment with

micronutrient powders caused a change in YLDs per 10,000 children of 71.7.

Treatment with micronutrient powders caused a net increase/decrease in healthcare costs of USD $9.9 per DALY averted.

If coverage was:
* 100 percent, net DALYs per 10,000 children averted was -40.8.
* 75 percent, net DALYs per 10,000 children averted was -31.9.
* 50 percent, net DALYs per 10,000 children averted was -21.2.
* 25 percent, net DALYs per 10,000 children averted was -11.5.

[Because our model predicts that treatment with micronutrient powders increases net DALYs, we did not calculate the effect on healthcare costs when MNPs increase DALYs]

Ghana (GHA)

--- INPUTS ---

Based on the 2017 Global Burden of Disease analysis, the incidence rates of diarrhoea are: mild = 1.3 events per year per child; moderate = 0.72 per year per child; severe = 0.01 per year per child.

Based on the 2017 Global Burden of Disease analysis, the incidence rates of malaria are: mild = 0.36 per year per child; moderate = 0.19 per year per child; severe = 0.0028 per year per child.

Based on the most recent Demographic and Health Survey (or equivalent), the prevalences of anaemia are:
6-8 months mild 25 percent, moderate 52 percent, severe 3.4 percent
9-11 months mild 28 percent, moderate 46 percent, severe 4.1 percent
12-17 months mild 24 percent, moderate 48 percent, severe 6 percent.

Background mortality rates through the treatment period are 3.02 percent per year on average.

Case-specific mortality rates are 0.0214 percent for a case of diarrhoea; 0.204 percent for a case of malaria.

Ghana is classified as having endemic malaria, and has an Insecticide Treated bednet coverage rate of 46.6 percent based on 2015 UNICEF bednet coverage data.

In this country, the cost of an outpatient clinic visit is $0.706, and of hospitalisation is $2.61. We modelled the cost of MNPs as US $9.9 per course (including implementation).

--- OUTPUTS ---

Treatment with micronutrient powders caused a 5 percent increase in the incidence of diarrhoea, and a 4 percent increase in the incidence of malaria.
Treatment with micronutrient powders produced a 18 percent absolute decrease in the prevalence of anaemia at 12 months of age, and a 23 percent relative decrease.

Treatment with micronutrient powders caused an additional 0.739 deaths per 10,000 children [95 percent CIs = 2.02 to -0.488].

If coverage of the intervention is 100 percent, universal home fortification with micronutrient powders (MNPs) from age 6-12 months averted 25.4 [95 percent CIs = -61.9 to 109] DALYs per 10,000 children, compared with a control cohort. Of these averted DALYs, -45.6 [95 percent CIs = -124 to 30.1] were YLLs and 70.9 [95 percent CIs = 62.6 to 78.9] were YLDs.

When the effects of micronutrient powders on anaemia alone (excluding effects on diarrhoea and malaria) are considered, treatment with micronutrient powders caused a change in YLDs per 10,000 children of 73.

Treatment with micronutrient powders caused a net increase/decrease in healthcare costs of USD $10 per DALY averted.

If coverage was:
* 100 percent, net DALYs per 10,000 children averted was 25.4.
* 75 percent, net DALYs per 10,000 children averted was 18.7.
* 50 percent, net DALYs per 10,000 children averted was 12.4.
* 25 percent, net DALYs per 10,000 children averted was 5.85.

If coverage was:
* 100 percent: cost per DALY averted was $3945.
* 75 percent: cost per DALY averted was $5343.
* 50 percent: cost per DALY averted was $8039.
* 25 percent: cost per DALY averted was $16969.

Togo (TGO)

--- INPUTS ---

Based on the 2017 Global Burden of Disease analysis, the incidence rates of diarrhoea are: mild = 1.7 events per year per child; moderate = 0.91 per year per child; severe = 0.013 per year per child.

Based on the 2017 Global Burden of Disease analysis, the incidence rates of malaria are: mild = 0.79 per year per child; moderate = 0.42 per year per child; severe = 0.0061 per year per child.

Based on the most recent Demographic and Health Survey (or equivalent), the prevalences of anaemia are:
6-8 months mild 26 percent, moderate 62 percent, severe 1.9 percent
9-11 months mild 23 percent, moderate 59 percent, severe 3.9 percent
12-17 months mild 23 percent, moderate 57 percent, severe 4.6 percent.

Background mortality rates through the treatment period are 3.75 percent per year on average.

Case-specific mortality rates are 0.0309 percent for a case of diarrhoea; 0.199 percent for a case of malaria.

Togo is classified as having endemic malaria, and has an Insecticide Treated bednet coverage rate of 42.8 percent based on 2015 UNICEF bednet coverage data.

In this country, the cost of an outpatient clinic visit is $1.06, and of hospitalisation is $3.71. We modelled the cost of MNPs as US $9.9 per course (including implementation).

--- OUTPUTS ---

Treatment with micronutrient powders caused a 5 percent increase in the incidence of diarrhoea, and a 4 percent increase in the incidence of malaria.
Treatment with micronutrient powders produced a 24 percent absolute decrease in the prevalence of anaemia at 12 months of age, and a 26 percent relative decrease.

Treatment with micronutrient powders caused an additional 2.62 deaths per 10,000 children [95 percent CIs = 6.06 to -0.74].

If coverage of the intervention is 100 percent, universal home fortification with micronutrient powders (MNPs) from age 6-12 months averted -83.7 [95 percent CIs = -296 to 123] DALYs per 10,000 children, compared with a control cohort. Of these averted DALYs, -155 [95 percent CIs = -358 to 43.9] were YLLs and 71.3 [95 percent CIs = 62.4 to 79] were YLDs.

When the effects of micronutrient powders on anaemia alone (excluding effects on diarrhoea and malaria) are considered, treatment with micronutrient powders caused a change in YLDs per 10,000 children of 73.9.

Treatment with micronutrient powders caused a net increase/decrease in healthcare costs of USD $10.1 per DALY averted.

If coverage was:
* 100 percent, net DALYs per 10,000 children averted was -83.7.
* 75 percent, net DALYs per 10,000 children averted was -63.4.
* 50 percent, net DALYs per 10,000 children averted was -42.3.
* 25 percent, net DALYs per 10,000 children averted was -21.6.

[Because our model predicts that treatment with micronutrient powders increases net DALYs, we did not calculate the effect on healthcare costs when MNPs increase DALYs]

Benin (BEN)

--- INPUTS ---

Based on the 2017 Global Burden of Disease analysis, the incidence rates of diarrhoea are: mild = 0.84 events per year per child; moderate = 0.45 per year per child; severe = 0.0065 per year per child.

Based on the 2017 Global Burden of Disease analysis, the incidence rates of malaria are: mild = 0.64 per year per child; moderate = 0.34 per year per child; severe = 0.005 per year per child.

Based on the most recent Demographic and Health Survey (or equivalent), the prevalences of anaemia are:
6-8 months mild 25 percent, moderate 33 percent, severe 5.5 percent
9-11 months mild 28 percent, moderate 41 percent, severe 3.4 percent
12-17 months mild 29 percent, moderate 36 percent, severe 2.9 percent.

Background mortality rates through the treatment period are 4.62 percent per year on average.

Case-specific mortality rates are 0.0868 percent for a case of diarrhoea; 0.23 percent for a case of malaria.

Benin is classified as having endemic malaria, and has an Insecticide Treated bednet coverage rate of 73 percent based on 2015 UNICEF bednet coverage data.

In this country, the cost of an outpatient clinic visit is $1.52, and of hospitalisation is $5.73. We modelled the cost of MNPs as US $9.9 per course (including implementation).

--- OUTPUTS ---

Treatment with micronutrient powders caused a 4 percent increase in the incidence of diarrhoea, and a 0.8 percent increase in the incidence of malaria.
Treatment with micronutrient powders produced a 20 percent absolute decrease in the prevalence of anaemia at 12 months of age, and a 27 percent relative decrease.

Treatment with micronutrient powders caused an additional 0.676 deaths per 10,000 children [95 percent CIs = 3.41 to -1.9].

If coverage of the intervention is 100 percent, universal home fortification with micronutrient powders (MNPs) from age 6-12 months averted 24.5 [95 percent CIs = -144 to 184] DALYs per 10,000 children, compared with a control cohort. Of these averted DALYs, -40.1 [95 percent CIs = -202 to 113] were YLLs and 64.5 [95 percent CIs = 57.8 to 71.1] were YLDs.

When the effects of micronutrient powders on anaemia alone (excluding effects on diarrhoea and malaria) are considered, treatment with micronutrient powders caused a change in YLDs per 10,000 children of 65.7.

Treatment with micronutrient powders caused a net increase/decrease in healthcare costs of USD $10 per DALY averted.

If coverage was:
* 100 percent, net DALYs per 10,000 children averted was 24.5.
* 75 percent, net DALYs per 10,000 children averted was 17.6.
* 50 percent, net DALYs per 10,000 children averted was 11.8.
* 25 percent, net DALYs per 10,000 children averted was 5.32.

If coverage was:
* 100 percent: cost per DALY averted was $4092.
* 75 percent: cost per DALY averted was $5687.
* 50 percent: cost per DALY averted was $8463.

* 25 percent: cost per DALY averted was $18667.

Cote d'Ivoire (CIV)

--- INPUTS ---

Based on the 2017 Global Burden of Disease analysis, the incidence rates of diarrhoea are: mild = 1.8 events per year per child; moderate = 0.97 per year per child; severe = 0.014 per year per child.

Based on the 2017 Global Burden of Disease analysis, the incidence rates of malaria are: mild = 0.26 per year per child; moderate = 0.14 per year per child; severe = 0.002 per year per child.

Based on the most recent Demographic and Health Survey (or equivalent), the prevalences of anaemia are:
6-8 months mild 15 percent, moderate 62 percent, severe 10 percent
9-11 months mild 24 percent, moderate 64 percent, severe 4.5 percent
12-17 months mild 23 percent, moderate 55 percent, severe 3.8 percent.

Background mortality rates through the treatment period are 4.7 percent per year on average.

Case-specific mortality rates are 0.0401 percent for a case of diarrhoea; 0.388 percent for a case of malaria.

Cote d'Ivoire is classified as having endemic malaria, and has an Insecticide Treated bednet coverage rate of 37.2 percent based on 2015 UNICEF bednet coverage data.

In this country, the cost of an outpatient clinic visit is $1.84, and of hospitalisation is $7.18. We modelled the cost of MNPs as US $9.9 per course (including implementation).

--- OUTPUTS ---

Treatment with micronutrient powders caused a 5 percent increase in the incidence of diarrhoea, and a 5 percent increase in the incidence of malaria.
Treatment with micronutrient powders produced a 22 percent absolute decrease in the prevalence of anaemia at 12 months of age, and a 23 percent relative decrease.

Treatment with micronutrient powders caused an additional 1.48 deaths per 10,000 children [95 percent CIs = 3.82 to -0.832].

If coverage of the intervention is 100 percent, universal home fortification with micronutrient powders (MNPs) from age 6-12 months averted -7.22 [95 percent CIs = -141 to 123] DALYs per 10,000 children, compared with a control cohort. Of these averted DALYs, -77.7 [95 percent CIs = -201 to 43.7] were YLLs and 70.4 [95 percent CIs = 60.1 to 78.8] were YLDs.

When the effects of micronutrient powders on anaemia alone (excluding effects on diarrhoea and malaria) are considered, treatment with

micronutrient powders caused a change in YLDs per 10,000 children of 73.2.

Treatment with micronutrient powders caused a net increase/decrease in healthcare costs of USD $10.2 per DALY averted.

If coverage was:
* 100 percent, net DALYs per 10,000 children averted was -7.22.
* 75 percent, net DALYs per 10,000 children averted was -6.11.
* 50 percent, net DALYs per 10,000 children averted was -4.01.
* 25 percent, net DALYs per 10,000 children averted was -2.39.

[Because our model predicts that treatment with micronutrient powders increases net DALYs, we did not calculate the effect on healthcare costs when MNPs increase DALYs]

Liberia (LBR)

--- INPUTS ---

Based on the 2017 Global Burden of Disease analysis, the incidence rates of diarrhoea are: mild = 2.3 events per year per child; moderate = 1.2 per year per child; severe = 0.017 per year per child.

Based on the 2017 Global Burden of Disease analysis, the incidence rates of malaria are: mild = 0.43 per year per child; moderate = 0.23 per year per child; severe = 0.0033 per year per child.

Based on the most recent Demographic and Health Survey (or equivalent), the prevalences of anaemia are:
6-8 months mild 34 percent, moderate 28 percent, severe 1.5 percent
9-11 months mild 33 percent, moderate 25 percent, severe 0.43 percent
12-17 months mild 32 percent, moderate 27 percent, severe 1.3 percent.

Background mortality rates through the treatment period are 3.7 percent per year on average.

Case-specific mortality rates are 0.0267 percent for a case of diarrhoea; 0.196 percent for a case of malaria.

Liberia is classified as having endemic malaria, and has an Insecticide Treated bednet coverage rate of 38.1 percent based on 2015 UNICEF bednet coverage data.

In this country, the cost of an outpatient clinic visit is $0.616, and of hospitalisation is $1.49. We modelled the cost of MNPs as US $9.9 per course (including implementation).

--- OUTPUTS ---

Treatment with micronutrient powders caused a 5 percent increase in the incidence of diarrhoea, and a 5 percent increase in the incidence of malaria.
Treatment with micronutrient powders produced a 17 percent absolute decrease in the prevalence of anaemia at 12 months of age, and a 29 percent relative decrease.

Treatment with micronutrient powders caused an additional 1.38 deaths per 10,000 children [95 percent CIs = 4.1 to -0.764].

If coverage of the intervention is 100 percent, universal home fortification with micronutrient powders (MNPs) from age 6-12 months averted -43.7 [95 percent CIs = -220 to 95.1] DALYs per 10,000 children, compared with a control cohort. Of these averted DALYs, -83.5 [95 percent CIs = -249 to 46.3] were YLLs and 39.8 [95 percent CIs = 28.9 to 48.7] were YLDs.

When the effects of micronutrient powders on anaemia alone (excluding effects on diarrhoea and malaria) are considered, treatment with micronutrient powders caused a change in YLDs per 10,000 children of 42.9.

Treatment with micronutrient powders caused a net increase/decrease in healthcare costs of USD $10 per DALY averted.

If coverage was:
* 100 percent, net DALYs per 10,000 children averted was -43.7.
* 75 percent, net DALYs per 10,000 children averted was -33.3.
* 50 percent, net DALYs per 10,000 children averted was -22.2.
* 25 percent, net DALYs per 10,000 children averted was -11.4.

[Because our model predicts that treatment with micronutrient powders increases net DALYs, we did not calculate the effect on healthcare costs when MNPs increase DALYs]

Sierra Leone (SLE)

--- INPUTS ---

Based on the 2017 Global Burden of Disease analysis, the incidence rates of diarrhoea are: mild = 1.2 events per year per child; moderate = 0.66 per year per child; severe = 0.0095 per year per child.

Based on the 2017 Global Burden of Disease analysis, the incidence rates of malaria are: mild = 0.77 per year per child; moderate = 0.41 per year per child; severe = 0.0059 per year per child.

Based on the most recent Demographic and Health Survey (or equivalent), the prevalences of anaemia are:
6-8 months mild 27 percent, moderate 53 percent, severe 7.1 percent
9-11 months mild 22 percent, moderate 55 percent, severe 7 percent
12-17 months mild 27 percent, moderate 49 percent, severe 7 percent.

Background mortality rates through the treatment period are 6.32 percent per year on average.

Case-specific mortality rates are 0.0502 percent for a case of diarrhoea; 0.379 percent for a case of malaria.

Sierra Leone is classified as having endemic malaria, and has an Insecticide Treated bednet coverage rate of 49 percent based on 2015 UNICEF bednet coverage data.

In this country, the cost of an outpatient clinic visit is $0.946, and of hospitalisation is $2.87. We modelled the cost of MNPs as US $9.9 per course (including implementation).

--- OUTPUTS ---

Treatment with micronutrient powders caused a 5 percent increase in the incidence of diarrhoea, and a 4 percent increase in the incidence of malaria.
Treatment with micronutrient powders produced a 22 percent absolute decrease in the prevalence of anaemia at 12 months of age, and a 25 percent relative decrease.

Treatment with micronutrient powders caused an additional 3.24 deaths per 10,000 children [95 percent CIs = 7.86 to -1.35].

If coverage of the intervention is 100 percent, universal home fortification with micronutrient powders (MNPs) from age 6-12 months averted -87.9 [95 percent CIs = -324 to 147] DALYs per 10,000 children, compared with a control cohort. Of these averted DALYs, -160 [95 percent CIs = -388 to 66.4] were YLLs and 72.1 [95 percent CIs = 63.4 to 80.3] were YLDs.

When the effects of micronutrient powders on anaemia alone (excluding effects on diarrhoea and malaria) are considered, treatment with micronutrient powders caused a change in YLDs per 10,000 children of 74.1.

Treatment with micronutrient powders caused a net increase/decrease in healthcare costs of USD $10 per DALY averted.

If coverage was:
* 100 percent, net DALYs per 10,000 children averted was -87.9.
* 75 percent, net DALYs per 10,000 children averted was -66.9.
* 50 percent, net DALYs per 10,000 children averted was -44.6.
* 25 percent, net DALYs per 10,000 children averted was -22.9.

[Because our model predicts that treatment with micronutrient powders increases net DALYs, we did not calculate the effect on healthcare costs when MNPs increase DALYs]

Guinea (GIN)

--- INPUTS ---

Based on the 2017 Global Burden of Disease analysis, the incidence rates of diarrhoea are: mild = 1.6 events per year per child; moderate = 0.87 per year per child; severe = 0.013 per year per child.

Based on the 2017 Global Burden of Disease analysis, the incidence rates of malaria are: mild = 0.6 per year per child; moderate = 0.32 per year per child; severe = 0.0046 per year per child.

Based on the most recent Demographic and Health Survey (or equivalent), the prevalences of anaemia are:
6-8 months mild 27 percent, moderate 48 percent, severe 8.9 percent
9-11 months mild 17 percent, moderate 59 percent, severe 11 percent

12-17 months mild 21 percent, moderate 54 percent, severe 10 percent.

Background mortality rates through the treatment period are 4.41 percent per year on average.

Case-specific mortality rates are 0.0326 percent for a case of diarrhoea; 0.297 percent for a case of malaria.

Guinea is classified as having endemic malaria, and has an Insecticide Treated bednet coverage rate of 26 percent based on 2015 UNICEF bednet coverage data.

In this country, the cost of an outpatient clinic visit is $0.917, and of hospitalisation is $3.08. We modelled the cost of MNPs as US $9.9 per course (including implementation).

--- OUTPUTS ---

Treatment with micronutrient powders caused a 5 percent increase in the incidence of diarrhoea, and a 6 percent increase in the incidence of malaria.
Treatment with micronutrient powders produced a 21 percent absolute decrease in the prevalence of anaemia at 12 months of age, and a 24 percent relative decrease.

Treatment with micronutrient powders caused an additional 4.04 deaths per 10,000 children [95 percent CIs = 9.04 to -0.526].

If coverage of the intervention is 100 percent, universal home fortification with micronutrient powders (MNPs) from age 6-12 months averted -150 [95 percent CIs = -451 to 126] DALYs per 10,000 children, compared with a control cohort. Of these averted DALYs, -236 [95 percent CIs = -527 to 30.7] were YLLs and 85.6 [95 percent CIs = 75.8 to 95.3] were YLDs.

When the effects of micronutrient powders on anaemia alone (excluding effects on diarrhoea and malaria) are considered, treatment with micronutrient powders caused a change in YLDs per 10,000 children of 88.3.

Treatment with micronutrient powders caused a net increase/decrease in healthcare costs of USD $10.1 per DALY averted.

If coverage was:
* 100 percent, net DALYs per 10,000 children averted was -150.
* 75 percent, net DALYs per 10,000 children averted was -113.
* 50 percent, net DALYs per 10,000 children averted was -75.7.
* 25 percent, net DALYs per 10,000 children averted was -38.5.

[Because our model predicts that treatment with micronutrient powders increases net DALYs, we did not calculate the effect on healthcare costs when MNPs increase DALYs]

Senegal (SEN)

--- INPUTS ---

Based on the 2017 Global Burden of Disease analysis, the incidence rates of diarrhoea are: mild = 1.8 events per year per child; moderate = 0.99 per year per child; severe = 0.014 per year per child.

Based on the 2017 Global Burden of Disease analysis, the incidence rates of malaria are: mild = 0.055 per year per child; moderate = 0.03 per year per child; severe = 0.00043 per year per child.

Based on the most recent Demographic and Health Survey (or equivalent), the prevalences of anaemia are:
6-8 months mild 32 percent, moderate 44 percent, severe 1.5 percent
9-11 months mild 20 percent, moderate 52 percent, severe 6.1 percent
12-17 months mild 21 percent, moderate 56 percent, severe 5.7 percent.

Background mortality rates through the treatment period are 2.91 percent per year on average.

Case-specific mortality rates are 0.031 percent for a case of diarrhoea; 0.248 percent for a case of malaria.

Senegal is classified as having endemic malaria, and has an Insecticide Treated bednet coverage rate of 43.2 percent based on 2015 UNICEF bednet coverage data.

In this country, the cost of an outpatient clinic visit is $1.51, and of hospitalisation is $6.06. We modelled the cost of MNPs as US $9.9 per course (including implementation).

--- OUTPUTS ---

Treatment with micronutrient powders caused a 4 percent increase in the incidence of diarrhoea, and a 5 percent increase in the incidence of malaria.
Treatment with micronutrient powders produced a 20 percent absolute decrease in the prevalence of anaemia at 12 months of age, and a 25 percent relative decrease.

Treatment with micronutrient powders caused an additional 1.17 deaths per 10,000 children [95 percent CIs = 5.43 to -1.99].

If coverage of the intervention is 100 percent, universal home fortification with micronutrient powders (MNPs) from age 6-12 months averted 2.68 [95 percent CIs = -288 to 219] DALYs per 10,000 children, compared with a control cohort. Of these averted DALYs, -76.7 [95 percent CIs = -358 to 131] were YLLs and 79.4 [95 percent CIs = 70.3 to 87.7] were YLDs.

When the effects of micronutrient powders on anaemia alone (excluding effects on diarrhoea and malaria) are considered, treatment with micronutrient powders caused a change in YLDs per 10,000 children of 81.8.

Treatment with micronutrient powders caused a net increase/decrease in healthcare costs of USD $10.1 per DALY averted.

If coverage was:
* 100 percent, net DALYs per 10,000 children averted was 2.68.
* 75 percent, net DALYs per 10,000 children averted was 1.18.

* 50 percent, net DALYs per 10,000 children averted was 0.714.
* 25 percent, net DALYs per 10,000 children averted was -0.284.

If coverage was:
* 100 percent: cost per DALY averted was $37817.
* 75 percent: cost per DALY averted was $85349.
* 50 percent: cost per DALY averted was $140223.
* 25 percent: cost per DALY averted was $-351189.

Gambia (GMB)

--- INPUTS ---

Based on the 2017 Global Burden of Disease analysis, the incidence rates of diarrhoea are: mild = 1.6 events per year per child; moderate = 0.85 per year per child; severe = 0.012 per year per child.

Based on the 2017 Global Burden of Disease analysis, the incidence rates of malaria are: mild = 0.024 per year per child; moderate = 0.013 per year per child; severe = 0.00019 per year per child.

Based on the most recent Demographic and Health Survey (or equivalent), the prevalences of anaemia are:
6-8 months mild 29 percent, moderate 52 percent, severe 1.6 percent
9-11 months mild 25 percent, moderate 62 percent, severe 3.7 percent
12-17 months mild 24 percent, moderate 54 percent, severe 7 percent.

Background mortality rates through the treatment period are 3.41 percent per year on average.

Case-specific mortality rates are 0.0252 percent for a case of diarrhoea; 0.256 percent for a case of malaria.

Gambia is classified as having endemic malaria, and has an Insecticide Treated bednet coverage rate of 47 percent based on 2015 UNICEF bednet coverage data.

In this country, the cost of an outpatient clinic visit is $0.552, and of hospitalisation is $2.01. We modelled the cost of MNPs as US $9.9 per course (including implementation).

--- OUTPUTS ---

Treatment with micronutrient powders caused a 5 percent increase in the incidence of diarrhoea, and a 3 percent increase in the incidence of malaria.
Treatment with micronutrient powders produced a 21 percent absolute decrease in the prevalence of anaemia at 12 months of age, and a 23 percent relative decrease.

Treatment with micronutrient powders caused an additional 0.578 deaths per 10,000 children [95 percent CIs = 2.46 to -1.02].

If coverage of the intervention is 100 percent, universal home fortification with micronutrient powders (MNPs) from age 6-12 months

averted 44.5 [95 percent CIs = -77.1 to 149] DALYs per 10,000 children, compared with a control cohort. Of these averted DALYs, -34.9 [95 percent CIs = -149 to 61.7] were YLLs and 79.4 [95 percent CIs = 71.5 to 87.5] were YLDs.

When the effects of micronutrient powders on anaemia alone (excluding effects on diarrhoea and malaria) are considered, treatment with micronutrient powders caused a change in YLDs per 10,000 children of 81.6.

Treatment with micronutrient powders caused a net increase/decrease in healthcare costs of USD $9.98 per DALY averted.

If coverage was:
* 100 percent, net DALYs per 10,000 children averted was 44.5.
* 75 percent, net DALYs per 10,000 children averted was 32.9.
* 50 percent, net DALYs per 10,000 children averted was 21.9.
* 25 percent, net DALYs per 10,000 children averted was 10.6.

If coverage was:
* 100 percent: cost per DALY averted was $2240.
* 75 percent: cost per DALY averted was $3025.
* 50 percent: cost per DALY averted was $4540.
* 25 percent: cost per DALY averted was $9402.

Cape Verde (CPV)

--- INPUTS ---

Based on the 2017 Global Burden of Disease analysis, the incidence rates of diarrhoea are: mild = 1.9 events per year per child; moderate = 1 per year per child; severe = 0.014 per year per child.

Based on the 2017 Global Burden of Disease analysis, the incidence rates of malaria are: mild = 0 per year per child; moderate = 0 per year per child; severe = 0 per year per child.

Based on the most recent Demographic and Health Survey (or equivalent), the prevalences of anaemia are:
6-8 months mild 28 percent, moderate 35 percent, severe 0 percent
9-11 months mild 26 percent, moderate 32 percent, severe 6.2 percent
12-17 months mild 28 percent, moderate 37 percent, severe 2.2 percent.

Background mortality rates through the treatment period are 1.43 percent per year on average.

Case-specific mortality rates are 0.00373 percent for a case of diarrhoea; 0 percent for a case of malaria.

Cape Verde is not classified as having endemic malaria, and has an Insecticide Treated bednet coverage rate of 0 percent based on 2015 UNICEF bednet coverage data.

In this country, the cost of an outpatient clinic visit is $4.48, and of hospitalisation is $22.7. We modelled the cost of MNPs as US $9.9 per course (including implementation).

--- OUTPUTS ---

Treatment with micronutrient powders caused a 5 percent increase in the incidence of diarrhoea, and a NaN percent decrease in the incidence of malaria.
Treatment with micronutrient powders produced a 13 percent absolute decrease in the prevalence of anaemia at 12 months of age, and a 21 percent relative decrease.

Treatment with micronutrient powders caused an additional 0.0924 deaths per 10,000 children [95 percent CIs = 0.423 to -0.162].

If coverage of the intervention is 100 percent, universal home fortification with micronutrient powders (MNPs) from age 6-12 months averted 41 [95 percent CIs = 5.99 to 69.1] DALYs per 10,000 children, compared with a control cohort. Of these averted DALYs, -6.7 [95 percent CIs = -30.7 to 11.8] were YLLs and 47.7 [95 percent CIs = 36.7 to 57.3] were YLDs.

When the effects of micronutrient powders on anaemia alone (excluding effects on diarrhoea and malaria) are considered, treatment with micronutrient powders caused a change in YLDs per 10,000 children of 50.6.

Treatment with micronutrient powders caused a net increase/decrease in healthcare costs of USD $10.8 per DALY averted.

If coverage was:
* 100 percent, net DALYs per 10,000 children averted was 41.
* 75 percent, net DALYs per 10,000 children averted was 30.8.
* 50 percent, net DALYs per 10,000 children averted was 20.5.
* 25 percent, net DALYs per 10,000 children averted was 10.3.

If coverage was:
* 100 percent: cost per DALY averted was $2632.
* 75 percent: cost per DALY averted was $3437.
* 50 percent: cost per DALY averted was $5066.
* 25 percent: cost per DALY averted was $9859.

Guinea-Bissau (GNB)

--- INPUTS ---

Based on the 2017 Global Burden of Disease analysis, the incidence rates of diarrhoea are: mild = 1.2 events per year per child; moderate = 0.64 per year per child; severe = 0.0092 per year per child.

Based on the 2017 Global Burden of Disease analysis, the incidence rates of malaria are: mild = 0.062 per year per child; moderate = 0.033 per year per child; severe = 0.00048 per year per child.

Based on the most recent Demographic and Health Survey (or equivalent), the prevalences of anaemia are:
6-8 months mild 25 percent, moderate 45 percent, severe 8.3 percent
9-11 months mild 16 percent, moderate 55 percent, severe 10 percent
12-17 months mild 20 percent, moderate 50 percent, severe 9.3 percent.

Background mortality rates through the treatment period are 4.32 percent per year on average.

Case-specific mortality rates are 0.0823 percent for a case of diarrhoea; 0.227 percent for a case of malaria.

Guinea-Bissau is classified as having endemic malaria, and has an Insecticide Treated bednet coverage rate of 80.6 percent based on 2015 UNICEF bednet coverage data.

In this country, the cost of an outpatient clinic visit is $0.498, and of hospitalisation is $1.34. We modelled the cost of MNPs as US $9.9 per course (including implementation).

--- OUTPUTS ---

Treatment with micronutrient powders caused a 6 percent increase in the incidence of diarrhoea, and a 0.7 percent decrease in the incidence of malaria.
Treatment with micronutrient powders produced a 20 percent absolute decrease in the prevalence of anaemia at 12 months of age, and a 24 percent relative decrease.

Treatment with micronutrient powders caused an additional 2.03 deaths per 10,000 children [95 percent CIs = 8.41 to -3.29].

If coverage of the intervention is 100 percent, universal home fortification with micronutrient powders (MNPs) from age 6-12 months averted -28.9 [95 percent CIs = -408 to 289] DALYs per 10,000 children, compared with a control cohort. Of these averted DALYs, -118 [95 percent CIs = -489 to 192] were YLLs and 89.3 [95 percent CIs = 80.9 to 97.8] were YLDs.

When the effects of micronutrient powders on anaemia alone (excluding effects on diarrhoea and malaria) are considered, treatment with micronutrient powders caused a change in YLDs per 10,000 children of 91.2.

Treatment with micronutrient powders caused a net increase/decrease in healthcare costs of USD $9.96 per DALY averted.

If coverage was:
* 100 percent, net DALYs per 10,000 children averted was -28.9.
* 75 percent, net DALYs per 10,000 children averted was -23.7.
* 50 percent, net DALYs per 10,000 children averted was -15.8.
* 25 percent, net DALYs per 10,000 children averted was -9.22.

[Because our model predicts that treatment with micronutrient powders increases net DALYs, we did not calculate the effect on healthcare costs when MNPs increase DALYs]

Mauritania (MRT)

--- INPUTS ---

Based on the 2017 Global Burden of Disease analysis, the incidence rates of diarrhoea are: mild = 1.3 events per year per child; moderate = 0.7 per year per child; severe = 0.01 per year per child.

Based on the 2017 Global Burden of Disease analysis, the incidence rates of malaria are: mild = 0.059 per year per child; moderate = 0.032 per year per child; severe = 0.00045 per year per child.

Based on the most recent Demographic and Health Survey (or equivalent), the prevalences of anaemia are:
6-8 months mild 29 percent, moderate 53 percent, severe 6.6 percent
9-11 months mild 21 percent, moderate 58 percent, severe 8.5 percent
12-17 months mild 17 percent, moderate 61 percent, severe 12 percent.

Background mortality rates through the treatment period are 4.63 percent per year on average.

Case-specific mortality rates are 0.0269 percent for a case of diarrhoea; 0.105 percent for a case of malaria.

Mauritania is classified as having endemic malaria, and has an Insecticide Treated bednet coverage rate of 18 percent based on 2015 UNICEF bednet coverage data.

In this country, the cost of an outpatient clinic visit is $1.83, and of hospitalisation is $7.5. We modelled the cost of MNPs as US $9.9 per course (including implementation).

--- OUTPUTS ---

Treatment with micronutrient powders caused a 5 percent increase in the incidence of diarrhoea, and a 6 percent increase in the incidence of malaria.
Treatment with micronutrient powders produced a 23 percent absolute decrease in the prevalence of anaemia at 12 months of age, and a 25 percent relative decrease.

Treatment with micronutrient powders caused an additional 0.778 deaths per 10,000 children [95 percent CIs = 2.68 to -0.913].

If coverage of the intervention is 100 percent, universal home fortification with micronutrient powders (MNPs) from age 6-12 months averted 40 [95 percent CIs = -87.3 to 153] DALYs per 10,000 children, compared with a control cohort. Of these averted DALYs, -48.5 [95 percent CIs = -167 to 56.9] were YLLs and 88.4 [95 percent CIs = 79.6 to 95.8] were YLDs.

When the effects of micronutrient powders on anaemia alone (excluding effects on diarrhoea and malaria) are considered, treatment with micronutrient powders caused a change in YLDs per 10,000 children of 90.3.

Treatment with micronutrient powders caused a net increase/decrease in healthcare costs of USD $10.1 per DALY averted.

If coverage was:
* 100 percent, net DALYs per 10,000 children averted was 40.
* 75 percent, net DALYs per 10,000 children averted was 29.2.
* 50 percent, net DALYs per 10,000 children averted was 19.3.
* 25 percent, net DALYs per 10,000 children averted was 9.21.

If coverage was:
* 100 percent: cost per DALY averted was $2531.
* 75 percent: cost per DALY averted was $3443.
* 50 percent: cost per DALY averted was $5175.
* 25 percent: cost per DALY averted was $10805.

Rwanda (RWA)

--- INPUTS ---

Based on the 2017 Global Burden of Disease analysis, the incidence rates of diarrhoea are: mild = 1.2 events per year per child; moderate = 0.63 per year per child; severe = 0.009 per year per child.

Based on the 2017 Global Burden of Disease analysis, the incidence rates of malaria are: mild = 0.081 per year per child; moderate = 0.043 per year per child; severe = 0.00063 per year per child.

Based on the most recent Demographic and Health Survey (or equivalent), the prevalences of anaemia are:
6-8 months mild 33 percent, moderate 34 percent, severe 4.1 percent
9-11 months mild 25 percent, moderate 36 percent, severe 0.2 percent
12-17 months mild 31 percent, moderate 21 percent, severe 0.9 percent.

Background mortality rates through the treatment period are 2.17 percent per year on average.

Case-specific mortality rates are 0.028 percent for a case of diarrhoea; 0.309 percent for a case of malaria.

Rwanda is classified as having endemic malaria, and has an Insecticide Treated bednet coverage rate of 68 percent based on 2015 UNICEF bednet coverage data.

In this country, the cost of an outpatient clinic visit is $0.846, and of hospitalisation is $2.85. We modelled the cost of MNPs as US $9.9 per course (including implementation).

--- OUTPUTS ---

Treatment with micronutrient powders caused a 5 percent increase in the incidence of diarrhoea, and a 1 percent increase in the incidence of malaria.
Treatment with micronutrient powders produced a 16 percent absolute decrease in the prevalence of anaemia at 12 months of age, and a 26 percent relative decrease.

Treatment with micronutrient powders caused an additional 0.491 deaths per 10,000 children [95 percent CIs = 2.14 to -0.951].

If coverage of the intervention is 100 percent, universal home fortification with micronutrient powders (MNPs) from age 6-12 months averted 8.3 [95 percent CIs = -106 to 109] DALYs per 10,000 children, compared with a control cohort. Of these averted DALYs, -32.1 [95 percent CIs = -140 to 62.2] were YLLs and 40.3 [95 percent CIs = 33.9 to 47] were YLDs.

When the effects of micronutrient powders on anaemia alone (excluding effects on diarrhoea and malaria) are considered, treatment with micronutrient powders caused a change in YLDs per 10,000 children of 41.9.

Treatment with micronutrient powders caused a net increase/decrease in healthcare costs of USD $9.99 per DALY averted.

If coverage was:
* 100 percent, net DALYs per 10,000 children averted was 8.3.
* 75 percent, net DALYs per 10,000 children averted was 5.24.
* 50 percent, net DALYs per 10,000 children averted was 3.47.
* 25 percent, net DALYs per 10,000 children averted was 1.1.

If coverage was:
* 100 percent: cost per DALY averted was $12036.
* 75 percent: cost per DALY averted was $19010.
* 50 percent: cost per DALY averted was $28640.
* 25 percent: cost per DALY averted was $90003.

Burundi (BDI)

--- INPUTS ---

Based on the 2017 Global Burden of Disease analysis, the incidence rates of diarrhoea are: mild = 1.3 events per year per child; moderate = 0.68 per year per child; severe = 0.0098 per year per child.

Based on the 2017 Global Burden of Disease analysis, the incidence rates of malaria are: mild = 0.4 per year per child; moderate = 0.21 per year per child; severe = 0.0031 per year per child.

Based on the most recent Demographic and Health Survey (or equivalent), the prevalences of anaemia are:
6-8 months mild 26 percent, moderate 46 percent, severe 1.3 percent
9-11 months mild 31 percent, moderate 34 percent, severe 1.5 percent
12-17 months mild 28 percent, moderate 26 percent, severe 1.6 percent.

Background mortality rates through the treatment period are 3.87 percent per year on average.

Case-specific mortality rates are 0.051 percent for a case of diarrhoea; 0.285 percent for a case of malaria.

Burundi is classified as having endemic malaria, and has an Insecticide Treated bednet coverage rate of 53.8 percent based on 2015 UNICEF bednet coverage data.

In this country, the cost of an outpatient clinic visit is $0.405, and of hospitalisation is $0.977. We modelled the cost of MNPs as US $9.9 per course (including implementation).

--- OUTPUTS ---

Treatment with micronutrient powders caused a 4 percent increase in the incidence of diarrhoea, and a 3 percent increase in the incidence of malaria.
Treatment with micronutrient powders produced a 15 percent absolute decrease in the prevalence of anaemia at 12 months of age, and a 23 percent relative decrease.

Treatment with micronutrient powders caused an additional 1.41 deaths per 10,000 children [95 percent CIs = 4.54 to -1.46].

If coverage of the intervention is 100 percent, universal home fortification with micronutrient powders (MNPs) from age 6-12 months averted -40.1 [95 percent CIs = -231 to 135] DALYs per 10,000 children, compared with a control cohort. Of these averted DALYs, -83 [95 percent CIs = -267 to 85.7] were YLLs and 43 [95 percent CIs = 35.9 to 49.3] were YLDs.

When the effects of micronutrient powders on anaemia alone (excluding effects on diarrhoea and malaria) are considered, treatment with micronutrient powders caused a change in YLDs per 10,000 children of 44.6.

Treatment with micronutrient powders caused a net increase/decrease in healthcare costs of USD $9.94 per DALY averted.

If coverage was:
* 100 percent, net DALYs per 10,000 children averted was -40.1.
* 75 percent, net DALYs per 10,000 children averted was -30.8.
* 50 percent, net DALYs per 10,000 children averted was -20.5.
* 25 percent, net DALYs per 10,000 children averted was -10.7.

[Because our model predicts that treatment with micronutrient powders increases net DALYs, we did not calculate the effect on healthcare costs when MNPs increase DALYs]

Sao Tome and Principe (STP)

--- INPUTS ---

Based on the 2017 Global Burden of Disease analysis, the incidence rates of diarrhoea are: mild = 1.4 events per year per child; moderate = 0.77 per year per child; severe = 0.011 per year per child.

Based on the 2017 Global Burden of Disease analysis, the incidence rates of malaria are: mild = 0.0079 per year per child; moderate = 0.0042 per year per child; severe = 6.1e-05 per year per child.

Based on the most recent Demographic and Health Survey (or equivalent), the prevalences of anaemia are:
6-8 months mild 30 percent, moderate 48 percent, severe 4.5 percent
9-11 months mild 36 percent, moderate 45 percent, severe 1.2 percent
12-17 months mild 32 percent, moderate 41 percent, severe 2.8 percent.

Background mortality rates through the treatment period are 2.42 percent per year on average.

Case-specific mortality rates are 0.00458 percent for a case of diarrhoea; 0.0342 percent for a case of malaria.

Sao Tome and Principe is classified as having endemic malaria, and has an Insecticide Treated bednet coverage rate of 61 percent based on 2015 UNICEF bednet coverage data.

In this country, the cost of an outpatient clinic visit is $0, and of hospitalisation is $0. We modelled the cost of MNPs as US $9.9 per course (including implementation).

--- OUTPUTS ---

Treatment with micronutrient powders caused a 5 percent increase in the incidence of diarrhoea, and a 2 percent increase in the incidence of malaria.
Treatment with micronutrient powders produced a 19 percent absolute decrease in the prevalence of anaemia at 12 months of age, and a 23 percent relative decrease.

Treatment with micronutrient powders caused an additional 0.223 deaths per 10,000 children [95 percent CIs = 0.988 to -0.379].

If coverage of the intervention is 100 percent, universal home fortification with micronutrient powders (MNPs) from age 6-12 months averted 40.2 [95 percent CIs = -18.2 to 87] DALYs per 10,000 children, compared with a control cohort. Of these averted DALYs, -14.9 [95 percent CIs = -66 to 25.3] were YLLs and 55.1 [95 percent CIs = 47.8 to 61.6] were YLDs.

When the effects of micronutrient powders on anaemia alone (excluding effects on diarrhoea and malaria) are considered, treatment with micronutrient powders caused a change in YLDs per 10,000 children of 57.

Treatment with micronutrient powders caused a net increase/decrease in healthcare costs of USD $9.9 per DALY averted.

If coverage was:
* 100 percent, net DALYs per 10,000 children averted was 40.2.
* 75 percent, net DALYs per 10,000 children averted was 30.
* 50 percent, net DALYs per 10,000 children averted was 20.1.
* 25 percent, net DALYs per 10,000 children averted was 9.98.

If coverage was:
* 100 percent: cost per DALY averted was $2464.
* 75 percent: cost per DALY averted was $3296.
* 50 percent: cost per DALY averted was $4926.

* 25 percent: cost per DALY averted was $9922.

Lesotho (LSO)

--- INPUTS ---

Based on the 2017 Global Burden of Disease analysis, the incidence rates of diarrhoea are: mild = 1 events per year per child; moderate = 0.56 per year per child; severe = 0.008 per year per child.

Based on the 2017 Global Burden of Disease analysis, the incidence rates of malaria are: mild = 0 per year per child; moderate = 0 per year per child; severe = 0 per year per child.

Based on the most recent Demographic and Health Survey (or equivalent), the prevalences of anaemia are:
6-8 months mild 23 percent, moderate 33 percent, severe 1.8 percent
9-11 months mild 30 percent, moderate 34 percent, severe 1.4 percent
12-17 months mild 24 percent, moderate 36 percent, severe 2.4 percent.

Background mortality rates through the treatment period are 4.84 percent per year on average.

Case-specific mortality rates are 0.0633 percent for a case of diarrhoea; NaN percent for a case of malaria.

Lesotho is not classified as having endemic malaria, and has an Insecticide Treated bednet coverage rate of 0 percent based on 2015 UNICEF bednet coverage data.

In this country, the cost of an outpatient clinic visit is $1.05, and of hospitalisation is $4.02. We modelled the cost of MNPs as US $9.9 per course (including implementation).

--- OUTPUTS ---

Treatment with micronutrient powders caused a 5 percent increase in the incidence of diarrhoea, and a NaN percent decrease in the incidence of malaria.
Treatment with micronutrient powders produced a 14 percent absolute decrease in the prevalence of anaemia at 12 months of age, and a 22 percent relative decrease.

Treatment with micronutrient powders caused an additional 0.703 deaths per 10,000 children [95 percent CIs = 2.82 to -1.22].

If coverage of the intervention is 100 percent, universal home fortification with micronutrient powders (MNPs) from age 6-12 months averted 6.38 [95 percent CIs = -112 to 115] DALYs per 10,000 children, compared with a control cohort. Of these averted DALYs, -37.2 [95 percent CIs = -149 to 64.6] were YLLs and 43.6 [95 percent CIs = 37.4 to 50.6] were YLDs.

When the effects of micronutrient powders on anaemia alone (excluding effects on diarrhoea and malaria) are considered, treatment with

micronutrient powders caused a change in YLDs per 10,000 children of 45.1.

Treatment with micronutrient powders caused a net increase/decrease in healthcare costs of USD $10 per DALY averted.

If coverage was:
* 100 percent, net DALYs per 10,000 children averted was 6.38.
* 75 percent, net DALYs per 10,000 children averted was 4.68.
* 50 percent, net DALYs per 10,000 children averted was 3.16.
* 25 percent, net DALYs per 10,000 children averted was 1.53.

If coverage was:
* 100 percent: cost per DALY averted was $15679.
* 75 percent: cost per DALY averted was $21351.
* 50 percent: cost per DALY averted was $31464.
* 25 percent: cost per DALY averted was $64793.

Malawi (MWI)

--- INPUTS ---

Based on the 2017 Global Burden of Disease analysis, the incidence rates of diarrhoea are: mild = 2 events per year per child; moderate = 1.1 per year per child; severe = 0.015 per year per child.

Based on the 2017 Global Burden of Disease analysis, the incidence rates of malaria are: mild = 0.39 per year per child; moderate = 0.21 per year per child; severe = 0.003 per year per child.

Based on the most recent Demographic and Health Survey (or equivalent), the prevalences of anaemia are:
6-8 months mild 24 percent, moderate 49 percent, severe 7.3 percent
9-11 months mild 23 percent, moderate 57 percent, severe 4.9 percent
12-17 months mild 24 percent, moderate 48 percent, severe 3.4 percent.

Background mortality rates through the treatment period are 3.1 percent per year on average.

Case-specific mortality rates are 0.0172 percent for a case of diarrhoea; 0.136 percent for a case of malaria.

Malawi is classified as having endemic malaria, and has an Insecticide Treated bednet coverage rate of 66 percent based on 2015 UNICEF bednet coverage data.

In this country, the cost of an outpatient clinic visit is $0.335, and of hospitalisation is $1. We modelled the cost of MNPs as US $9.9 per course (including implementation).

--- OUTPUTS ---

Treatment with micronutrient powders caused a 4 percent increase in the incidence of diarrhoea, and a 2 percent increase in the incidence of malaria.

Treatment with micronutrient powders produced a 21 percent absolute decrease in the prevalence of anaemia at 12 months of age, and a 24 percent relative decrease.

Treatment with micronutrient powders caused an additional 1.35 deaths per 10,000 children [95 percent CIs = 5.69 to -2.32].

If coverage of the intervention is 100 percent, universal home fortification with micronutrient powders (MNPs) from age 6-12 months averted -9.43 [95 percent CIs = -270 to 212] DALYs per 10,000 children, compared with a control cohort. Of these averted DALYs, -77.9 [95 percent CIs = -327 to 134] were YLLs and 68.4 [95 percent CIs = 57.6 to 78.6] were YLDs.

When the effects of micronutrient powders on anaemia alone (excluding effects on diarrhoea and malaria) are considered, treatment with micronutrient powders caused a change in YLDs per 10,000 children of 70.9.

Treatment with micronutrient powders caused a net increase/decrease in healthcare costs of USD $9.95 per DALY averted.

If coverage was:
* 100 percent, net DALYs per 10,000 children averted was -9.43.
* 75 percent, net DALYs per 10,000 children averted was -7.82.
* 50 percent, net DALYs per 10,000 children averted was -5.31.
* 25 percent, net DALYs per 10,000 children averted was -3.07.

[Because our model predicts that treatment with micronutrient powders increases net DALYs, we did not calculate the effect on healthcare costs when MNPs increase DALYs]

Swaziland (SWZ)

--- INPUTS ---

Based on the 2017 Global Burden of Disease analysis, the incidence rates of diarrhoea are: mild = 1.3 events per year per child; moderate = 0.68 per year per child; severe = 0.0099 per year per child.

Based on the 2017 Global Burden of Disease analysis, the incidence rates of malaria are: mild = 5.8e-05 per year per child; moderate = 3.1e-05 per year per child; severe = 4.5e-07 per year per child.

Based on the most recent Demographic and Health Survey (or equivalent), the prevalences of anaemia are:
6-8 months mild 32 percent, moderate 32 percent, severe 1.4 percent
9-11 months mild 33 percent, moderate 35 percent, severe 0.9 percent
12-17 months mild 24 percent, moderate 36 percent, severe 3.1 percent.

Background mortality rates through the treatment period are 3.13 percent per year on average.

Case-specific mortality rates are 0.0321 percent for a case of diarrhoea; 13.1 percent for a case of malaria.

Swaziland is not classified as having endemic malaria, and has an Insecticide Treated bednet coverage rate of 2 percent based on 2015 UNICEF bednet coverage data.

In this country, the cost of an outpatient clinic visit is $3.14, and of hospitalisation is $17.5. We modelled the cost of MNPs as US $9.9 per course (including implementation).

--- OUTPUTS ---

Treatment with micronutrient powders caused a 5 percent increase in the incidence of diarrhoea, and a 1e+01 percent decrease in the incidence of malaria.
Treatment with micronutrient powders produced a 16 percent absolute decrease in the prevalence of anaemia at 12 months of age, and a 23 percent relative decrease.

Treatment with micronutrient powders caused an additional 0.559 deaths per 10,000 children [95 percent CIs = 2.53 to -0.891].

If coverage of the intervention is 100 percent, universal home fortification with micronutrient powders (MNPs) from age 6-12 months averted 12.5 [95 percent CIs = -109 to 103] DALYs per 10,000 children, compared with a control cohort. Of these averted DALYs, -32.5 [95 percent CIs = -147 to 51.8] were YLLs and 45 [95 percent CIs = 38 to 51.6] were YLDs.

When the effects of micronutrient powders on anaemia alone (excluding effects on diarrhoea and malaria) are considered, treatment with micronutrient powders caused a change in YLDs per 10,000 children of 46.7.

Treatment with micronutrient powders caused a net increase/decrease in healthcare costs of USD $10.3 per DALY averted.

If coverage was:
* 100 percent, net DALYs per 10,000 children averted was 12.5.
* 75 percent, net DALYs per 10,000 children averted was 9.24.
* 50 percent, net DALYs per 10,000 children averted was 6.21.
* 25 percent, net DALYs per 10,000 children averted was 3.02.

If coverage was:
* 100 percent: cost per DALY averted was $8225.
* 75 percent: cost per DALY averted was $11017.
* 50 percent: cost per DALY averted was $16261.
* 25 percent: cost per DALY averted was $33110.

Equatorial Guinea (GNQ)

--- INPUTS ---

Based on the 2017 Global Burden of Disease analysis, the incidence rates of diarrhoea are: mild = 1.5 events per year per child; moderate = 0.81 per year per child; severe = 0.012 per year per child.

Based on the 2017 Global Burden of Disease analysis, the incidence rates of malaria are: mild = 0.62 per year per child; moderate = 0.33 per year per child; severe = 0.0048 per year per child.

Based on the most recent Demographic and Health Survey (or equivalent), the prevalences of anaemia are:
6-8 months mild 21 percent, moderate 54 percent, severe 0 percent
9-11 months mild 34 percent, moderate 34 percent, severe 12 percent
12-17 months mild 23 percent, moderate 46 percent, severe 5.3 percent.

Background mortality rates through the treatment period are 4.81 percent per year on average.

Case-specific mortality rates are 0.00765 percent for a case of diarrhoea; 0.155 percent for a case of malaria.

Equatorial Guinea is classified as having endemic malaria, and has an Insecticide Treated bednet coverage rate of 23 percent based on 2015 UNICEF bednet coverage data.

In this country, the cost of an outpatient clinic visit is $26.9, and of hospitalisation is $275. We modelled the cost of MNPs as US $9.9 per course (including implementation).

--- OUTPUTS ---

Treatment with micronutrient powders caused a 5 percent increase in the incidence of diarrhoea, and a 6 percent increase in the incidence of malaria.
Treatment with micronutrient powders produced a 19 percent absolute decrease in the prevalence of anaemia at 12 months of age, and a 24 percent relative decrease.

Treatment with micronutrient powders caused an additional 3.13 deaths per 10,000 children [95 percent CIs = 6.7 to -0.265].

If coverage of the intervention is 100 percent, universal home fortification with micronutrient powders (MNPs) from age 6-12 months averted -107 [95 percent CIs = -321 to 97.2] DALYs per 10,000 children, compared with a control cohort. Of these averted DALYs, -180 [95 percent CIs = -385 to 15.2] were YLLs and 72.9 [95 percent CIs = 64.6 to 82] were YLDs.

When the effects of micronutrient powders on anaemia alone (excluding effects on diarrhoea and malaria) are considered, treatment with micronutrient powders caused a change in YLDs per 10,000 children of 75.4.

Treatment with micronutrient powders caused a net increase/decrease in healthcare costs of USD $16 per DALY averted.

If coverage was:
* 100 percent, net DALYs per 10,000 children averted was -107.
* 75 percent, net DALYs per 10,000 children averted was -80.7.
* 50 percent, net DALYs per 10,000 children averted was -53.9.
* 25 percent, net DALYs per 10,000 children averted was -27.4.

[Because our model predicts that treatment with micronutrient powders increases net DALYs, we did not calculate the effect on healthcare costs when MNPs increase DALYs]

Egypt (EGY)

--- INPUTS ---

Based on the 2017 Global Burden of Disease analysis, the incidence rates of diarrhoea are: mild = 1.4 events per year per child; moderate = 0.77 per year per child; severe = 0.011 per year per child.

Based on the 2017 Global Burden of Disease analysis, the incidence rates of malaria are: mild = 0 per year per child; moderate = 0 per year per child; severe = 0 per year per child.

Based on the most recent Demographic and Health Survey (or equivalent), the prevalences of anaemia are:
6-8 months mild 25 percent, moderate 17 percent, severe 0 percent
9-11 months mild 28 percent, moderate 21 percent, severe 0 percent
12-17 months mild 20 percent, moderate 22 percent, severe 0.1 percent.

Background mortality rates through the treatment period are 1.4 percent per year on average.

Case-specific mortality rates are 0.00601 percent for a case of diarrhoea; NaN percent for a case of malaria.

Egypt is not classified as having endemic malaria, and has an Insecticide Treated bednet coverage rate of 0 percent based on 2015 UNICEF bednet coverage data.

In this country, the cost of an outpatient clinic visit is $4.15, and of hospitalisation is $23.6. We modelled the cost of MNPs as US $9.9 per course (including implementation).

--- OUTPUTS ---

Treatment with micronutrient powders caused a 5 percent increase in the incidence of diarrhoea, and a NaN percent decrease in the incidence of malaria.
Treatment with micronutrient powders produced a 13 percent absolute decrease in the prevalence of anaemia at 12 months of age, and a 25 percent relative decrease.

Treatment with micronutrient powders caused an additional 0.0897 deaths per 10,000 children [95 percent CIs = 0.419 to -0.164].

If coverage of the intervention is 100 percent, universal home fortification with micronutrient powders (MNPs) from age 6-12 months averted 18.5 [95 percent CIs = -11.6 to 42.1] DALYs per 10,000 children, compared with a control cohort. Of these averted DALYs, -6.23 [95 percent CIs = -29.4 to 11.5] were YLLs and 24.7 [95 percent CIs = 17.8 to 30.6] were YLDs.

When the effects of micronutrient powders on anaemia alone (excluding effects on diarrhoea and malaria) are considered, treatment with

micronutrient powders caused a change in YLDs per 10,000 children of 26.4.

Treatment with micronutrient powders caused a net increase/decrease in healthcare costs of USD $10.4 per DALY averted.

If coverage was:
* 100 percent, net DALYs per 10,000 children averted was 18.5.
* 75 percent, net DALYs per 10,000 children averted was 13.8.
* 50 percent, net DALYs per 10,000 children averted was 9.29.
* 25 percent, net DALYs per 10,000 children averted was 4.62.

If coverage was:
* 100 percent: cost per DALY averted was $5652.
* 75 percent: cost per DALY averted was $7442.
* 50 percent: cost per DALY averted was $10955.
* 25 percent: cost per DALY averted was $21667.

Sudan (SDN)

--- INPUTS ---

Based on the 2017 Global Burden of Disease analysis, the incidence rates of diarrhoea are: mild = 2.5 events per year per child; moderate = 1.3 per year per child; severe = 0.019 per year per child.

Based on the 2017 Global Burden of Disease analysis, the incidence rates of malaria are: mild = 0.072 per year per child; moderate = 0.039 per year per child; severe = 0.00056 per year per child.

Based on the most recent Demographic and Health Survey (or equivalent), the prevalences of anaemia are:
6-8 months mild 29 percent, moderate 42 percent, severe 1.8 percent
9-11 months mild 31 percent, moderate 48 percent, severe 6.7 percent
12-17 months mild 30 percent, moderate 40 percent, severe 4.2 percent.

Background mortality rates through the treatment period are 3.38 percent per year on average.

Case-specific mortality rates are 0.0161 percent for a case of diarrhoea; 0.143 percent for a case of malaria.

Sudan is classified as having endemic malaria, and has an Insecticide Treated bednet coverage rate of 30 percent based on 2015 UNICEF bednet coverage data.

In this country, the cost of an outpatient clinic visit is $3.4, and of hospitalisation is $14.2. We modelled the cost of MNPs as US $9.9 per course (including implementation).

--- OUTPUTS ---

Treatment with micronutrient powders caused a 5 percent increase in the incidence of diarrhoea, and a 5 percent increase in the incidence of malaria.

Treatment with micronutrient powders produced a 23 percent absolute decrease in the prevalence of anaemia at 12 months of age, and a 26 percent relative decrease.

Treatment with micronutrient powders caused an additional 0.817 deaths per 10,000 children [95 percent CIs = 3.06 to -0.994].

If coverage of the intervention is 100 percent, universal home fortification with micronutrient powders (MNPs) from age 6-12 months averted 24.3 [95 percent CIs = -115 to 137] DALYs per 10,000 children, compared with a control cohort. Of these averted DALYs, -46.2 [95 percent CIs = -173 to 56.2] were YLLs and 70.5 [95 percent CIs = 58.6 to 81.1] were YLDs.

When the effects of micronutrient powders on anaemia alone (excluding effects on diarrhoea and malaria) are considered, treatment with micronutrient powders caused a change in YLDs per 10,000 children of 74.

Treatment with micronutrient powders caused a net increase/decrease in healthcare costs of USD $10.7 per DALY averted.

If coverage was:
* 100 percent, net DALYs per 10,000 children averted was 24.3.
* 75 percent, net DALYs per 10,000 children averted was 17.7.
* 50 percent, net DALYs per 10,000 children averted was 11.8.
* 25 percent, net DALYs per 10,000 children averted was 5.54.

If coverage was:
* 100 percent: cost per DALY averted was $4403.
* 75 percent: cost per DALY averted was $5934.
* 50 percent: cost per DALY averted was $8703.
* 25 percent: cost per DALY averted was $18208.

Asia and the Middle East

Yemen (YEM)

--- INPUTS ---

Based on the 2017 Global Burden of Disease analysis, the incidence rates of diarrhoea are: mild = 2.9 events per year per child; moderate = 1.5 per year per child; severe = 0.022 per year per child.

Based on the 2017 Global Burden of Disease analysis, the incidence rates of malaria are: mild = 0.023 per year per child; moderate = 0.012 per year per child; severe = 0.00017 per year per child.

Based on the most recent Demographic and Health Survey (or equivalent), the prevalences of anaemia are:
6-8 months mild 12 percent, moderate 60 percent, severe 16 percent
9-11 months mild 10 percent, moderate 64 percent, severe 22 percent
12-17 months mild 10 percent, moderate 61 percent, severe 21 percent.

Background mortality rates through the treatment period are 0.702 percent per year on average.

Case-specific mortality rates are 0.0134 percent for a case of diarrhoea; 1.82e-05 percent for a case of malaria.

Yemen is classified as having endemic malaria, and has an Insecticide Treated bednet coverage rate of 1 percent based on 2015 UNICEF bednet coverage data.

In this country, the cost of an outpatient clinic visit is $4.69, and of hospitalisation is $21.3. We modelled the cost of MNPs as US $9.9 per course (including implementation).

--- OUTPUTS ---

Treatment with micronutrient powders caused a 4 percent increase in the incidence of diarrhoea, and a 8 percent increase in the incidence of malaria.
Treatment with micronutrient powders produced a 21 percent absolute decrease in the prevalence of anaemia at 12 months of age, and a 21 percent relative decrease.

Treatment with micronutrient powders caused an additional 0.2 deaths per 10,000 children [95 percent CIs = 0.996 to -0.463].

If coverage of the intervention is 100 percent, universal home fortification with micronutrient powders (MNPs) from age 6-12 months averted 105 [95 percent CIs = 39.9 to 160] DALYs per 10,000 children, compared with a control cohort. Of these averted DALYs, -13 [95 percent CIs = -64.7 to 30.1] were YLLs and 118 [95 percent CIs = 105 to 130] were YLDs.

When the effects of micronutrient powders on anaemia alone (excluding effects on diarrhoea and malaria) are considered, treatment with micronutrient powders caused a change in YLDs per 10,000 children of 122.

Treatment with micronutrient powders caused a net increase/decrease in healthcare costs of USD $10.9 per DALY averted.

If coverage was:
* 100 percent, net DALYs per 10,000 children averted was 105.
* 75 percent, net DALYs per 10,000 children averted was 79.
* 50 percent, net DALYs per 10,000 children averted was 52.5.
* 25 percent, net DALYs per 10,000 children averted was 26.2.

If coverage was:
* 100 percent: cost per DALY averted was $1041.
* 75 percent: cost per DALY averted was $1353.
* 50 percent: cost per DALY averted was $1980.
* 25 percent: cost per DALY averted was $3889.

Kyrgyzstan (KGZ)

--- INPUTS ---

Based on the 2017 Global Burden of Disease analysis, the incidence rates of diarrhoea are: mild = 3.6 events per year per child; moderate = 1.9 per year per child; severe = 0.028 per year per child.

Based on the 2017 Global Burden of Disease analysis, the incidence rates of malaria are: mild = 0 per year per child; moderate = 0 per year per child; severe = 0 per year per child.

Based on the most recent Demographic and Health Survey (or equivalent), the prevalences of anaemia are:
6-8 months mild 26 percent, moderate 28 percent, severe 1.4 percent
9-11 months mild 28 percent, moderate 29 percent, severe 3 percent
12-17 months mild 26 percent, moderate 29 percent, severe 3.6 percent.

Background mortality rates through the treatment period are 0.347 percent per year on average.

Case-specific mortality rates are 0.00898 percent for a case of diarrhoea; 0 percent for a case of malaria.

Kyrgyzstan is not classified as having endemic malaria, and has an Insecticide Treated bednet coverage rate of 0 percent based on 2015 UNICEF bednet coverage data.

In this country, the cost of an outpatient clinic visit is $1.3, and of hospitalisation is $5.41. We modelled the cost of MNPs as US $9.9 per course (including implementation).

--- OUTPUTS ---

Treatment with micronutrient powders caused a 4 percent increase in the incidence of diarrhoea, and a NaN percent decrease in the incidence of malaria.
Treatment with micronutrient powders produced a 13 percent absolute decrease in the prevalence of anaemia at 12 months of age, and a 22 percent relative decrease.

Treatment with micronutrient powders caused an additional 0.357 deaths per 10,000 children [95 percent CIs = 1.82 to -0.921].

If coverage of the intervention is 100 percent, universal home fortification with micronutrient powders (MNPs) from age 6-12 months averted 15.4 [95 percent CIs = -98.9 to 115] DALYs per 10,000 children, compared with a control cohort. Of these averted DALYs, -23.7 [95 percent CIs = -121 to 61.2] were YLLs and 39.2 [95 percent CIs = 21.8 to 53.5] were YLDs.

When the effects of micronutrient powders on anaemia alone (excluding effects on diarrhoea and malaria) are considered, treatment with micronutrient powders caused a change in YLDs per 10,000 children of 43.6.

Treatment with micronutrient powders caused a net increase/decrease in healthcare costs of USD $10.3 per DALY averted.

If coverage was:
* 100 percent, net DALYs per 10,000 children averted was 15.4.
* 75 percent, net DALYs per 10,000 children averted was 11.6.
* 50 percent, net DALYs per 10,000 children averted was 7.67.
* 25 percent, net DALYs per 10,000 children averted was 3.81.

If coverage was:
* 100 percent: cost per DALY averted was $6674.
* 75 percent: cost per DALY averted was $8821.
* 50 percent: cost per DALY averted was $13166.
* 25 percent: cost per DALY averted was $26208.

Tajikistan (TJK)

--- INPUTS ---

Based on the 2017 Global Burden of Disease analysis, the incidence rates of diarrhoea are: mild = 0.86 events per year per child; moderate = 0.46 per year per child; severe = 0.0067 per year per child.

Based on the 2017 Global Burden of Disease analysis, the incidence rates of malaria are: mild = 0 per year per child; moderate = 0 per year per child; severe = 0 per year per child.

Based on the most recent Demographic and Health Survey (or equivalent), the prevalences of anaemia are:
6-8 months mild 19 percent, moderate 21 percent, severe 1 percent
9-11 months mild 21 percent, moderate 22 percent, severe 2.2 percent
12-17 months mild 19 percent, moderate 21 percent, severe 2.7 percent.

Background mortality rates through the treatment period are 0.739 percent per year on average.

Case-specific mortality rates are 0.0311 percent for a case of diarrhoea; NaN percent for a case of malaria.

Tajikistan is not classified as having endemic malaria, and has an Insecticide Treated bednet coverage rate of 0 percent based on 2015 UNICEF bednet coverage data.

In this country, the cost of an outpatient clinic visit is $0.971, and of hospitalisation is $3.91. We modelled the cost of MNPs as US $9.9 per course (including implementation).

--- OUTPUTS ---

Treatment with micronutrient powders caused a 5 percent increase in the incidence of diarrhoea, and a NaN percent decrease in the incidence of malaria.
Treatment with micronutrient powders produced a 11 percent absolute decrease in the prevalence of anaemia at 12 months of age, and a 24 percent relative decrease.

Treatment with micronutrient powders caused an additional 0.165 deaths per 10,000 children [95 percent CIs = 0.797 to -0.34].

If coverage of the intervention is 100 percent, universal home fortification with micronutrient powders (MNPs) from age 6-12 months averted 24.1 [95 percent CIs = -25.6 to 64.7] DALYs per 10,000 children, compared with a control cohort. Of these averted DALYs, -11.4 [95 percent CIs = -54.9 to 23.4] were YLLs and 35.4 [95 percent CIs = 29.4 to 41.3] were YLDs.

When the effects of micronutrient powders on anaemia alone (excluding effects on diarrhoea and malaria) are considered, treatment with micronutrient powders caused a change in YLDs per 10,000 children of 36.6.

Treatment with micronutrient powders caused a net increase/decrease in healthcare costs of USD $9.97 per DALY averted.

If coverage was:
* 100 percent, net DALYs per 10,000 children averted was 24.1.
* 75 percent, net DALYs per 10,000 children averted was 18.
* 50 percent, net DALYs per 10,000 children averted was 12.
* 25 percent, net DALYs per 10,000 children averted was 5.98.

If coverage was:
* 100 percent: cost per DALY averted was $4146.
* 75 percent: cost per DALY averted was $5531.
* 50 percent: cost per DALY averted was $8269.
* 25 percent: cost per DALY averted was $16589.

Indonesia (IDN)

--- INPUTS ---

Based on the 2017 Global Burden of Disease analysis, the incidence rates of diarrhoea are: mild = 1.4 events per year per child; moderate = 0.73 per year per child; severe = 0.011 per year per child.

Based on the 2017 Global Burden of Disease analysis, the incidence rates of malaria are: mild = 0.0052 per year per child; moderate = 0.0028 per year per child; severe = 4e-05 per year per child.

Based on the most recent Demographic and Health Survey (or equivalent), the prevalences of anaemia are:
6-8 months mild 25 percent, moderate 18 percent, severe 0.78 percent
9-11 months mild 26 percent, moderate 15 percent, severe 1.6 percent
12-17 months mild 24 percent, moderate 17 percent, severe 0 percent.

Background mortality rates through the treatment period are 0.452 percent per year on average.

Case-specific mortality rates are 0.00661 percent for a case of diarrhoea; 0.1 percent for a case of malaria.

Indonesia is classified as having endemic malaria, and has an Insecticide Treated bednet coverage rate of 3 percent based on 2015 UNICEF bednet coverage data.

In this country, the cost of an outpatient clinic visit is $4.49, and of hospitalisation is $23.1. We modelled the cost of MNPs as US $9.9 per course (including implementation).

--- OUTPUTS ---

Treatment with micronutrient powders caused a 5 percent increase in the incidence of diarrhoea, and a 7 percent increase in the incidence of malaria.
Treatment with micronutrient powders produced a 11 percent absolute decrease in the prevalence of anaemia at 12 months of age, and a 27 percent relative decrease.

Treatment with micronutrient powders caused an additional 0.123 deaths per 10,000 children [95 percent CIs = 0.522 to -0.23].

If coverage of the intervention is 100 percent, universal home fortification with micronutrient powders (MNPs) from age 6-12 months averted 18.7 [95 percent CIs = -15.7 to 49.5] DALYs per 10,000 children, compared with a control cohort. Of these averted DALYs, -8.41 [95 percent CIs = -35.6 to 15.7] were YLLs and 27.1 [95 percent CIs = 20 to 33.7] were YLDs.

When the effects of micronutrient powders on anaemia alone (excluding effects on diarrhoea and malaria) are considered, treatment with micronutrient powders caused a change in YLDs per 10,000 children of 28.9.

Treatment with micronutrient powders caused a net increase/decrease in healthcare costs of USD $10.5 per DALY averted.

If coverage was:
* 100 percent, net DALYs per 10,000 children averted was 18.7.
* 75 percent, net DALYs per 10,000 children averted was 13.9.
* 50 percent, net DALYs per 10,000 children averted was 9.22.
* 25 percent, net DALYs per 10,000 children averted was 4.61.

If coverage was:
* 100 percent: cost per DALY averted was $5638.
* 75 percent: cost per DALY averted was $7474.
* 50 percent: cost per DALY averted was $11086.
* 25 percent: cost per DALY averted was $21822.

Mongolia (MNG)

--- INPUTS ---

Based on the 2017 Global Burden of Disease analysis, the incidence rates of diarrhoea are: mild = 0.79 events per year per child; moderate = 0.42 per year per child; severe = 0.0061 per year per child.

Based on the 2017 Global Burden of Disease analysis, the incidence rates of malaria are: mild = 0 per year per child; moderate = 0 per year per child; severe = 0 per year per child.

Based on the most recent Demographic and Health Survey (or equivalent), the prevalences of anaemia are:
6-8 months mild 22 percent, moderate 8.7 percent, severe 0 percent
9-11 months mild 18 percent, moderate 3.6 percent, severe 0 percent
12-17 months mild 22 percent, moderate 24 percent, severe 0 percent.

Background mortality rates through the treatment period are 0.369 percent per year on average.

Case-specific mortality rates are 0.00114 percent for a case of diarrhoea; NaN percent for a case of malaria.

Mongolia is not classified as having endemic malaria, and has an Insecticide Treated bednet coverage rate of 0 percent based on 2015 UNICEF bednet coverage data.

In this country, the cost of an outpatient clinic visit is $3.46, and of hospitalisation is $17. We modelled the cost of MNPs as US $9.9 per course (including implementation).

--- OUTPUTS ---

Treatment with micronutrient powders caused a 4 percent increase in the incidence of diarrhoea, and a NaN percent decrease in the incidence of malaria.
Treatment with micronutrient powders produced a 4.5 percent absolute decrease in the prevalence of anaemia at 12 months of age, and a 21 percent relative decrease.

Treatment with micronutrient powders caused an additional 0.00381 deaths per 10,000 children [95 percent CIs = 0.0199 to -0.0094].

If coverage of the intervention is 100 percent, universal home fortification with micronutrient powders (MNPs) from age 6-12 months averted 18.7 [95 percent CIs = 12.9 to 24] DALYs per 10,000 children, compared with a control cohort. Of these averted DALYs, -0.259 [95 percent CIs = -1.35 to 0.64] were YLLs and 18.9 [95 percent CIs = 14.3 to 23.4] were YLDs.

When the effects of micronutrient powders on anaemia alone (excluding effects on diarrhoea and malaria) are considered, treatment with micronutrient powders caused a change in YLDs per 10,000 children of 19.9.

Treatment with micronutrient powders caused a net increase/decrease in healthcare costs of USD $10.1 per DALY averted.

If coverage was:
* 100 percent, net DALYs per 10,000 children averted was 18.7.
* 75 percent, net DALYs per 10,000 children averted was 14.
* 50 percent, net DALYs per 10,000 children averted was 9.35.
* 25 percent, net DALYs per 10,000 children averted was 4.67.

If coverage was:
* 100 percent: cost per DALY averted was $5423.
* 75 percent: cost per DALY averted was $7190.
* 50 percent: cost per DALY averted was $10706.
* 25 percent: cost per DALY averted was $21296.

China (CHN)

--- INPUTS ---

Based on the 2017 Global Burden of Disease analysis, the incidence rates of diarrhoea are: mild = 0.66 events per year per child; moderate = 0.35 per year per child; severe = 0.0051 per year per child.

Based on the 2017 Global Burden of Disease analysis, the incidence rates of malaria are: mild = 3.2e-09 per year per child; moderate = 1.7e-09 per year per child; severe = 2.5e-11 per year per child.

Based on the most recent Demographic and Health Survey (or equivalent), the prevalences of anaemia are:
6-8 months mild 10 percent, moderate 16 percent, severe 0.21 percent
9-11 months mild 11 percent, moderate 18 percent, severe 0.24 percent
12-17 months mild 10 percent, moderate 16 percent, severe 0.52 percent.

Background mortality rates through the treatment period are 0.178 percent per year on average.

Case-specific mortality rates are 0.000556 percent for a case of diarrhoea; 108 percent for a case of malaria.

China is not classified as having endemic malaria, and has an Insecticide Treated bednet coverage rate of 0 percent based on 2015 UNICEF bednet coverage data.

In this country, the cost of an outpatient clinic visit is $5.82, and of hospitalisation is $34. We modelled the cost of MNPs as US $9.9 per course (including implementation).

--- OUTPUTS ---

Treatment with micronutrient powders caused a 4 percent increase in the incidence of diarrhoea, and a NaN percent decrease in the incidence of malaria.
Treatment with micronutrient powders produced a 7.8 percent absolute decrease in the prevalence of anaemia at 12 months of age, and a 28 percent relative decrease.

Treatment with micronutrient powders caused an additional 0.0146 deaths per 10,000 children [95 percent CIs = 0.0755 to -0.0334].

If coverage of the intervention is 100 percent, universal home fortification with micronutrient powders (MNPs) from age 6-12 months averted 25.5 [95 percent CIs = 16.2 to 33.3] DALYs per 10,000 children, compared with a control cohort. Of these averted DALYs, -1.1 [95 percent CIs = -5.69 to 2.52] were YLLs and 26.6 [95 percent CIs = 21.9 to 30.7] were YLDs.

When the effects of micronutrient powders on anaemia alone (excluding effects on diarrhoea and malaria) are considered, treatment with micronutrient powders caused a change in YLDs per 10,000 children of 27.3.

Treatment with micronutrient powders caused a net increase/decrease in healthcare costs of USD $10.2 per DALY averted.

If coverage was:
* 100 percent, net DALYs per 10,000 children averted was 25.5.
* 75 percent, net DALYs per 10,000 children averted was 19.
* 50 percent, net DALYs per 10,000 children averted was 12.8.
* 25 percent, net DALYs per 10,000 children averted was 6.38.

If coverage was:
* 100 percent: cost per DALY averted was $4020.
* 75 percent: cost per DALY averted was $5336.
* 50 percent: cost per DALY averted was $7883.
* 25 percent: cost per DALY averted was $15622.

Philippines (PHL)

--- INPUTS ---

Based on the 2017 Global Burden of Disease analysis, the incidence rates of diarrhoea are: mild = 0.82 events per year per child; moderate = 0.44 per year per child; severe = 0.0064 per year per child.

Based on the 2017 Global Burden of Disease analysis, the incidence rates of malaria are: mild = 3.1e-05 per year per child; moderate = 1.6e-05 per year per child; severe = 2.4e-07 per year per child.

Based on the most recent Demographic and Health Survey (or equivalent), the prevalences of anaemia are:
6-8 months mild 27 percent, moderate 19 percent, severe 0.62 percent
9-11 months mild 28 percent, moderate 23 percent, severe 0.62 percent
12-17 months mild 21 percent, moderate 18 percent, severe 0.62 percent.

Background mortality rates through the treatment period are 0.458 percent per year on average.

Case-specific mortality rates are 0.014 percent for a case of diarrhoea; 0.787 percent for a case of malaria.

Philippines is classified as having endemic malaria, and has an Insecticide Treated bednet coverage rate of 0 percent based on 2015 UNICEF bednet coverage data.

In this country, the cost of an outpatient clinic visit is $3.54, and of hospitalisation is $17.6. We modelled the cost of MNPs as US $9.9 per course (including implementation).

--- OUTPUTS ---

Treatment with micronutrient powders caused a 5 percent increase in the incidence of diarrhoea, and a 6 percent increase in the incidence of malaria.
Treatment with micronutrient powders produced a 15 percent absolute decrease in the prevalence of anaemia at 12 months of age, and a 28 percent relative decrease.

Treatment with micronutrient powders caused an additional 0.135 deaths per 10,000 children [95 percent CIs = 0.612 to -0.262].

If coverage of the intervention is 100 percent, universal home fortification with micronutrient powders (MNPs) from age 6-12 months averted 22 [95 percent CIs = -15.4 to 53.5] DALYs per 10,000 children, compared with a control cohort. Of these averted DALYs, -9.15 [95 percent CIs = -41.4 to 17.8] were YLLs and 31.1 [95 percent CIs = 26 to 35.8] were YLDs.

When the effects of micronutrient powders on anaemia alone (excluding effects on diarrhoea and malaria) are considered, treatment with micronutrient powders caused a change in YLDs per 10,000 children of 32.3.

Treatment with micronutrient powders caused a net increase/decrease in healthcare costs of USD $10.2 per DALY averted.

If coverage was:
* 100 percent, net DALYs per 10,000 children averted was 22.
* 75 percent, net DALYs per 10,000 children averted was 16.5.
* 50 percent, net DALYs per 10,000 children averted was 10.9.
* 25 percent, net DALYs per 10,000 children averted was 5.51.

If coverage was:
* 100 percent: cost per DALY averted was $4630.
* 75 percent: cost per DALY averted was $6131.
* 50 percent: cost per DALY averted was $9220.
* 25 percent: cost per DALY averted was $18090.

Myanmar (MMR)

--- INPUTS ---

Based on the 2017 Global Burden of Disease analysis, the incidence rates of diarrhoea are: mild = 1.1 events per year per child; moderate = 0.58 per year per child; severe = 0.0083 per year per child.

Based on the 2017 Global Burden of Disease analysis, the incidence rates of malaria are: mild = 0.0012 per year per child; moderate = 0.00065 per year per child; severe = 9.4e-06 per year per child.

Based on the most recent Demographic and Health Survey (or equivalent), the prevalences of anaemia are:
6-8 months mild 37 percent, moderate 44 percent, severe 0 percent
9-11 months mild 34 percent, moderate 40 percent, severe 0.8 percent
12-17 months mild 32 percent, moderate 45 percent, severe 0.7 percent.

Background mortality rates through the treatment period are 0.85 percent per year on average.

Case-specific mortality rates are 0.00868 percent for a case of diarrhoea; 2.64 percent for a case of malaria.

Myanmar is classified as having endemic malaria, and has an Insecticide Treated bednet coverage rate of 11 percent based on 2015 UNICEF bednet coverage data.

In this country, the cost of an outpatient clinic visit is $1.17, and of hospitalisation is $4.02. We modelled the cost of MNPs as US $9.9 per course (including implementation).

--- OUTPUTS ---

Treatment with micronutrient powders caused a 5 percent increase in the incidence of diarrhoea, and a 1e+01 percent increase in the incidence of malaria.
Treatment with micronutrient powders produced a 17 percent absolute decrease in the prevalence of anaemia at 12 months of age, and a 23 percent relative decrease.

Treatment with micronutrient powders caused an additional 0.165 deaths per 10,000 children [95 percent CIs = 0.724 to -0.303].

If coverage of the intervention is 100 percent, universal home fortification with micronutrient powders (MNPs) from age 6-12 months averted 40.5 [95 percent CIs = -2.88 to 77.6] DALYs per 10,000 children, compared with a control cohort. Of these averted DALYs, -10.9 [95 percent CIs = -47.7 to 19.9] were YLLs and 51.4 [95 percent CIs = 44.8 to 57.7] were YLDs.

When the effects of micronutrient powders on anaemia alone (excluding effects on diarrhoea and malaria) are considered, treatment with micronutrient powders caused a change in YLDs per 10,000 children of 52.9.

Treatment with micronutrient powders caused a net increase/decrease in healthcare costs of USD $10 per DALY averted.

If coverage was:

* 100 percent, net DALYs per 10,000 children averted was 40.5.
* 75 percent, net DALYs per 10,000 children averted was 30.3.
* 50 percent, net DALYs per 10,000 children averted was 20.2.
* 25 percent, net DALYs per 10,000 children averted was 10.

If coverage was:
* 100 percent: cost per DALY averted was $2474.
* 75 percent: cost per DALY averted was $3291.
* 50 percent: cost per DALY averted was $4940.
* 25 percent: cost per DALY averted was $9883.

Solomon Islands (SLB)

--- INPUTS ---

Based on the 2017 Global Burden of Disease analysis, the incidence rates of diarrhoea are: mild = 1.7 events per year per child; moderate = 0.89 per year per child; severe = 0.013 per year per child.

Based on the 2017 Global Burden of Disease analysis, the incidence rates of malaria are: mild = 0.14 per year per child; moderate = 0.073 per year per child; severe = 0.0011 per year per child.

Based on the most recent Demographic and Health Survey (or equivalent), the prevalences of anaemia are:
6-8 months mild 36 percent, moderate 24 percent, severe 1.6 percent
9-11 months mild 28 percent, moderate 28 percent, severe 2.6 percent
12-17 months mild 33 percent, moderate 29 percent, severe 1.1 percent.

Background mortality rates through the treatment period are 0.46 percent per year on average.

Case-specific mortality rates are 0.00515 percent for a case of diarrhoea; 3.56e-05 percent for a case of malaria.

Solomon Islands is classified as having endemic malaria, and has an Insecticide Treated bednet coverage rate of 40 percent based on 2015 UNICEF bednet coverage data.

In this country, the cost of an outpatient clinic visit is $2.92, and of hospitalisation is $12.8. We modelled the cost of MNPs as US $9.9 per course (including implementation).

--- OUTPUTS ---

Treatment with micronutrient powders caused a 5 percent increase in the incidence of diarrhoea, and a 5 percent increase in the incidence of malaria.
Treatment with micronutrient powders produced a 15 percent absolute decrease in the prevalence of anaemia at 12 months of age, and a 25 percent relative decrease.

Treatment with micronutrient powders caused an additional 0.226 deaths per 10,000 children [95 percent CIs = 0.874 to -0.267].

If coverage of the intervention is 100 percent, universal home fortification with micronutrient powders (MNPs) from age 6-12 months averted 26.2 [95 percent CIs = -26.6 to 67.6] DALYs per 10,000 children, compared with a control cohort. Of these averted DALYs, -15.5 [95 percent CIs = -59.8 to 18.2] were YLLs and 41.6 [95 percent CIs = 33.2 to 49.3] were YLDs.

When the effects of micronutrient powders on anaemia alone (excluding effects on diarrhoea and malaria) are considered, treatment with micronutrient powders caused a change in YLDs per 10,000 children of 44.

Treatment with micronutrient powders caused a net increase/decrease in healthcare costs of USD $10.4 per DALY averted.

If coverage was:
* 100 percent, net DALYs per 10,000 children averted was 26.2.
* 75 percent, net DALYs per 10,000 children averted was 19.5.
* 50 percent, net DALYs per 10,000 children averted was 13.
* 25 percent, net DALYs per 10,000 children averted was 6.42.

If coverage was:
* 100 percent: cost per DALY averted was $3986.
* 75 percent: cost per DALY averted was $5278.
* 50 percent: cost per DALY averted was $7840.
* 25 percent: cost per DALY averted was $15606.

Timor Leste (TLS)

--- INPUTS ---

Based on the 2017 Global Burden of Disease analysis, the incidence rates of diarrhoea are: mild = 1.2 events per year per child; moderate = 0.64 per year per child; severe = 0.0093 per year per child.

Based on the 2017 Global Burden of Disease analysis, the incidence rates of malaria are: mild = 2.6e-05 per year per child; moderate = 1.4e-05 per year per child; severe = 2e-07 per year per child.

Based on the most recent Demographic and Health Survey (or equivalent), the prevalences of anaemia are:
6-8 months mild 35 percent, moderate 26 percent, severe 1.1 percent
9-11 months mild 37 percent, moderate 21 percent, severe 2.3 percent
12-17 months mild 34 percent, moderate 23 percent, severe 0 percent.

Background mortality rates through the treatment period are 0.877 percent per year on average.

Case-specific mortality rates are 0.0108 percent for a case of diarrhoea; 2.19 percent for a case of malaria.

Timor Leste is classified as having endemic malaria, and has an Insecticide Treated bednet coverage rate of 41 percent based on 2015 UNICEF bednet coverage data.

In this country, the cost of an outpatient clinic visit is $1.31, and of hospitalisation is $4.11. We modelled the cost of MNPs as US $9.9 per course (including implementation).

--- OUTPUTS ---

Treatment with micronutrient powders caused a 5 percent increase in the incidence of diarrhoea, and a 1e+01 percent increase in the incidence of malaria.
Treatment with micronutrient powders produced a 16 percent absolute decrease in the prevalence of anaemia at 12 months of age, and a 27 percent relative decrease.

Treatment with micronutrient powders caused an additional 0.142 deaths per 10,000 children [95 percent CIs = 0.623 to -0.258].

If coverage of the intervention is 100 percent, universal home fortification with micronutrient powders (MNPs) from age 6-12 months averted 27.1 [95 percent CIs = -11.9 to 60.3] DALYs per 10,000 children, compared with a control cohort. Of these averted DALYs, -9.56 [95 percent CIs = -42.1 to 17.4] were YLLs and 36.7 [95 percent CIs = 30.1 to 42.9] were YLDs.

When the effects of micronutrient powders on anaemia alone (excluding effects on diarrhoea and malaria) are considered, treatment with micronutrient powders caused a change in YLDs per 10,000 children of 38.3.

Treatment with micronutrient powders caused a net increase/decrease in healthcare costs of USD $10 per DALY averted.

If coverage was:
* 100 percent, net DALYs per 10,000 children averted was 27.1.
* 75 percent, net DALYs per 10,000 children averted was 20.3.
* 50 percent, net DALYs per 10,000 children averted was 13.5.
* 25 percent, net DALYs per 10,000 children averted was 6.81.

If coverage was:
* 100 percent: cost per DALY averted was $3701.
* 75 percent: cost per DALY averted was $4930.
* 50 percent: cost per DALY averted was $7370.
* 25 percent: cost per DALY averted was $14591.

India (IND)

--- INPUTS ---

Based on the 2017 Global Burden of Disease analysis, the incidence rates of diarrhoea are: mild = 0.5 events per year per child; moderate = 0.27 per year per child; severe = 0.0039 per year per child.

Based on the 2017 Global Burden of Disease analysis, the incidence rates of malaria are: mild = 0.0053 per year per child; moderate = 0.0028 per year per child; severe = 4.1e-05 per year per child.

Based on the most recent Demographic and Health Survey (or equivalent), the prevalences of anaemia are:
6-8 months mild 30 percent, moderate 37 percent, severe 1.4 percent
9-11 months mild 28 percent, moderate 38 percent, severe 2.1 percent
12-17 months mild 28 percent, moderate 41 percent, severe 2.4 percent.

Background mortality rates through the treatment period are 0.816 percent per year on average.

Case-specific mortality rates are 0.0309 percent for a case of diarrhoea; 1.35 percent for a case of malaria.

India is classified as having endemic malaria, and has an Insecticide Treated bednet coverage rate of 0 percent based on 2015 UNICEF bednet coverage data.

In this country, the cost of an outpatient clinic visit is $2.1, and of hospitalisation is $9.77. We modelled the cost of MNPs as US $9.9 per course (including implementation).

--- OUTPUTS ---

Treatment with micronutrient powders caused a 5 percent increase in the incidence of diarrhoea, and a 7 percent increase in the incidence of malaria.
Treatment with micronutrient powders produced a 18 percent absolute decrease in the prevalence of anaemia at 12 months of age, and a 27 percent relative decrease.

Treatment with micronutrient powders caused an additional 0.122 deaths per 10,000 children [95 percent CIs = 0.546 to -0.246].

If coverage of the intervention is 100 percent, universal home fortification with micronutrient powders (MNPs) from age 6-12 months averted 54.4 [95 percent CIs = 20.3 to 85] DALYs per 10,000 children, compared with a control cohort. Of these averted DALYs, -8.26 [95 percent CIs = -36.9 to 16.6] were YLLs and 62.6 [95 percent CIs = 57.2 to 68.4] were YLDs.

When the effects of micronutrient powders on anaemia alone (excluding effects on diarrhoea and malaria) are considered, treatment with micronutrient powders caused a change in YLDs per 10,000 children of 63.3.

Treatment with micronutrient powders caused a net increase/decrease in healthcare costs of USD $10 per DALY averted.

If coverage was:
* 100 percent, net DALYs per 10,000 children averted was 54.4.
* 75 percent, net DALYs per 10,000 children averted was 40.6.
* 50 percent, net DALYs per 10,000 children averted was 27.2.
* 25 percent, net DALYs per 10,000 children averted was 13.5.

If coverage was:
* 100 percent: cost per DALY averted was $1840.
* 75 percent: cost per DALY averted was $2454.
* 50 percent: cost per DALY averted was $3663.

* 25 percent: cost per DALY averted was $7347.

Pakistan (PAK)

--- INPUTS ---

Based on the 2017 Global Burden of Disease analysis, the incidence rates of diarrhoea are: mild = 1.2 events per year per child; moderate = 0.63 per year per child; severe = 0.009 per year per child.

Based on the 2017 Global Burden of Disease analysis, the incidence rates of malaria are: mild = 0.0091 per year per child; moderate = 0.0049 per year per child; severe = 7e-05 per year per child.

Based on the most recent Demographic and Health Survey (or equivalent), the prevalences of anaemia are:
6-8 months mild 28 percent, moderate 30 percent, severe 4.5 percent
9-11 months mild 26 percent, moderate 37 percent, severe 4.8 percent
12-17 months mild 26 percent, moderate 42 percent, severe 4.8 percent.

Background mortality rates through the treatment period are 1.37 percent per year on average.

Case-specific mortality rates are 0.026 percent for a case of diarrhoea; 0.181 percent for a case of malaria.

Pakistan is not classified as having endemic malaria, and has an Insecticide Treated bednet coverage rate of 0 percent based on 2015 UNICEF bednet coverage data.

In this country, the cost of an outpatient clinic visit is $1.82, and of hospitalisation is $8.06. We modelled the cost of MNPs as US $9.9 per course (including implementation).

--- OUTPUTS ---

Treatment with micronutrient powders caused a 5 percent increase in the incidence of diarrhoea, and a 4 percent decrease in the incidence of malaria.
Treatment with micronutrient powders produced a 15 percent absolute decrease in the prevalence of anaemia at 12 months of age, and a 22 percent relative decrease.

Treatment with micronutrient powders caused an additional 0.231 deaths per 10,000 children [95 percent CIs = 1.08 to -0.526].

If coverage of the intervention is 100 percent, universal home fortification with micronutrient powders (MNPs) from age 6-12 months averted 42.2 [95 percent CIs = -21.9 to 99.6] DALYs per 10,000 children, compared with a control cohort. Of these averted DALYs, -15.1 [95 percent CIs = -71.2 to 34.6] were YLLs and 57.3 [95 percent CIs = 49.3 to 65.1] were YLDs.

When the effects of micronutrient powders on anaemia alone (excluding effects on diarrhoea and malaria) are considered, treatment with

micronutrient powders caused a change in YLDs per 10,000 children of 58.9.

Treatment with micronutrient powders caused a net increase/decrease in healthcare costs of USD $10.1 per DALY averted.

If coverage was:
* 100 percent, net DALYs per 10,000 children averted was 42.2.
* 75 percent, net DALYs per 10,000 children averted was 31.5.
* 50 percent, net DALYs per 10,000 children averted was 21.
* 25 percent, net DALYs per 10,000 children averted was 10.3.

If coverage was:
* 100 percent: cost per DALY averted was $2387.
* 75 percent: cost per DALY averted was $3186.
* 50 percent: cost per DALY averted was $4746.
* 25 percent: cost per DALY averted was $9627.

Afghanistan (AFG)

--- INPUTS ---

Based on the 2017 Global Burden of Disease analysis, the incidence rates of diarrhoea are: mild = 2.5 events per year per child; moderate = 1.3 per year per child; severe = 0.019 per year per child.

Based on the 2017 Global Burden of Disease analysis, the incidence rates of malaria are: mild = 0.0052 per year per child; moderate = 0.0028 per year per child; severe = 4e-05 per year per child.

Based on the most recent Demographic and Health Survey (or equivalent), the prevalences of anaemia are:
6-8 months mild 30 percent, moderate 19 percent, severe 0.68 percent
9-11 months mild 19 percent, moderate 26 percent, severe 2 percent
12-17 months mild 18 percent, moderate 24 percent, severe 0.43 percent.

Background mortality rates through the treatment period are 1.6 percent per year on average.

Case-specific mortality rates are 0.00793 percent for a case of diarrhoea; 0.165 percent for a case of malaria.

Afghanistan is not classified as having endemic malaria, and has an Insecticide Treated bednet coverage rate of 0 percent based on 2015 UNICEF bednet coverage data.

In this country, the cost of an outpatient clinic visit is $1.26, and of hospitalisation is $3.86. We modelled the cost of MNPs as US $9.9 per course (including implementation).

--- OUTPUTS ---

Treatment with micronutrient powders caused a 5 percent increase in the incidence of diarrhoea, and a 3 percent decrease in the incidence of malaria.

Treatment with micronutrient powders produced a 13 percent absolute decrease in the prevalence of anaemia at 12 months of age, and a 27 percent relative decrease.

Treatment with micronutrient powders caused an additional 0.743 deaths per 10,000 children [95 percent CIs = 3.48 to -1.62].

If coverage of the intervention is 100 percent, universal home fortification with micronutrient powders (MNPs) from age 6-12 months averted -12.1 [95 percent CIs = -187 to 139] DALYs per 10,000 children, compared with a control cohort. Of these averted DALYs, -44.4 [95 percent CIs = -208 to 97] were YLLs and 32.3 [95 percent CIs = 20.9 to 42.4] were YLDs.

When the effects of micronutrient powders on anaemia alone (excluding effects on diarrhoea and malaria) are considered, treatment with micronutrient powders caused a change in YLDs per 10,000 children of 35.5.

Treatment with micronutrient powders caused a net increase/decrease in healthcare costs of USD $10.2 per DALY averted.

If coverage was:
* 100 percent, net DALYs per 10,000 children averted was -12.1.
* 75 percent, net DALYs per 10,000 children averted was -9.48.
* 50 percent, net DALYs per 10,000 children averted was -6.42.
* 25 percent, net DALYs per 10,000 children averted was -3.46.

[Because our model predicts that treatment with micronutrient powders increases net DALYs, we did not calculate the effect on healthcare costs when MNPs increase DALYs]
Nepal (NPL)

--- INPUTS ---

Based on the 2017 Global Burden of Disease analysis, the incidence rates of diarrhoea are: mild = 0.67 events per year per child; moderate = 0.36 per year per child; severe = 0.0052 per year per child.

Based on the 2017 Global Burden of Disease analysis, the incidence rates of malaria are: mild = 3.8e-05 per year per child; moderate = 2e-05 per year per child; severe = 2.9e-07 per year per child.

Based on the most recent Demographic and Health Survey (or equivalent), the prevalences of anaemia are:
6-8 months mild 37 percent, moderate 40 percent, severe 0.7 percent
9-11 months mild 37 percent, moderate 35 percent, severe 0.8 percent
12-17 months mild 38 percent, moderate 32 percent, severe 1.7 percent.

Background mortality rates through the treatment period are 0.628 percent per year on average.

Case-specific mortality rates are 0.00778 percent for a case of diarrhoea; 77.7 percent for a case of malaria.

Nepal is not classified as having endemic malaria, and has an Insecticide Treated bednet coverage rate of 0 percent based on 2015 UNICEF bednet coverage data.

In this country, the cost of an outpatient clinic visit is $0.926, and of hospitalisation is $3.14. We modelled the cost of MNPs as US $9.9 per course (including implementation).

--- OUTPUTS ---

Treatment with micronutrient powders caused a 5 percent increase in the incidence of diarrhoea, and a 8 percent decrease in the incidence of malaria.
Treatment with micronutrient powders produced a 13 percent absolute decrease in the prevalence of anaemia at 12 months of age, and a 18 percent relative decrease.

Treatment with micronutrient powders caused an additional 0.0412 deaths per 10,000 children [95 percent CIs = 0.188 to -0.0895].

If coverage of the intervention is 100 percent, universal home fortification with micronutrient powders (MNPs) from age 6-12 months averted 33.2 [95 percent CIs = 17.6 to 47.4] DALYs per 10,000 children, compared with a control cohort. Of these averted DALYs, -2.82 [95 percent CIs = -12.9 to 6.13] were YLLs and 36 [95 percent CIs = 30.5 to 41.3] were YLDs.

When the effects of micronutrient powders on anaemia alone (excluding effects on diarrhoea and malaria) are considered, treatment with micronutrient powders caused a change in YLDs per 10,000 children of 36.9.

Treatment with micronutrient powders caused a net increase/decrease in healthcare costs of USD $9.95 per DALY averted.

If coverage was:
* 100 percent, net DALYs per 10,000 children averted was 33.2.
* 75 percent, net DALYs per 10,000 children averted was 24.8.
* 50 percent, net DALYs per 10,000 children averted was 16.5.
* 25 percent, net DALYs per 10,000 children averted was 8.21.

If coverage was:
* 100 percent: cost per DALY averted was $2998.
* 75 percent: cost per DALY averted was $4003.
* 50 percent: cost per DALY averted was $6003.
* 25 percent: cost per DALY averted was $12082.

Bangladesh (BGD)

--- INPUTS ---

Based on the 2017 Global Burden of Disease analysis, the incidence rates of diarrhoea are: mild = 0.49 events per year per child; moderate = 0.26 per year per child; severe = 0.0038 per year per child.

Based on the 2017 Global Burden of Disease analysis, the incidence rates of malaria are: mild = 2.2e-05 per year per child; moderate = 1.2e-05 per year per child; severe = 1.7e-07 per year per child.

Based on the most recent Demographic and Health Survey (or equivalent), the prevalences of anaemia are:
6-8 months mild 27 percent, moderate 38 percent, severe 1.9 percent
9-11 months mild 29 percent, moderate 48 percent, severe 1.5 percent
12-17 months mild 32 percent, moderate 43 percent, severe 1.4 percent.

Background mortality rates through the treatment period are 0.623 percent per year on average.

Case-specific mortality rates are 0.0111 percent for a case of diarrhoea; 0.113 percent for a case of malaria.

Bangladesh is classified as having endemic malaria, and has an Insecticide Treated bednet coverage rate of 0 percent based on 2015 UNICEF bednet coverage data.

In this country, the cost of an outpatient clinic visit is $1.09, and of hospitalisation is $3.92. We modelled the cost of MNPs as US $9.9 per course (including implementation).

--- OUTPUTS ---

Treatment with micronutrient powders caused a 5 percent increase in the incidence of diarrhoea, and a 2e+01 percent decrease in the incidence of malaria.
Treatment with micronutrient powders produced a 22 percent absolute decrease in the prevalence of anaemia at 12 months of age, and a 28 percent relative decrease.

Treatment with micronutrient powders caused an additional 0.0348 deaths per 10,000 children [95 percent CIs = 0.152 to -0.0581].

If coverage of the intervention is 100 percent, universal home fortification with micronutrient powders (MNPs) from age 6-12 months averted 63.9 [95 percent CIs = 50.1 to 76] DALYs per 10,000 children, compared with a control cohort. Of these averted DALYs, -2.47 [95 percent CIs = -10.8 to 4.13] were YLLs and 66.4 [95 percent CIs = 60.9 to 71.9] were YLDs.

When the effects of micronutrient powders on anaemia alone (excluding effects on diarrhoea and malaria) are considered, treatment with micronutrient powders caused a change in YLDs per 10,000 children of 67.1.

Treatment with micronutrient powders caused a net increase/decrease in healthcare costs of USD $9.95 per DALY averted.

If coverage was:
* 100 percent, net DALYs per 10,000 children averted was 63.9.
* 75 percent, net DALYs per 10,000 children averted was 48.
* 50 percent, net DALYs per 10,000 children averted was 31.8.
* 25 percent, net DALYs per 10,000 children averted was 16.

If coverage was:
* 100 percent: cost per DALY averted was $1557.
* 75 percent: cost per DALY averted was $2070.
* 50 percent: cost per DALY averted was $3117.
* 25 percent: cost per DALY averted was $6183.

Bhutan (BTN)

--- INPUTS ---

Based on the 2017 Global Burden of Disease analysis, the incidence rates of diarrhoea are: mild = 0.81 events per year per child; moderate = 0.44 per year per child; severe = 0.0063 per year per child.

Based on the 2017 Global Burden of Disease analysis, the incidence rates of malaria are: mild = 6.7e-06 per year per child; moderate = 3.6e-06 per year per child; severe = 5.2e-08 per year per child.

Based on the most recent Demographic and Health Survey (or equivalent), the prevalences of anaemia are:
6-8 months mild 40 percent, moderate 44 percent, severe 0.75 percent
9-11 months mild 40 percent, moderate 38 percent, severe 0.86 percent
12-17 months mild 42 percent, moderate 35 percent, severe 1.8 percent.

Background mortality rates through the treatment period are 0.536 percent per year on average.

Case-specific mortality rates are 0.00194 percent for a case of diarrhoea; 450 percent for a case of malaria.

Bhutan is not classified as having endemic malaria, and has an Insecticide Treated bednet coverage rate of 0 percent based on 2015 UNICEF bednet coverage data.

In this country, the cost of an outpatient clinic visit is $2.64, and of hospitalisation is $14.4. We modelled the cost of MNPs as US $9.9 per course (including implementation).

--- OUTPUTS ---

Treatment with micronutrient powders caused a 5 percent increase in the incidence of diarrhoea, and a 4e+01 percent increase in the incidence of malaria.
Treatment with micronutrient powders produced a 14 percent absolute decrease in the prevalence of anaemia at 12 months of age, and a 17 percent relative decrease.

Treatment with micronutrient powders caused an additional 0.0366 deaths per 10,000 children [95 percent CIs = 0.595 to -0.514].

If coverage of the intervention is 100 percent, universal home fortification with micronutrient powders (MNPs) from age 6-12 months averted 32.3 [95 percent CIs = -12.3 to 76] DALYs per 10,000 children, compared with a control cohort. Of these averted DALYs, -2.53 [95 percent CIs = -41.1 to 35.5] were YLLs and 34.8 [95 percent CIs = 28.9 to 40.4] were YLDs.

When the effects of micronutrient powders on anaemia alone (excluding effects on diarrhoea and malaria) are considered, treatment with micronutrient powders caused a change in YLDs per 10,000 children of 35.9.

Treatment with micronutrient powders caused a net increase/decrease in healthcare costs of USD $10.1 per DALY averted.

If coverage was:
* 100 percent, net DALYs per 10,000 children averted was 32.3.
* 75 percent, net DALYs per 10,000 children averted was 22.6.
* 50 percent, net DALYs per 10,000 children averted was 14.3.
* 25 percent, net DALYs per 10,000 children averted was 6.09.

If coverage was:
* 100 percent: cost per DALY averted was $3136.
* 75 percent: cost per DALY averted was $4459.
* 50 percent: cost per DALY averted was $6990.
* 25 percent: cost per DALY averted was $16334.

Lao PDR (LAO)

--- INPUTS ---

Based on the 2017 Global Burden of Disease analysis, the incidence rates of diarrhoea are: mild = 1 events per year per child; moderate = 0.55 per year per child; severe = 0.008 per year per child.

Based on the 2017 Global Burden of Disease analysis, the incidence rates of malaria are: mild = 0.0016 per year per child; moderate = 0.00085 per year per child; severe = 1.2e-05 per year per child.

Based on the most recent Demographic and Health Survey (or equivalent), the prevalences of anaemia are:
6-8 months mild 23 percent, moderate 43 percent, severe 0 percent
9-11 months mild 22 percent, moderate 42 percent, severe 5.5 percent
12-17 months mild 23 percent, moderate 39 percent, severe 0 percent.

Background mortality rates through the treatment period are 1.14 percent per year on average.

Case-specific mortality rates are 0.0254 percent for a case of diarrhoea; 0.0451 percent for a case of malaria.

Lao PDR is classified as having endemic malaria, and has an Insecticide Treated bednet coverage rate of 43 percent based on 2015 UNICEF bednet coverage data.

In this country, the cost of an outpatient clinic visit is $1.75, and of hospitalisation is $7.25. We modelled the cost of MNPs as US $9.9 per course (including implementation).

--- OUTPUTS ---

Treatment with micronutrient powders caused a 5 percent increase in the incidence of diarrhoea, and a 2 percent increase in the incidence of malaria.
Treatment with micronutrient powders produced a 18 percent absolute decrease in the prevalence of anaemia at 12 months of age, and a 26 percent relative decrease.

Treatment with micronutrient powders caused an additional 0.279 deaths per 10,000 children [95 percent CIs = 1.31 to -0.529].

If coverage of the intervention is 100 percent, universal home fortification with micronutrient powders (MNPs) from age 6-12 months averted 42.2 [95 percent CIs = -31.1 to 100] DALYs per 10,000 children, compared with a control cohort. Of these averted DALYs, -18.1 [95 percent CIs = -84.9 to 34.4] were YLLs and 60.3 [95 percent CIs = 53.7 to 65.9] were YLDs.

When the effects of micronutrient powders on anaemia alone (excluding effects on diarrhoea and malaria) are considered, treatment with micronutrient powders caused a change in YLDs per 10,000 children of 61.7.

Treatment with micronutrient powders caused a net increase/decrease in healthcare costs of USD $10.1 per DALY averted.

If coverage was:
* 100 percent, net DALYs per 10,000 children averted was 42.2.
* 75 percent, net DALYs per 10,000 children averted was 31.6.
* 50 percent, net DALYs per 10,000 children averted was 20.9.
* 25 percent, net DALYs per 10,000 children averted was 10.4.

If coverage was:
* 100 percent: cost per DALY averted was $2389.
* 75 percent: cost per DALY averted was $3178.
* 50 percent: cost per DALY averted was $4780.
* 25 percent: cost per DALY averted was $9535.

Cambodia (KHM)

--- INPUTS ---

Based on the 2017 Global Burden of Disease analysis, the incidence rates of diarrhoea are: mild = 1.1 events per year per child; moderate = 0.61 per year per child; severe = 0.0088 per year per child.

Based on the 2017 Global Burden of Disease analysis, the incidence rates of malaria are: mild = 0.0008 per year per child; moderate = 0.00043 per year per child; severe = 6.2e-06 per year per child.

Based on the most recent Demographic and Health Survey (or equivalent), the prevalences of anaemia are:
6-8 months mild 30 percent, moderate 46 percent, severe 0.6 percent
9-11 months mild 31 percent, moderate 51 percent, severe 0.7 percent
12-17 months mild 29 percent, moderate 46 percent, severe 1.5 percent.

Background mortality rates through the treatment period are 0.484 percent per year on average.

Case-specific mortality rates are 0.00282 percent for a case of diarrhoea; 4.68 percent for a case of malaria.

Cambodia is classified as having endemic malaria, and has an Insecticide Treated bednet coverage rate of 4 percent based on 2015 UNICEF bednet coverage data.

In this country, the cost of an outpatient clinic visit is $1.3, and of hospitalisation is $5.28. We modelled the cost of MNPs as US $9.9 per course (including implementation).

--- OUTPUTS ---

Treatment with micronutrient powders caused a 5 percent increase in the incidence of diarrhoea, and a 7 percent increase in the incidence of malaria.
Treatment with micronutrient powders produced a 20 percent absolute decrease in the prevalence of anaemia at 12 months of age, and a 24 percent relative decrease.

Treatment with micronutrient powders caused an additional 0.059 deaths per 10,000 children [95 percent CIs = 0.274 to -0.124].

If coverage of the intervention is 100 percent, universal home fortification with micronutrient powders (MNPs) from age 6-12 months averted 58 [95 percent CIs = 36.9 to 76.5] DALYs per 10,000 children, compared with a control cohort. Of these averted DALYs, -4.01 [95 percent CIs = -18.6 to 8.46] were YLLs and 62 [95 percent CIs = 55.5 to 68.1] were YLDs.

When the effects of micronutrient powders on anaemia alone (excluding effects on diarrhoea and malaria) are considered, treatment with micronutrient powders caused a change in YLDs per 10,000 children of 63.5.

Treatment with micronutrient powders caused a net increase/decrease in healthcare costs of USD $10 per DALY averted.

If coverage was:
* 100 percent, net DALYs per 10,000 children averted was 58.
* 75 percent, net DALYs per 10,000 children averted was 43.3.
* 50 percent, net DALYs per 10,000 children averted was 28.9.
* 25 percent, net DALYs per 10,000 children averted was 14.4.

If coverage was:
* 100 percent: cost per DALY averted was $1729.
* 75 percent: cost per DALY averted was $2307.
* 50 percent: cost per DALY averted was $3447.
* 25 percent: cost per DALY averted was $6878.

Uzbekistan (UZB)

--- INPUTS ---

Based on the 2017 Global Burden of Disease analysis, the incidence rates of diarrhoea are: mild = 0.42 events per year per child; moderate = 0.22 per year per child; severe = 0.0032 per year per child.

Based on the 2017 Global Burden of Disease analysis, the incidence rates of malaria are: mild = 0 per year per child; moderate = 0 per year per child; severe = 0 per year per child.

Based on the most recent Demographic and Health Survey (or equivalent), the prevalences of anaemia are:
6-8 months mild 26 percent, moderate 25 percent, severe 0 percent
9-11 months mild 37 percent, moderate 22 percent, severe 0 percent
12-17 months mild 25 percent, moderate 42 percent, severe 2.6 percent.

Background mortality rates through the treatment period are 0.639 percent per year on average.

Case-specific mortality rates are 0.00207 percent for a case of diarrhoea; NaN percent for a case of malaria.

Uzbekistan is not classified as having endemic malaria, and has an Insecticide Treated bednet coverage rate of 0 percent based on 2015 UNICEF bednet coverage data.

In this country, the cost of an outpatient clinic visit is $1.66, and of hospitalisation is $7.43. We modelled the cost of MNPs as US $9.9 per course (including implementation).

--- OUTPUTS ---

Treatment with micronutrient powders caused a 4 percent increase in the incidence of diarrhoea, and a NaN percent decrease in the incidence of malaria.
Treatment with micronutrient powders produced a 14 percent absolute decrease in the prevalence of anaemia at 12 months of age, and a 24 percent relative decrease.

Treatment with micronutrient powders caused an additional 0.0133 deaths per 10,000 children [95 percent CIs = 0.0732 to -0.0335].

If coverage of the intervention is 100 percent, universal home fortification with micronutrient powders (MNPs) from age 6-12 months averted 43.6 [95 percent CIs = 34.7 to 51] DALYs per 10,000 children, compared with a control cohort. Of these averted DALYs, -0.912 [95 percent CIs = -5.03 to 2.3] were YLLs and 44.5 [95 percent CIs = 39.8 to 48.7] were YLDs.

When the effects of micronutrient powders on anaemia alone (excluding effects on diarrhoea and malaria) are considered, treatment with micronutrient powders caused a change in YLDs per 10,000 children of 45.

Treatment with micronutrient powders caused a net increase/decrease in healthcare costs of USD $9.96 per DALY averted.

If coverage was:

* 100 percent, net DALYs per 10,000 children averted was 43.6.
* 75 percent, net DALYs per 10,000 children averted was 32.7.
* 50 percent, net DALYs per 10,000 children averted was 21.8.
* 25 percent, net DALYs per 10,000 children averted was 10.9.

If coverage was:
* 100 percent: cost per DALY averted was $2283.
* 75 percent: cost per DALY averted was $3041.
* 50 percent: cost per DALY averted was $4557.
* 25 percent: cost per DALY averted was $9099.

Papua New Guinea (PNG)

--- INPUTS ---

Based on the 2017 Global Burden of Disease analysis, the incidence rates of diarrhoea are: mild = 1.7 events per year per child; moderate = 0.93 per year per child; severe = 0.013 per year per child.

Based on the 2017 Global Burden of Disease analysis, the incidence rates of malaria are: mild = 0.099 per year per child; moderate = 0.053 per year per child; severe = 0.00076 per year per child.

Based on the most recent Demographic and Health Survey (or equivalent), the prevalences of anaemia are:
6-8 months mild 39 percent, moderate 35 percent, severe 0 percent
9-11 months mild 25 percent, moderate 35 percent, severe 4.2 percent
12-17 months mild 25 percent, moderate 39 percent, severe 4.1 percent.

Background mortality rates through the treatment period are 0.975 percent per year on average.

Case-specific mortality rates are 0.0236 percent for a case of diarrhoea; 0.0258 percent for a case of malaria.

Papua New Guinea is classified as having endemic malaria, and has an Insecticide Treated bednet coverage rate of 58.2 percent based on 2015 UNICEF bednet coverage data.

In this country, the cost of an outpatient clinic visit is $2.69, and of hospitalisation is $11.4. We modelled the cost of MNPs as US $9.9 per course (including implementation).

--- OUTPUTS ---

Treatment with micronutrient powders caused a 5 percent increase in the incidence of diarrhoea, and a 3 percent increase in the incidence of malaria.
Treatment with micronutrient powders produced a 15 percent absolute decrease in the prevalence of anaemia at 12 months of age, and a 25 percent relative decrease.

Treatment with micronutrient powders caused an additional 0.453 deaths per 10,000 children [95 percent CIs = 1.93 to -0.821].

If coverage of the intervention is 100 percent, universal home fortification with micronutrient powders (MNPs) from age 6-12 months averted 27.3 [95 percent CIs = -73.6 to 115] DALYs per 10,000 children, compared with a control cohort. Of these averted DALYs, -28.2 [95 percent CIs = -120 to 51] were YLLs and 55.5 [95 percent CIs = 46.3 to 64.1] were YLDs.

When the effects of micronutrient powders on anaemia alone (excluding effects on diarrhoea and malaria) are considered, treatment with micronutrient powders caused a change in YLDs per 10,000 children of 57.9.

Treatment with micronutrient powders caused a net increase/decrease in healthcare costs of USD $10.3 per DALY averted.

If coverage was:
* 100 percent, net DALYs per 10,000 children averted was 27.3.
* 75 percent, net DALYs per 10,000 children averted was 20.5.
* 50 percent, net DALYs per 10,000 children averted was 13.6.
* 25 percent, net DALYs per 10,000 children averted was 6.71.

If coverage was:
* 100 percent: cost per DALY averted was $3779.
* 75 percent: cost per DALY averted was $4988.
* 50 percent: cost per DALY averted was $7456.
* 25 percent: cost per DALY averted was $14913.

Latin America

Grenada (GRD)

--- INPUTS ---

Based on the 2017 Global Burden of Disease analysis, the incidence rates of diarrhoea are: mild = 0.98 events per year per child; moderate = 0.52 per year per child; severe = 0.0076 per year per child.

Based on the 2017 Global Burden of Disease analysis, the incidence rates of malaria are: mild = 0 per year per child; moderate = 0 per year per child; severe = 0 per year per child.

Based on the most recent Demographic and Health Survey (or equivalent), the prevalences of anaemia are:
6-8 months mild 38 percent, moderate 26 percent, severe 0 percent
9-11 months mild 50 percent, moderate 33 percent, severe 0.34 percent
12-17 months mild 24 percent, moderate 40 percent, severe 0.56 percent.

Background mortality rates through the treatment period are 0.743 percent per year on average.

Case-specific mortality rates are 0.000488 percent for a case of diarrhoea; NaN percent for a case of malaria.

Grenada is not classified as having endemic malaria, and has an Insecticide Treated bednet coverage rate of 0 percent based on 2015 UNICEF bednet coverage data.

In this country, the cost of an outpatient clinic visit is $9.67, and of hospitalisation is $65.4. We modelled the cost of MNPs as US $9.9 per course (including implementation).

--- OUTPUTS ---

Treatment with micronutrient powders caused a 4 percent increase in the incidence of diarrhoea, and a NaN percent decrease in the incidence of malaria.
Treatment with micronutrient powders produced a 18 percent absolute decrease in the prevalence of anaemia at 12 months of age, and a 22 percent relative decrease.

Treatment with micronutrient powders caused an additional 0.0212 deaths per 10,000 children [95 percent CIs = 0.103 to -0.0535].

If coverage of the intervention is 100 percent, universal home fortification with micronutrient powders (MNPs) from age 6-12 months averted 37.3 [95 percent CIs = 25.5 to 48.6] DALYs per 10,000 children, compared with a control cohort. Of these averted DALYs, -1.54 [95 percent CIs = -7.52 to 3.9] were YLLs and 38.9 [95 percent CIs = 33 to 44.7] were YLDs.

When the effects of micronutrient powders on anaemia alone (excluding effects on diarrhoea and malaria) are considered, treatment with micronutrient powders caused a change in YLDs per 10,000 children of 40.

Treatment with micronutrient powders caused a net increase/decrease in healthcare costs of USD $10.8 per DALY averted.

If coverage was:
* 100 percent, net DALYs per 10,000 children averted was 37.3.
* 75 percent, net DALYs per 10,000 children averted was 28.
* 50 percent, net DALYs per 10,000 children averted was 18.8.
* 25 percent, net DALYs per 10,000 children averted was 9.35.

If coverage was:
* 100 percent: cost per DALY averted was $2880.
* 75 percent: cost per DALY averted was $3769.
* 50 percent: cost per DALY averted was $5515.
* 25 percent: cost per DALY averted was $10781.

Colombia (COL)

--- INPUTS ---

Based on the 2017 Global Burden of Disease analysis, the incidence rates of diarrhoea are: mild = 1.1 events per year per child; moderate = 0.61 per year per child; severe = 0.0088 per year per child.

Based on the 2017 Global Burden of Disease analysis, the incidence rates of malaria are: mild = 0.00048 per year per child; moderate = 0.00026 per year per child; severe = 3.7e-06 per year per child.

Based on the most recent Demographic and Health Survey (or equivalent), the prevalences of anaemia are:
6-8 months mild 40 percent, moderate 20 percent, severe 0.073 percent
9-11 months mild 36 percent, moderate 22 percent, severe 0.14 percent
12-17 months mild 24 percent, moderate 12 percent, severe 0 percent.

Background mortality rates through the treatment period are 0.91 percent per year on average.

Case-specific mortality rates are 0.00154 percent for a case of diarrhoea; 0.128 percent for a case of malaria.

Colombia is not classified as having endemic malaria, and has an Insecticide Treated bednet coverage rate of 0 percent based on 2015 UNICEF bednet coverage data.

In this country, the cost of an outpatient clinic visit is $10.4, and of hospitalisation is $37.3. We modelled the cost of MNPs as US $9.9 per course (including implementation).

--- OUTPUTS ---

Treatment with micronutrient powders caused a 5 percent increase in the incidence of diarrhoea, and a 2 percent decrease in the incidence of malaria.
Treatment with micronutrient powders produced a 15 percent absolute decrease in the prevalence of anaemia at 12 months of age, and a 25 percent relative decrease.

Treatment with micronutrient powders caused an additional 0.0416 deaths per 10,000 children [95 percent CIs = 0.196 to -0.0786].

If coverage of the intervention is 100 percent, universal home fortification with micronutrient powders (MNPs) from age 6-12 months averted 18.1 [95 percent CIs = 1.43 to 32.4] DALYs per 10,000 children, compared with a control cohort. Of these averted DALYs, -3.08 [95 percent CIs = -14.5 to 5.82] were YLLs and 21.2 [95 percent CIs = 15.9 to 26.6] were YLDs.

When the effects of micronutrient powders on anaemia alone (excluding effects on diarrhoea and malaria) are considered, treatment with micronutrient powders caused a change in YLDs per 10,000 children of 22.6.

Treatment with micronutrient powders caused a net increase/decrease in healthcare costs of USD $10.9 per DALY averted.

If coverage was:
* 100 percent, net DALYs per 10,000 children averted was 18.1.
* 75 percent, net DALYs per 10,000 children averted was 13.6.
* 50 percent, net DALYs per 10,000 children averted was 9.01.
* 25 percent, net DALYs per 10,000 children averted was 4.51.

If coverage was:
* 100 percent: cost per DALY averted was $6026.
* 75 percent: cost per DALY averted was $7849.
* 50 percent: cost per DALY averted was $11520.
* 25 percent: cost per DALY averted was $22525.

Cuba (CUB)

--- INPUTS ---

Based on the 2017 Global Burden of Disease analysis, the incidence rates of diarrhoea are: mild = 0.51 events per year per child; moderate = 0.27 per year per child; severe = 0.004 per year per child.

Based on the 2017 Global Burden of Disease analysis, the incidence rates of malaria are: mild = 0 per year per child; moderate = 0 per year per child; severe = 0 per year per child.

Based on the most recent Demographic and Health Survey (or equivalent), the prevalences of anaemia are:
6-8 months mild 28 percent, moderate 19 percent, severe 0 percent
9-11 months mild 37 percent, moderate 24 percent, severe 0.25 percent
12-17 months mild 18 percent, moderate 30 percent, severe 0.41 percent.

Background mortality rates through the treatment period are 0.279 percent per year on average.

Case-specific mortality rates are 0.000448 percent for a case of diarrhoea; NaN percent for a case of malaria.

Cuba is not classified as having endemic malaria, and has an Insecticide Treated bednet coverage rate of 0 percent based on 2015 UNICEF bednet coverage data.

In this country, the cost of an outpatient clinic visit is $NaN, and of hospitalisation is $NaN. We modelled the cost of MNPs as US $9.9 per course (including implementation).

--- OUTPUTS ---

Treatment with micronutrient powders caused a 5 percent increase in the incidence of diarrhoea, and a NaN percent decrease in the incidence of malaria.
Treatment with micronutrient powders produced a 15 percent absolute decrease in the prevalence of anaemia at 12 months of age, and a 23 percent relative decrease.

Treatment with micronutrient powders caused an additional 0.00461 deaths per 10,000 children [95 percent CIs = 0.0236 to -0.0103].

If coverage of the intervention is 100 percent, universal home fortification with micronutrient powders (MNPs) from age 6-12 months averted 30.2 [95 percent CIs = 24.3 to 35.3] DALYs per 10,000 children, compared with a control cohort. Of these averted DALYs, -0.361 [95 percent CIs = -1.85 to 0.807] were YLLs and 30.5 [95 percent CIs = 26.2 to 34.5] were YLDs.

When the effects of micronutrient powders on anaemia alone (excluding effects on diarrhoea and malaria) are considered, treatment with micronutrient powders caused a change in YLDs per 10,000 children of 31.2.

Treatment with micronutrient powders caused a net increase/decrease in healthcare costs of USD $NaN per DALY averted.

If coverage was:
* 100 percent, net DALYs per 10,000 children averted was 30.2.
* 75 percent, net DALYs per 10,000 children averted was 22.7.
* 50 percent, net DALYs per 10,000 children averted was 15.1.
* 25 percent, net DALYs per 10,000 children averted was 7.5.

If coverage was:
* 100 percent: cost per DALY averted was $NaN.
* 75 percent: cost per DALY averted was $NaN.
* 50 percent: cost per DALY averted was $NaN.
* 25 percent: cost per DALY averted was $NaN.

Dominican Republic (DOM)

--- INPUTS ---

Based on the 2017 Global Burden of Disease analysis, the incidence rates of diarrhoea are: mild = 1.4 events per year per child; moderate = 0.75 per year per child; severe = 0.011 per year per child.

Based on the 2017 Global Burden of Disease analysis, the incidence rates of malaria are: mild = 2.4e-05 per year per child; moderate = 1.3e-05 per year per child; severe = 1.8e-07 per year per child.

Based on the most recent Demographic and Health Survey (or equivalent), the prevalences of anaemia are:
6-8 months mild 27 percent, moderate 19 percent, severe 0 percent
9-11 months mild 36 percent, moderate 24 percent, severe 0.24 percent
12-17 months mild 17 percent, moderate 29 percent, severe 0.4 percent.

Background mortality rates through the treatment period are 1.75 percent per year on average.

Case-specific mortality rates are 0.00231 percent for a case of diarrhoea; 0.0155 percent for a case of malaria.

Dominican Republic is not classified as having endemic malaria, and has an Insecticide Treated bednet coverage rate of 0 percent based on 2015 UNICEF bednet coverage data.

In this country, the cost of an outpatient clinic visit is $6.41, and of hospitalisation is $41.7. We modelled the cost of MNPs as US $9.9 per course (including implementation).

--- OUTPUTS ---

Treatment with micronutrient powders caused a 5 percent increase in the incidence of diarrhoea, and a 0.8 percent decrease in the incidence of malaria.
Treatment with micronutrient powders produced a 16 percent absolute decrease in the prevalence of anaemia at 12 months of age, and a 26 percent relative decrease.

Treatment with micronutrient powders caused an additional 0.0752 deaths per 10,000 children [95 percent CIs = 0.34 to -0.131].

If coverage of the intervention is 100 percent, universal home fortification with micronutrient powders (MNPs) from age 6-12 months averted 24.7 [95 percent CIs = -2.25 to 46] DALYs per 10,000 children, compared with a control cohort. Of these averted DALYs, -5.5 [95 percent CIs = -24.9 to 9.61] were YLLs and 30.2 [95 percent CIs = 22.6 to 36.4] were YLDs.

When the effects of micronutrient powders on anaemia alone (excluding effects on diarrhoea and malaria) are considered, treatment with micronutrient powders caused a change in YLDs per 10,000 children of 32.1.

Treatment with micronutrient powders caused a net increase/decrease in healthcare costs of USD $10.8 per DALY averted.

If coverage was:
* 100 percent, net DALYs per 10,000 children averted was 24.7.
* 75 percent, net DALYs per 10,000 children averted was 18.5.
* 50 percent, net DALYs per 10,000 children averted was 12.4.
* 25 percent, net DALYs per 10,000 children averted was 6.11.

If coverage was:
* 100 percent: cost per DALY averted was $4381.
* 75 percent: cost per DALY averted was $5709.
* 50 percent: cost per DALY averted was $8338.
* 25 percent: cost per DALY averted was $16542.

Peru (PER)

--- INPUTS ---

Based on the 2017 Global Burden of Disease analysis, the incidence rates of diarrhoea are: mild = 1.3 events per year per child; moderate = 0.67 per year per child; severe = 0.0097 per year per child.

Based on the 2017 Global Burden of Disease analysis, the incidence rates of malaria are: mild = 0.00078 per year per child; moderate = 0.00042 per year per child; severe = 6e-06 per year per child.

Based on the most recent Demographic and Health Survey (or equivalent), the prevalences of anaemia are:
6-8 months mild 33 percent, moderate 27 percent, severe 1.2 percent
9-11 months mild 30 percent, moderate 33 percent, severe 0.1 percent
12-17 months mild 35 percent, moderate 27 percent, severe 1.1 percent.

Background mortality rates through the treatment period are 0.897 percent per year on average.

Case-specific mortality rates are 0.00173 percent for a case of diarrhoea; 0.00589 percent for a case of malaria.

Peru is classified as having endemic malaria, and has an Insecticide Treated bednet coverage rate of 0 percent based on 2015 UNICEF bednet coverage data.

In this country, the cost of an outpatient clinic visit is $6.46, and of hospitalisation is $42.1. We modelled the cost of MNPs as US $9.9 per course (including implementation).

--- OUTPUTS ---

Treatment with micronutrient powders caused a 5 percent increase in the incidence of diarrhoea, and a 5 percent increase in the incidence of malaria.
Treatment with micronutrient powders produced a 17 percent absolute decrease in the prevalence of anaemia at 12 months of age, and a 27 percent relative decrease.

Treatment with micronutrient powders caused an additional 0.0361 deaths per 10,000 children [95 percent CIs = 0.169 to -0.071].

If coverage of the intervention is 100 percent, universal home fortification with micronutrient powders (MNPs) from age 6-12 months averted 39.6 [95 percent CIs = 22.9 to 54.1] DALYs per 10,000 children, compared with a control cohort. Of these averted DALYs, -2.7 [95 percent CIs = -12.7 to 5.31] were YLLs and 42.3 [95 percent CIs = 35.6 to 48.8] were YLDs.

When the effects of micronutrient powders on anaemia alone (excluding effects on diarrhoea and malaria) are considered, treatment with micronutrient powders caused a change in YLDs per 10,000 children of 44.

Treatment with micronutrient powders caused a net increase/decrease in healthcare costs of USD $10.7 per DALY averted.

If coverage was:
* 100 percent, net DALYs per 10,000 children averted was 39.6.
* 75 percent, net DALYs per 10,000 children averted was 29.7.
* 50 percent, net DALYs per 10,000 children averted was 19.8.
* 25 percent, net DALYs per 10,000 children averted was 9.93.

If coverage was:
* 100 percent: cost per DALY averted was $2689.
* 75 percent: cost per DALY averted was $3517.
* 50 percent: cost per DALY averted was $5186.
* 25 percent: cost per DALY averted was $10165.

Uruguay (URY)

--- INPUTS ---

Based on the 2017 Global Burden of Disease analysis, the incidence rates of diarrhoea are: mild = 1 events per year per child; moderate = 0.56 per year per child; severe = 0.008 per year per child.

Based on the 2017 Global Burden of Disease analysis, the incidence rates of malaria are: mild = 0 per year per child; moderate = 0 per year per child; severe = 0 per year per child.

Based on the most recent Demographic and Health Survey (or equivalent), the prevalences of anaemia are:
6-8 months mild 24 percent, moderate 19 percent, severe 0.87 percent
9-11 months mild 22 percent, moderate 24 percent, severe 0.073 percent
12-17 months mild 25 percent, moderate 20 percent, severe 0.8 percent.

Background mortality rates through the treatment period are 0.613 percent per year on average.

Case-specific mortality rates are 0.000519 percent for a case of diarrhoea; NaN percent for a case of malaria.

Uruguay is not classified as having endemic malaria, and has an Insecticide Treated bednet coverage rate of 0 percent based on 2015 UNICEF bednet coverage data.

In this country, the cost of an outpatient clinic visit is $14.1, and of hospitalisation is $104. We modelled the cost of MNPs as US $9.9 per course (including implementation).

--- OUTPUTS ---

Treatment with micronutrient powders caused a 5 percent increase in the incidence of diarrhoea, and a NaN percent decrease in the incidence of malaria.
Treatment with micronutrient powders produced a 11 percent absolute decrease in the prevalence of anaemia at 12 months of age, and a 24 percent relative decrease.

Treatment with micronutrient powders caused an additional 0.172 deaths per 10,000 children [95 percent CIs = 0.8 to -0.377].

If coverage of the intervention is 100 percent, universal home fortification with micronutrient powders (MNPs) from age 6-12 months averted 14.6 [95 percent CIs = -39.6 to 62.2] DALYs per 10,000 children, compared with a control cohort. Of these averted DALYs, -13.1 [95 percent CIs = -61 to 28.8] were YLLs and 27.7 [95 percent CIs = 21.4 to 33.4] were YLDs.

When the effects of micronutrient powders on anaemia alone (excluding effects on diarrhoea and malaria) are considered, treatment with micronutrient powders caused a change in YLDs per 10,000 children of 29.

Treatment with micronutrient powders caused a net increase/decrease in healthcare costs of USD $11.1 per DALY averted.

If coverage was:
* 100 percent, net DALYs per 10,000 children averted was 14.6.
* 75 percent, net DALYs per 10,000 children averted was 10.9.
* 50 percent, net DALYs per 10,000 children averted was 7.3.
* 25 percent, net DALYs per 10,000 children averted was 3.62.

If coverage was:
* 100 percent: cost per DALY averted was $7626.
* 75 percent: cost per DALY averted was $9885.
* 50 percent: cost per DALY averted was $14354.
* 25 percent: cost per DALY averted was $28286.

Ecuador (ECU)

--- INPUTS ---

Based on the 2017 Global Burden of Disease analysis, the incidence rates of diarrhoea are: mild = 1 events per year per child; moderate = 0.53 per year per child; severe = 0.0077 per year per child.

Based on the 2017 Global Burden of Disease analysis, the incidence rates of malaria are: mild = 3.7e-05 per year per child; moderate = 2e-05 per year per child; severe = 2.9e-07 per year per child.

Based on the most recent Demographic and Health Survey (or equivalent), the prevalences of anaemia are:
6-8 months mild 34 percent, moderate 29 percent, severe 0.9 percent
9-11 months mild 34 percent, moderate 29 percent, severe 0.9 percent
12-17 months mild 25 percent, moderate 14 percent, severe 0.5 percent.

Background mortality rates through the treatment period are 1.26 percent per year on average.

Case-specific mortality rates are 0.0035 percent for a case of diarrhoea; 0.247 percent for a case of malaria.

Ecuador is not classified as having endemic malaria, and has an Insecticide Treated bednet coverage rate of 0 percent based on 2015 UNICEF bednet coverage data.

In this country, the cost of an outpatient clinic visit is $0.000285, and of hospitalisation is $0.00185. We modelled the cost of MNPs as US $9.9 per course (including implementation).

--- OUTPUTS ---

Treatment with micronutrient powders caused a 5 percent increase in the incidence of diarrhoea, and a 2e+01 percent increase in the incidence of malaria.
Treatment with micronutrient powders produced a 13 percent absolute decrease in the prevalence of anaemia at 12 months of age, and a 20 percent relative decrease.

Treatment with micronutrient powders caused an additional 0.0549 deaths per 10,000 children [95 percent CIs = 0.253 to -0.111].

If coverage of the intervention is 100 percent, universal home fortification with micronutrient powders (MNPs) from age 6-12 months averted 20.1 [95 percent CIs = -0.303 to 37.9] DALYs per 10,000 children, compared with a control cohort. Of these averted DALYs, -4.15 [95 percent CIs = -19.1 to 8.38] were YLLs and 24.3 [95 percent CIs = 18.8 to 29.5] were YLDs.

When the effects of micronutrient powders on anaemia alone (excluding effects on diarrhoea and malaria) are considered, treatment with micronutrient powders caused a change in YLDs per 10,000 children of 25.6.

Treatment with micronutrient powders caused a net increase/decrease in healthcare costs of USD $9.9 per DALY averted.

If coverage was:
* 100 percent, net DALYs per 10,000 children averted was 20.1.
* 75 percent, net DALYs per 10,000 children averted was 15.
* 50 percent, net DALYs per 10,000 children averted was 9.95.
* 25 percent, net DALYs per 10,000 children averted was 4.94.

If coverage was:
* 100 percent: cost per DALY averted was $4918.
* 75 percent: cost per DALY averted was $6607.
* 50 percent: cost per DALY averted was $9947.
* 25 percent: cost per DALY averted was $20043.

Bolivia (BOL)

--- INPUTS ---

Based on the 2017 Global Burden of Disease analysis, the incidence rates of diarrhoea are: mild = 1.3 events per year per child; moderate = 0.71 per year per child; severe = 0.01 per year per child.

Based on the 2017 Global Burden of Disease analysis, the incidence rates of malaria are: mild = 0.00022 per year per child; moderate = 0.00012 per year per child; severe = 1.7e-06 per year per child.

Based on the most recent Demographic and Health Survey (or equivalent), the prevalences of anaemia are:
6-8 months mild 25 percent, moderate 41 percent, severe 12 percent
9-11 months mild 18 percent, moderate 54 percent, severe 5.5 percent
12-17 months mild 20 percent, moderate 55 percent, severe 7.6 percent.

Background mortality rates through the treatment period are 2.11 percent per year on average.

Case-specific mortality rates are 0.00341 percent for a case of diarrhoea; 0.065 percent for a case of malaria.

Bolivia is classified as having endemic malaria, and has an Insecticide Treated bednet coverage rate of 0 percent based on 2015 UNICEF bednet coverage data.

In this country, the cost of an outpatient clinic visit is $3.71, and of hospitalisation is $19.6. We modelled the cost of MNPs as US $9.9 per course (including implementation).

--- OUTPUTS ---

Treatment with micronutrient powders caused a 5 percent increase in the incidence of diarrhoea, and a 4 percent increase in the incidence of malaria.
Treatment with micronutrient powders produced a 17 percent absolute decrease in the prevalence of anaemia at 12 months of age, and a 22 percent relative decrease.

Treatment with micronutrient powders caused an additional 0.133 deaths per 10,000 children [95 percent CIs = 0.573 to -0.241].

If coverage of the intervention is 100 percent, universal home fortification with micronutrient powders (MNPs) from age 6-12 months averted 74.3 [95 percent CIs = 33.5 to 109] DALYs per 10,000 children, compared with a control cohort. Of these averted DALYs, -9.31 [95 percent CIs = -40.1 to 16.9] were YLLs and 83.6 [95 percent CIs = 73.6 to 91.8] were YLDs.

When the effects of micronutrient powders on anaemia alone (excluding effects on diarrhoea and malaria) are considered, treatment with micronutrient powders caused a change in YLDs per 10,000 children of 85.6.

Treatment with micronutrient powders caused a net increase/decrease in healthcare costs of USD $10.4 per DALY averted.

If coverage was:

* 100 percent, net DALYs per 10,000 children averted was 74.3.
* 75 percent, net DALYs per 10,000 children averted was 55.7.
* 50 percent, net DALYs per 10,000 children averted was 37.1.
* 25 percent, net DALYs per 10,000 children averted was 18.5.

If coverage was:
* 100 percent: cost per DALY averted was $1398.
* 75 percent: cost per DALY averted was $1842.
* 50 percent: cost per DALY averted was $2731.
* 25 percent: cost per DALY averted was $5415.

Haiti (HTI)

--- INPUTS ---

Based on the 2017 Global Burden of Disease analysis, the incidence rates of diarrhoea are: mild = 1.4 events per year per child; moderate = 0.77 per year per child; severe = 0.011 per year per child.

Based on the 2017 Global Burden of Disease analysis, the incidence rates of malaria are: mild = 0.0037 per year per child; moderate = 0.002 per year per child; severe = 2.9e-05 per year per child.

Based on the most recent Demographic and Health Survey (or equivalent), the prevalences of anaemia are:
6-8 months mild 31 percent, moderate 53 percent, severe 2.4 percent
9-11 months mild 26 percent, moderate 59 percent, severe 2.1 percent
12-17 months mild 30 percent, moderate 47 percent, severe 1.2 percent.

Background mortality rates through the treatment period are 3.64 percent per year on average.

Case-specific mortality rates are 0.0268 percent for a case of diarrhoea; 0.00698 percent for a case of malaria.

Haiti is classified as having endemic malaria, and has an Insecticide Treated bednet coverage rate of 12 percent based on 2015 UNICEF bednet coverage data.

In this country, the cost of an outpatient clinic visit is $1.25, and of hospitalisation is $4.32. We modelled the cost of MNPs as US $9.9 per course (including implementation).

--- OUTPUTS ---

Treatment with micronutrient powders caused a 5 percent increase in the incidence of diarrhoea, and a 6 percent increase in the incidence of malaria.
Treatment with micronutrient powders produced a 22 percent absolute decrease in the prevalence of anaemia at 12 months of age, and a 24 percent relative decrease.

Treatment with micronutrient powders caused an additional 0.423 deaths per 10,000 children [95 percent CIs = 1.97 to -0.764].

If coverage of the intervention is 100 percent, universal home fortification with micronutrient powders (MNPs) from age 6-12 months averted 31.9 [95 percent CIs = -73 to 114] DALYs per 10,000 children, compared with a control cohort. Of these averted DALYs, -26.5 [95 percent CIs = -124 to 47.9] were YLLs and 58.4 [95 percent CIs = 50.6 to 65.7] were YLDs.

When the effects of micronutrient powders on anaemia alone (excluding effects on diarrhoea and malaria) are considered, treatment with micronutrient powders caused a change in YLDs per 10,000 children of 60.3.

Treatment with micronutrient powders caused a net increase/decrease in healthcare costs of USD $10.1 per DALY averted.

If coverage was:
* 100 percent, net DALYs per 10,000 children averted was 31.9.
* 75 percent, net DALYs per 10,000 children averted was 23.7.
* 50 percent, net DALYs per 10,000 children averted was 15.9.
* 25 percent, net DALYs per 10,000 children averted was 7.87.

If coverage was:
* 100 percent: cost per DALY averted was $3150.
* 75 percent: cost per DALY averted was $4218.
* 50 percent: cost per DALY averted was $6277.
* 25 percent: cost per DALY averted was $12632.

Guyana (GUY)

--- INPUTS ---

Based on the 2017 Global Burden of Disease analysis, the incidence rates of diarrhoea are: mild = 0.73 events per year per child; moderate = 0.39 per year per child; severe = 0.0056 per year per child.

Based on the 2017 Global Burden of Disease analysis, the incidence rates of malaria are: mild = 0.019 per year per child; moderate = 0.01 per year per child; severe = 0.00014 per year per child.

Based on the most recent Demographic and Health Survey (or equivalent), the prevalences of anaemia are:
6-8 months mild 34 percent, moderate 23 percent, severe 0 percent
9-11 months mild 44 percent, moderate 29 percent, severe 0.3 percent
12-17 months mild 21 percent, moderate 36 percent, severe 0.5 percent.

Background mortality rates through the treatment period are 2.21 percent per year on average.

Case-specific mortality rates are 0.00505 percent for a case of diarrhoea; 0.0223 percent for a case of malaria.

Guyana is classified as having endemic malaria, and has an Insecticide Treated bednet coverage rate of 24.4 percent based on 2015 UNICEF bednet coverage data.

In this country, the cost of an outpatient clinic visit is $0, and of hospitalisation is $0. We modelled the cost of MNPs as US $9.9 per course (including implementation).

--- OUTPUTS ---

Treatment with micronutrient powders caused a 5 percent increase in the incidence of diarrhoea, and a 6 percent increase in the incidence of malaria.
Treatment with micronutrient powders produced a 23 percent absolute decrease in the prevalence of anaemia at 12 months of age, and a 30 percent relative decrease.

Treatment with micronutrient powders caused an additional 0.0672 deaths per 10,000 children [95 percent CIs = 0.312 to -0.124].

If coverage of the intervention is 100 percent, universal home fortification with micronutrient powders (MNPs) from age 6-12 months averted 41.4 [95 percent CIs = 19.8 to 58.3] DALYs per 10,000 children, compared with a control cohort. Of these averted DALYs, -4.4 [95 percent CIs = -20.5 to 8.11] were YLLs and 45.8 [95 percent CIs = 40.3 to 50.1] were YLDs.

When the effects of micronutrient powders on anaemia alone (excluding effects on diarrhoea and malaria) are considered, treatment with micronutrient powders caused a change in YLDs per 10,000 children of 46.8.

Treatment with micronutrient powders caused a net increase/decrease in healthcare costs of USD $9.9 per DALY averted.

If coverage was:
* 100 percent, net DALYs per 10,000 children averted was 41.4.
* 75 percent, net DALYs per 10,000 children averted was 31.
* 50 percent, net DALYs per 10,000 children averted was 20.7.
* 25 percent, net DALYs per 10,000 children averted was 10.3.

If coverage was:
* 100 percent: cost per DALY averted was $2390.
* 75 percent: cost per DALY averted was $3190.
* 50 percent: cost per DALY averted was $4779.
* 25 percent: cost per DALY averted was $9631.

St. Lucia (LCA)

--- INPUTS ---

Based on the 2017 Global Burden of Disease analysis, the incidence rates of diarrhoea are: mild = 0.96 events per year per child; moderate = 0.51 per year per child; severe = 0.0074 per year per child.

Based on the 2017 Global Burden of Disease analysis, the incidence rates of malaria are: mild = 0 per year per child; moderate = 0 per year per child; severe = 0 per year per child.

Based on the most recent Demographic and Health Survey (or equivalent), the prevalences of anaemia are:
6-8 months mild 34 percent, moderate 23 percent, severe 0 percent
9-11 months mild 44 percent, moderate 29 percent, severe 0.3 percent
12-17 months mild 21 percent, moderate 36 percent, severe 0.5 percent.

Background mortality rates through the treatment period are 0.847 percent per year on average.

Case-specific mortality rates are 0.000727 percent for a case of diarrhoea; NaN percent for a case of malaria.

St. Lucia is not classified as having endemic malaria, and has an Insecticide Treated bednet coverage rate of 0 percent based on 2015 UNICEF bednet coverage data.

In this country, the cost of an outpatient clinic visit is $9.71, and of hospitalisation is $68.4. We modelled the cost of MNPs as US $9.9 per course (including implementation).

--- OUTPUTS ---

Treatment with micronutrient powders caused a 6 percent increase in the incidence of diarrhoea, and a NaN percent decrease in the incidence of malaria.
Treatment with micronutrient powders produced a 17 percent absolute decrease in the prevalence of anaemia at 12 months of age, and a 22 percent relative decrease.

Treatment with micronutrient powders caused an additional 0.0368 deaths per 10,000 children [95 percent CIs = 0.143 to -0.0552].

If coverage of the intervention is 100 percent, universal home fortification with micronutrient powders (MNPs) from age 6-12 months averted 33.9 [95 percent CIs = 20.6 to 46] DALYs per 10,000 children, compared with a control cohort. Of these averted DALYs, -2.74 [95 percent CIs = -10.6 to 4.11] were YLLs and 36.7 [95 percent CIs = 31.2 to 41.9] were YLDs.

When the effects of micronutrient powders on anaemia alone (excluding effects on diarrhoea and malaria) are considered, treatment with micronutrient powders caused a change in YLDs per 10,000 children of 38.2.

Treatment with micronutrient powders caused a net increase/decrease in healthcare costs of USD $11.1 per DALY averted.

If coverage was:
* 100 percent, net DALYs per 10,000 children averted was 33.9.
* 75 percent, net DALYs per 10,000 children averted was 25.4.
* 50 percent, net DALYs per 10,000 children averted was 16.9.
* 25 percent, net DALYs per 10,000 children averted was 8.49.

If coverage was:
* 100 percent: cost per DALY averted was $3283.
* 75 percent: cost per DALY averted was $4258.
* 50 percent: cost per DALY averted was $6230.

* 25 percent: cost per DALY averted was $12061.

Guatemala (GTM)

--- INPUTS ---

Based on the 2017 Global Burden of Disease analysis, the incidence rates of diarrhoea are: mild = 2 events per year per child; moderate = 1.1 per year per child; severe = 0.015 per year per child.

Based on the 2017 Global Burden of Disease analysis, the incidence rates of malaria are: mild = 0.00019 per year per child; moderate = 0.0001 per year per child; severe = 1.5e-06 per year per child.

Based on the most recent Demographic and Health Survey (or equivalent), the prevalences of anaemia are:
6-8 months mild 34 percent, moderate 29 percent, severe 0.9 percent
9-11 months mild 34 percent, moderate 29 percent, severe 0.9 percent
12-17 months mild 25 percent, moderate 14 percent, severe 0.5 percent.

Background mortality rates through the treatment period are 1.68 percent per year on average.

Case-specific mortality rates are 0.013 percent for a case of diarrhoea; 0.0649 percent for a case of malaria.

Guatemala is classified as having endemic malaria, and has an Insecticide Treated bednet coverage rate of 0 percent based on 2015 UNICEF bednet coverage data.

In this country, the cost of an outpatient clinic visit is $5.62, and of hospitalisation is $30.9. We modelled the cost of MNPs as US $9.9 per course (including implementation).

--- OUTPUTS ---

Treatment with micronutrient powders caused a 5 percent increase in the incidence of diarrhoea, and a 1e+01 percent increase in the incidence of malaria.
Treatment with micronutrient powders produced a 18 percent absolute decrease in the prevalence of anaemia at 12 months of age, and a 28 percent relative decrease.

Treatment with micronutrient powders caused an additional 0.508 deaths per 10,000 children [95 percent CIs = 2.42 to -0.888].

If coverage of the intervention is 100 percent, universal home fortification with micronutrient powders (MNPs) from age 6-12 months averted -4.16 [95 percent CIs = -150 to 103] DALYs per 10,000 children, compared with a control cohort. Of these averted DALYs, -36.1 [95 percent CIs = -172 to 63.2] were YLLs and 32 [95 percent CIs = 22.3 to 39.7] were YLDs.

When the effects of micronutrient powders on anaemia alone (excluding effects on diarrhoea and malaria) are considered, treatment with

micronutrient powders caused a change in YLDs per 10,000 children of 34.5.

Treatment with micronutrient powders caused a net increase/decrease in healthcare costs of USD $10.9 per DALY averted.

If coverage was:
* 100 percent, net DALYs per 10,000 children averted was -4.16.
* 75 percent, net DALYs per 10,000 children averted was -3.19.
* 50 percent, net DALYs per 10,000 children averted was -2.11.
* 25 percent, net DALYs per 10,000 children averted was -1.09.

[Because our model predicts that treatment with micronutrient powders increases net DALYs, we did not calculate the effect on healthcare costs when MNPs increase DALYs]

Figure S1 Anaemia-only model of effects of universal MNP interventions on DALYs

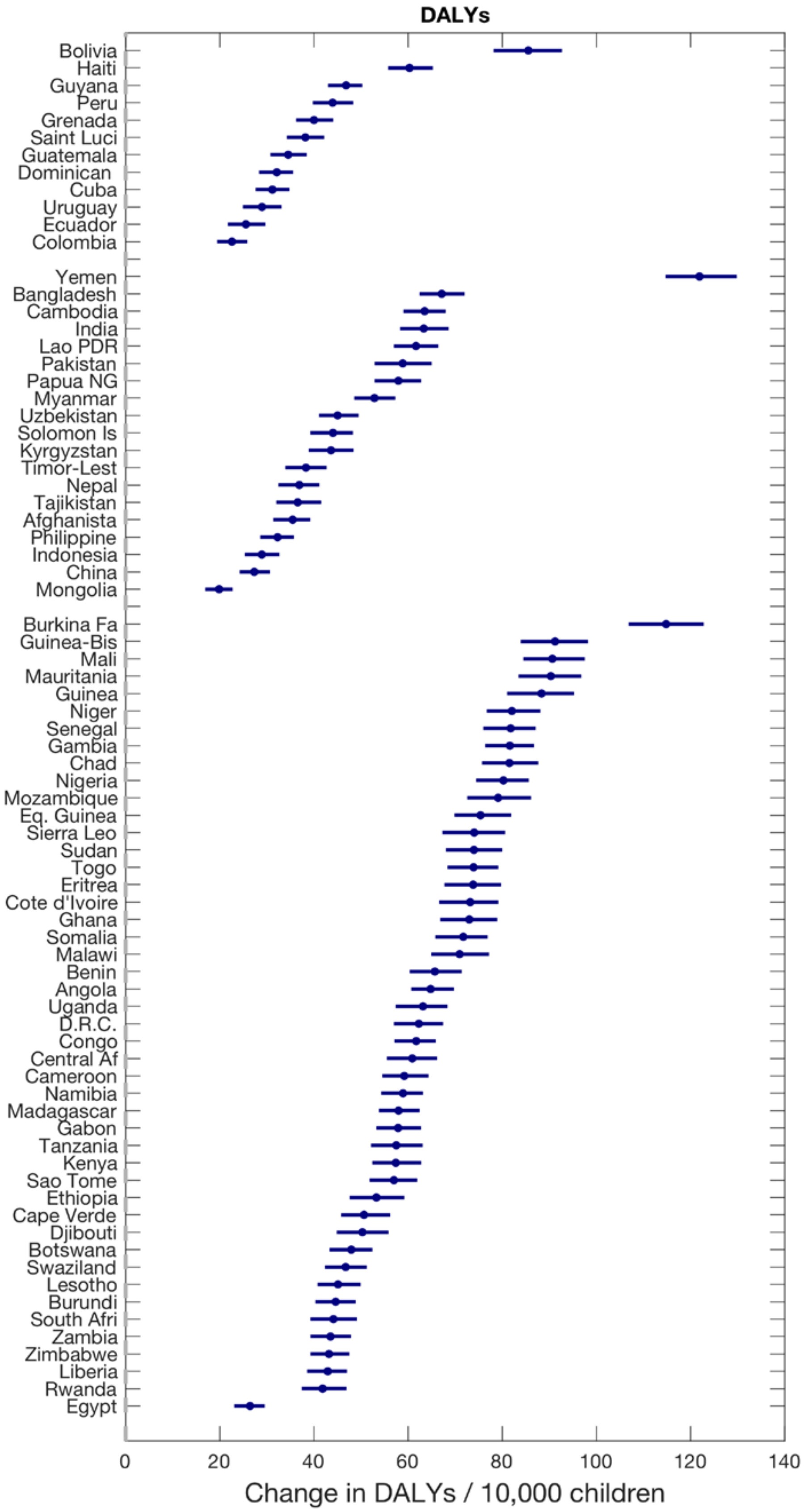

Figure S2 Cost per YLD averted for provision of MNPs adjusted by coverage

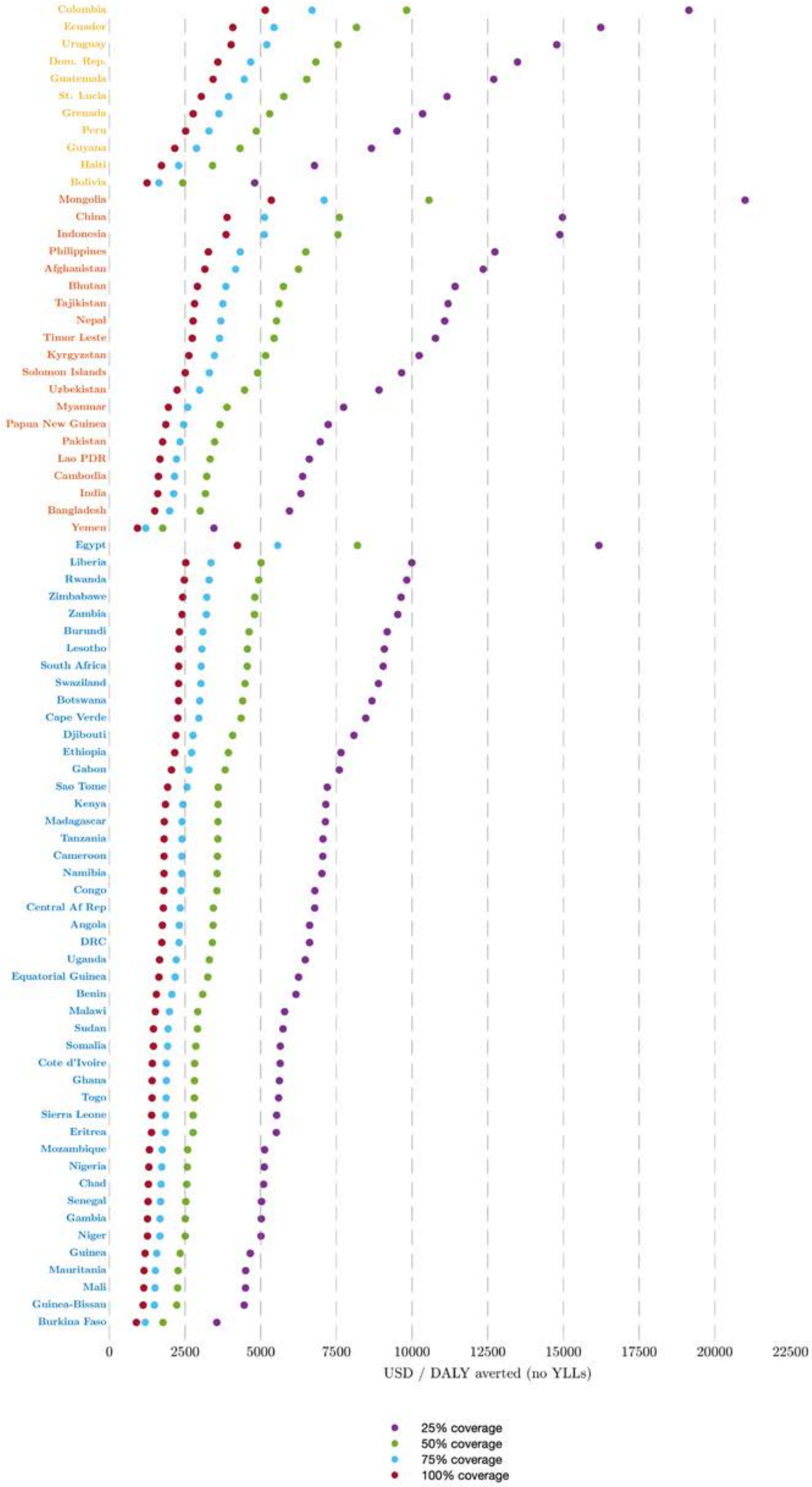

Figure S3 Cost Effectiveness Acceptability Curves for Africa

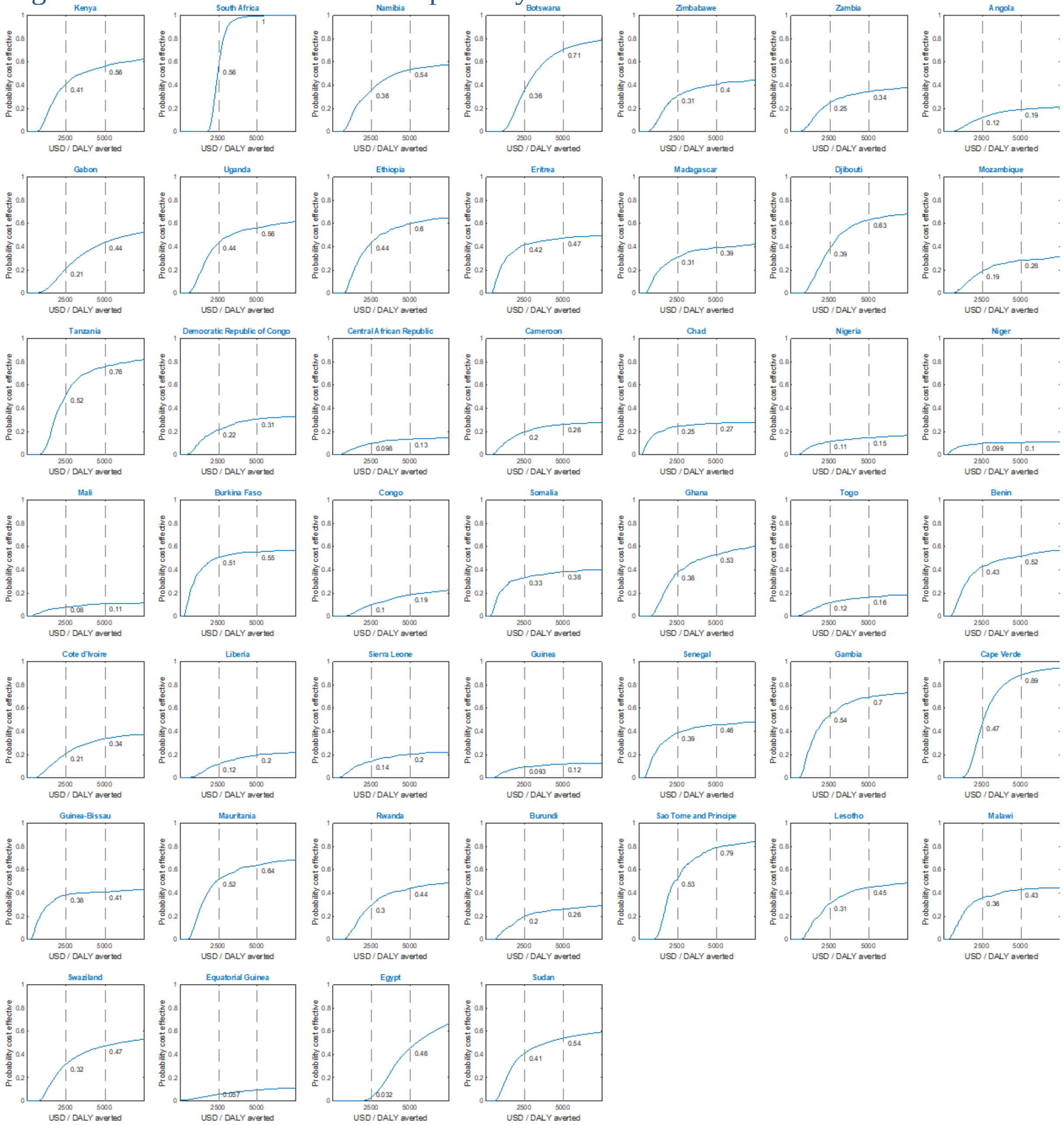

Figure Legend: CEACs for each country in Africa. Each CEAC plot shows the proportion of simulations (Y axis) where MNPs are cost effective at a particular cost effectiveness threshold value (X axis). Countries where the cumulative probability does not reach 1.0 are those in which the non-depicted simulations showed a net harm (and hence where cost-effectiveness could not be calculated).

Figure S4 Cost Effectiveness Acceptability Curves for Asia and The Middle East

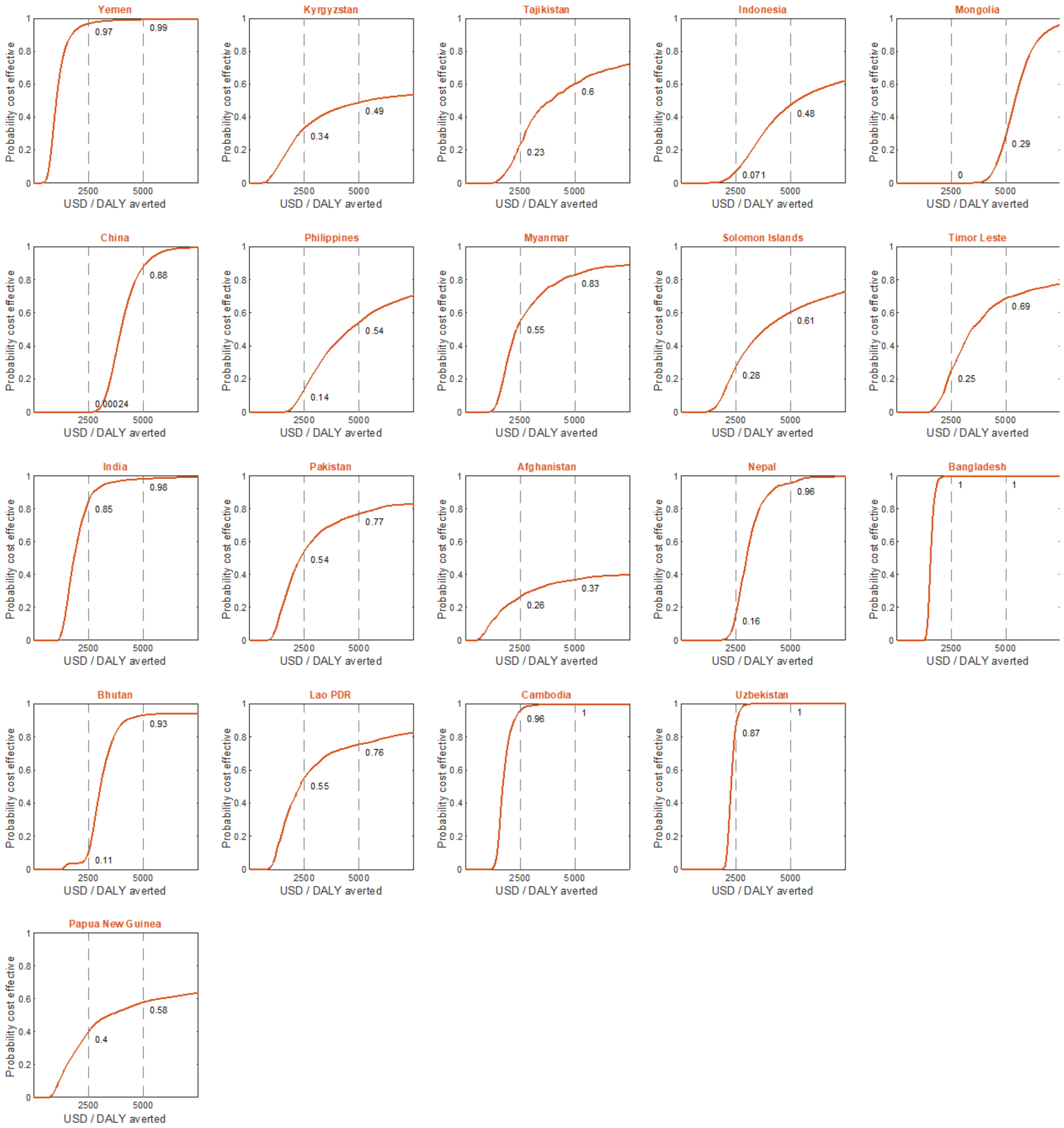

Figure Legend: CEACs for each country in Asia and the Middle East. Each CEAC plot shows the proportion of simulations (Y axis) where MNPs are cost effective at a particular cost effectiveness threshold value (X axis). Countries where the cumulative probability does not reach 1.0 are those in which the non-depicted simulations showed a net harm (and hence where cost-effectiveness could not be calculated).

Figure S5 Cost Effectiveness Acceptability Curves for Latin America

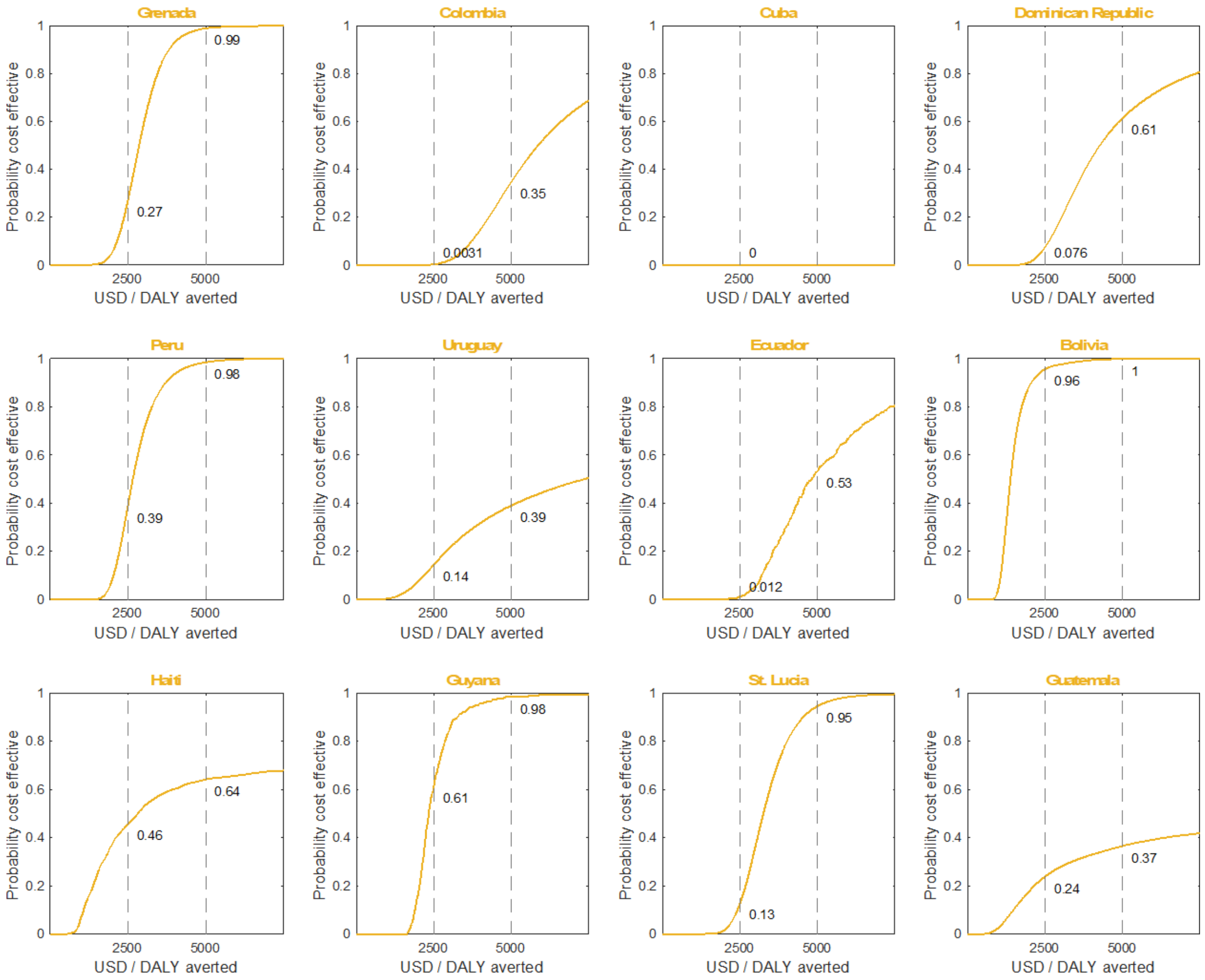

Figure Legend: CEACs for each country in Latin America. Each CEAC plot shows the proportion of simulations (Y axis) where MNPs are cost effective at a particular cost effectiveness threshold value (X axis). Countries where the cumulative probability does not reach 1.0 are those in which the non-depicted simulations showed a net harm (and hence where cost-effectiveness could not be calculated).

Figure S6 Cost per DALY averted using regional multipliers for programme costs

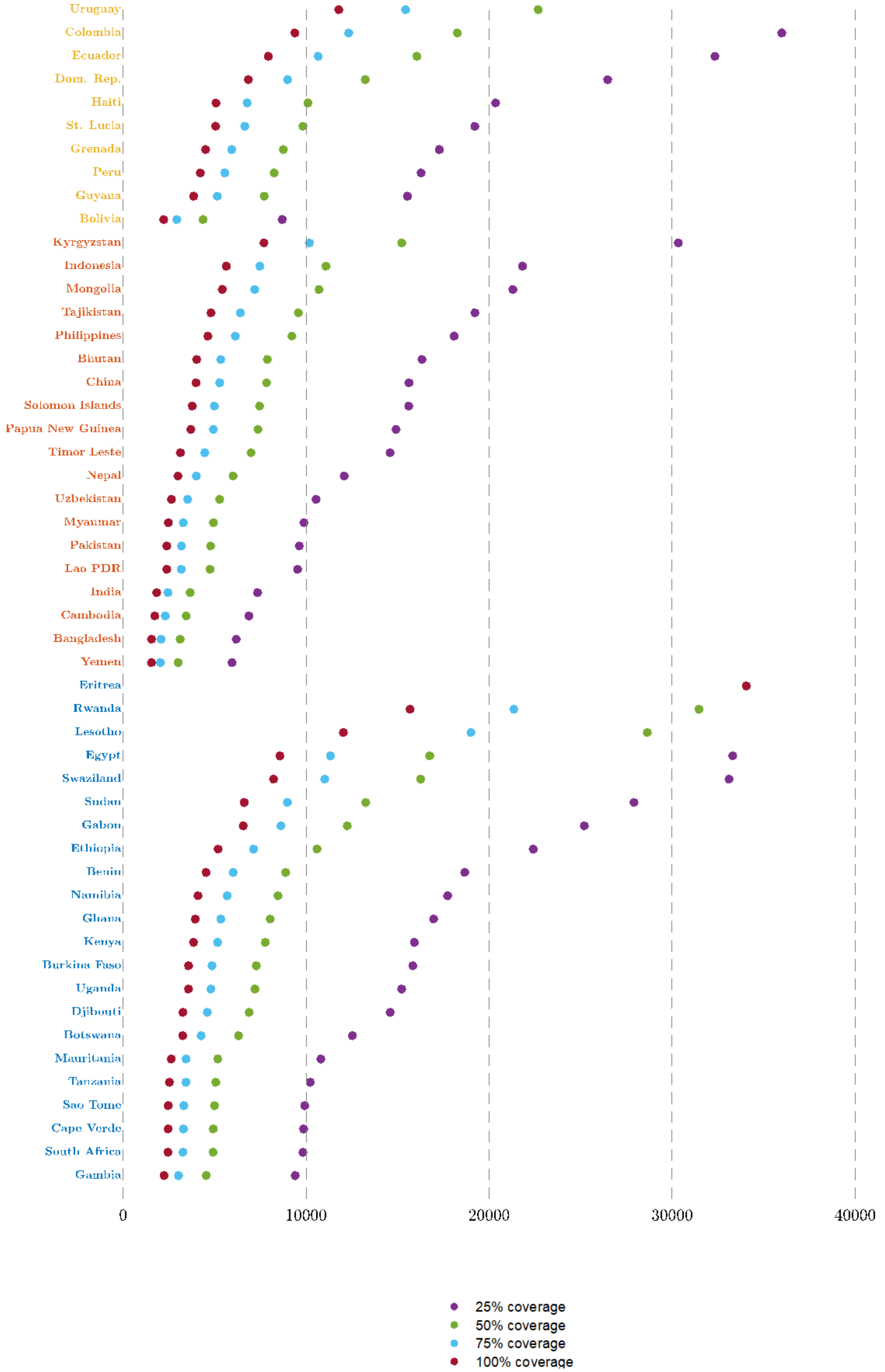

Figure S7 Correlation matrix of associations between YLDs/ YLLs/ DALYs averted by MNPs and prevalence of anaemia (mild/ moderate/ severe/ moderate+severe), incidence of malaria and incidence of diarrhoea.

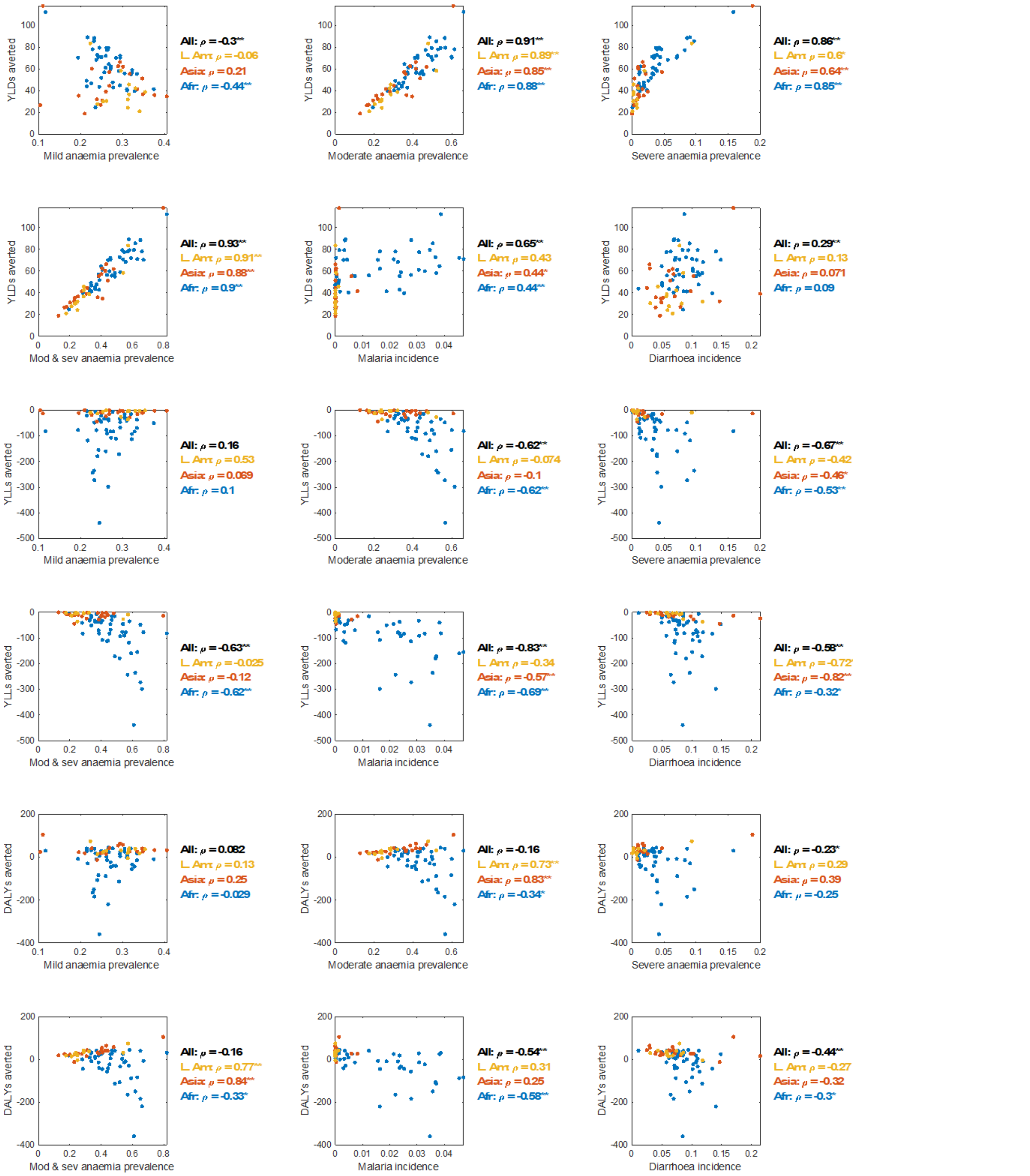

Figure S8 Correlation matrix of associations between prevalence of anaemia (mild/ moderate/ severe/ moderate+severe), incidence of malaria and incidence of diarrhoea.

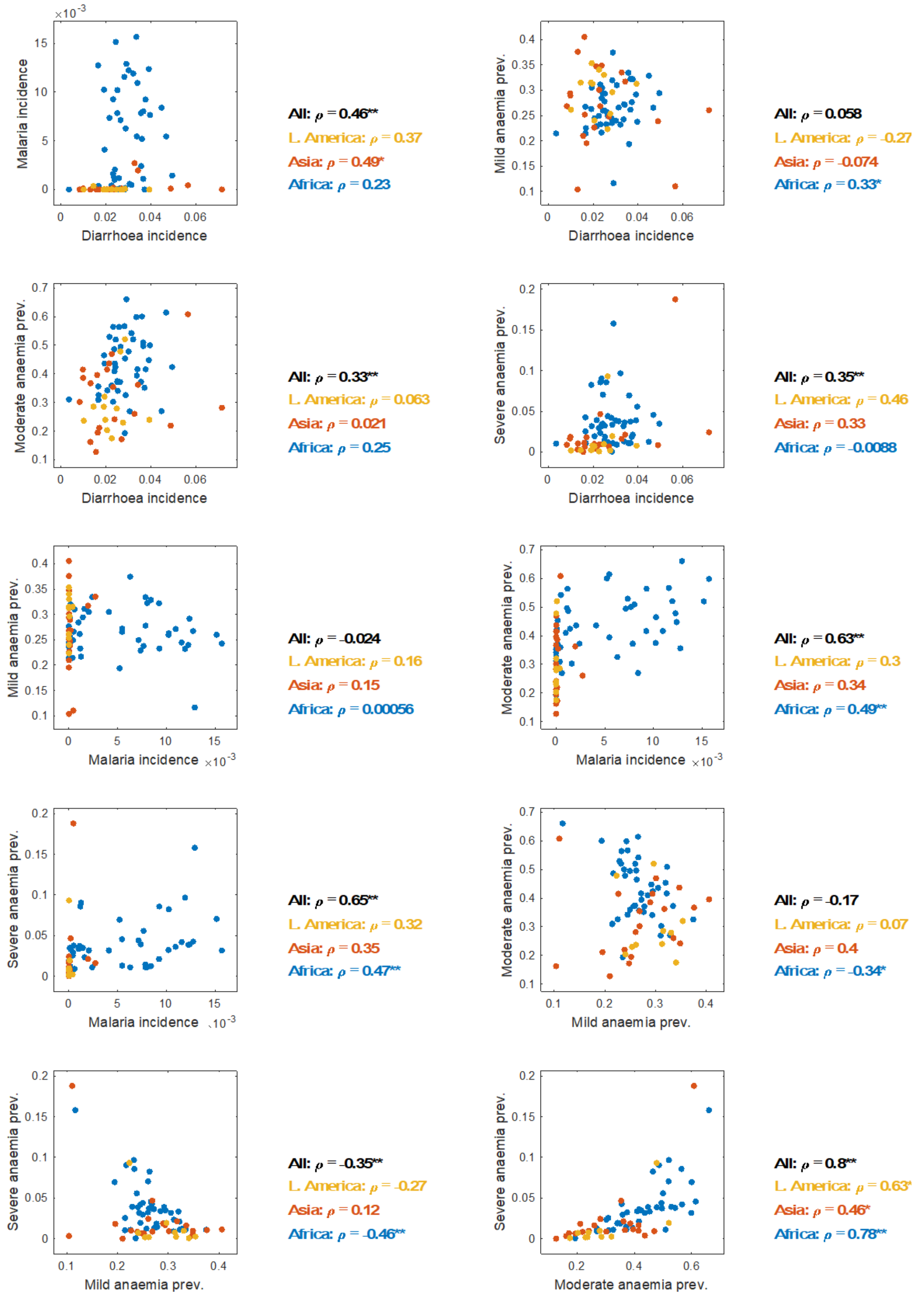

References

1. WHO. Daily iron supplementation in children 6-23 months of age. 2016. http://www.who.int/elena/titles/guidance_summaries/iron_children/en/ (accessed July 11th 2016).
2. WHO. WHO guideline: Use of multiple micronutrient powders for point-of-use fortication of foods consumed by infants and young children aged 6–23 months and children aged 2–12 years. Geneva: World Health Organization; 2016.
3. Husereau D, Drummond M, Petrou S, et al. Consolidated Health Economic Evaluation Reporting Standards (CHEERS) statement. *BMJ* 2013; **346**: f1049.
4. WHO. Haemoglobin concentrations for the diagnosis of anaemia and assessment of severity. Geneva: World Health Organization; 2011.
5. Jefferds ME, Irizarry L, Timmer A, Tripp K. UNICEF-CDC global assessment of home fortification interventions 2011: current status, new directions, and implications for policy and programmatic guidance. *Food Nutr Bull* 2013; **34**(4): 434-43.
6. World Health Organization. The global prevalence of anaemia in 2011. Geneva: World Health Organization; 2015.
7. Global Burden of Disease Collaborative Network. Global Burden of Disease Study 2017 (GBD 2017) Results. Seattle, United States
: Institute for Health Metrics and Evaluation (IHME); 2018.
8. Lamberti LM, Fischer Walker CL, Black RE. Systematic review of diarrhea duration and severity in children and adults in low- and middle-income countries. *BMC Public Health* 2012; **12**: 276.
9. Neuberger A, Okebe J, Yahav D, Paul M. Oral iron supplements for children in malaria-endemic areas. *Cochrane Database Syst Rev* 2016; **2**: CD006589.
10. UNICEF. Malaria: Children under 5 sleeping under an insecticide treated net (ITN) - Percentage. 2015. data.unicef.org (accessed June 2016).
11. World Health Organization. World Malaria Report 2014. Geneva: World Health Organization, 2015.
12. Lengeler C. Insecticide-treated bed nets and curtains for preventing malaria. *Cochrane Database Syst Rev* 2004; (2): CD000363.
13. Murray-Kolb LE, Khatry SK, Katz J, et al. Preschool micronutrient supplementation effects on intellectual and motor function in school-aged Nepalese children. *Arch Pediatr Adolesc Med* 2012; **166**(5): 404-10.
14. Pongcharoen T, DiGirolamo AM, Ramakrishnan U, Winichagoon P, Flores R, Martorell R. Long-term effects of iron and zinc supplementation during infancy on cognitive function at 9 y of age in northeast Thai children: a follow-up study. *American Journal of Clinical Nutrition* 2011; **93**(3): 636-43.
15. Pasricha S-R, Hayes E, Kalumba K, Biggs B-A. Effect of daily iron supplementation on health in children aged 4—23 months: a systematic review and meta-analysis of randomised controlled trials. *Lancet Global Health* 2013; **1**(2): e77 - e86.
16. Sachdev H, Gera T, Nestel P. Effect of iron supplementation on mental and motor development in children: systematic review of randomised controlled trials. *Public Health Nutr* 2005; **8**(2): 117-32.
17. Salomon JA, Haagsma JA, Davis A, et al. Disability weights for the Global Burden of Disease 2013 study. *Lancet Glob Health* 2015; **3**(11): e712-23.

18. Berger J, Dyck JL, Galan P, et al. Effect of daily iron supplementation on iron status, cell-mediated immunity, and incidence of infections in 6-36 month old Togolese children. *Eur J Clin Nutr* 2000; **54**(1): 29-35.
19. Soofi S, Cousens S, Iqbal SP, et al. Effect of provision of daily zinc and iron with several micronutrients on growth and morbidity among young children in Pakistan: a cluster-randomised trial. *Lancet* 2013.
20. Zlotkin S, Newton S, Aimone AM, et al. Effect of iron fortification on malaria incidence in infants and young children in Ghana: a randomized trial. *JAMA* 2013; **310**(9): 938-47.
21. UNICEF, WHO. Sources and prices of selected medicines for children - including therapeutic food, dietary vitamin and mineral supplementation. UNICEF, WHO; 2010.
22. Saskia De Pee, Rafael Flores-Ayala, Joris Van Hees, et al., editors. Introduction: Home Fortification Technical Advisory Group; 2013.
23. Horton S, Shekar M, McDonald C, Mahal A, Brooks JK. Scaling Up Nutrition - What Will It Cost? Washington DC: The World Bank; 2010.
24. Deasy A, Luneta V, Butler K. PO-0230 Management Of Severe Malaria In Children, An Audit Of Clinical Practise In A Rural Hospital In Zambia. *Archives of Disease in Childhood* 2014; **99**(S2).
25. World Health Organisation. Recommendations for management of common childhood conditions: Newborn conditions, dysentery, pneumonia, oxygen use and delivery, common causes of fever, severe acute malnutrition and supportive care. Geneva: World Health Organization; 2012.
26. World Health Organisation. Tables of Costs and Prices used in WHO-CHOICE Analysis. http://www.who.int/choice/costs/en/.
